# Identification of a photoredox-active Pt(IV) complex that induces light-mediated cell death

**DOI:** 10.1039/d5sc02879e

**Published:** 2025-09-01

**Authors:** Jevon W. Marsh, Lina Hacker, Sophie A. Twigger, Jake A. Vickery, Shitong Huang, Claudia Almuzara Romero, Aaron P. Langston, Ismael Diez-Perez, Rebecca A. Musgrave, Ester M. Hammond, Adam C. Sedgwick

**Affiliations:** a Chemistry Research Laboratory, University of Oxford Mansfield Road Oxford OX1 3TA UK; b Department of Oncology, University of Oxford, Old Road Campus Research Building Oxford OX3 7DQ UK; c Department of Chemistry, King's College London 7 Trinity Street London SE1 1DB UK adam.sedgwick@kcl.ac.uk

## Abstract

We report the synthesis and characterisation of a series of fluorogenic Pt(IV) complexes – CarboBlue, OxaliBlue and CisBlue. These Pt(IV) complexes were identified as photoactive, oxidising biomolecules under light irradation to then undergo rapid intramolecular photoreduction to release the fluorescent reporter, Nap-OH and corresponding Pt(II) species. OxaliBlue and CisBlue displayed cytotoxicity regardless of light irradiation. In contrast, HCT116 cells treated with CarboBlue displayed a light-dependent increase in fluorescence emission along with selective light-induced toxicity.

## Abstract 2

Here, we report the synthesis and study of the light-activated properties of the novel Pt(IV) complexes, CarboBlue, OxaliBlue and CisBlue. Only CarboBlue demonstrated selective light-mediated toxicity.

## Graphical abstract

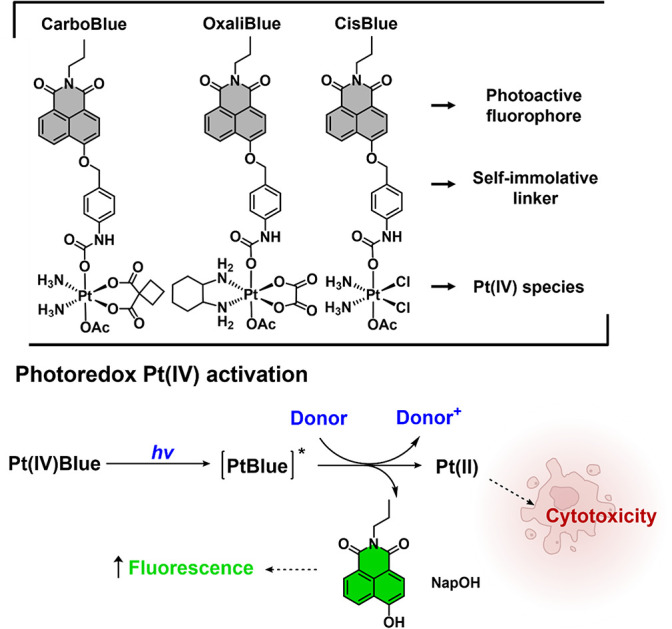

Pt(IV) complexes hold promise as potential cancer-selective alternatives to the FDA-approved Pt(II) chemotherapeutics, cisplatin, oxaliplatin, and carboplatin.^1,2^ It is widely considered that Pt(IV) complexes are non-toxic prodrugs that undergo intracellular reduction to afford the corresponding cytotoxic Pt(II) species. However, the exact mechanism for Pt(IV) reduction within cells remains poorly understood. Given that the mode of activation directly determines the selectivity and efficacy of these compounds, a comprehensive understanding of Pt(IV) redox chemistry within biology is needed. To address this knowledge gap, significant research efforts have been made towards identifying factors that contribute to Pt(IV) activation within cells.^3–6^ Notably, fluorogenic Pt(IV)-based probes have been developed by incorporating fluorophores at the axial positions.^7–9^ Reductive activation of these fluorogenic Pt(IV) complexes enables direct visualisation of intracellular Pt(IV) reduction *via* the release of the fluorescent axial ligand(s), affording an “off” to “on” fluorescence response.^7,10^ To this end, our group recently visualised the hypoxia-mediated reduction of fluorogenic Pt(IV) complexes (*e.g.*, CarboNap (Fig. 1)),^11,12^ which in turn has informed the design of so-called Hypoxia-Activated Pt(IV) Prodrugs.^11^ Hypoxia (low-oxygen concentrations) is a hallmark trait of many solid tumours,^13^ therefore this prodrug approach provides the potential to afford cancer-selective agents and overcome the systemic toxicity associated with traditional Pt(II) therapies.^14^ However, Pt(IV) prodrugs typically display slow reduction kinetics, which impacts the overall translational potential of this strategy.^15^ To increase our understanding on intracellular Pt(IV) activation rates, in this work, we developed a series of fluorogenic Pt(IV) complexes, named CarboBlue, OxaliBlue and CisBlue. Given that light irradiation is an established method for accelerating Pt(IV) activation,^4,16–19^ we investigated and compared both light exposure and intracellular oxygen levels as activation mechanisms. Among these complexes, CarboBlue emerged as a promising photoactivated chemotherapeutic, demonstrating light as the dominant activation pathway.

**Fig. 1:**
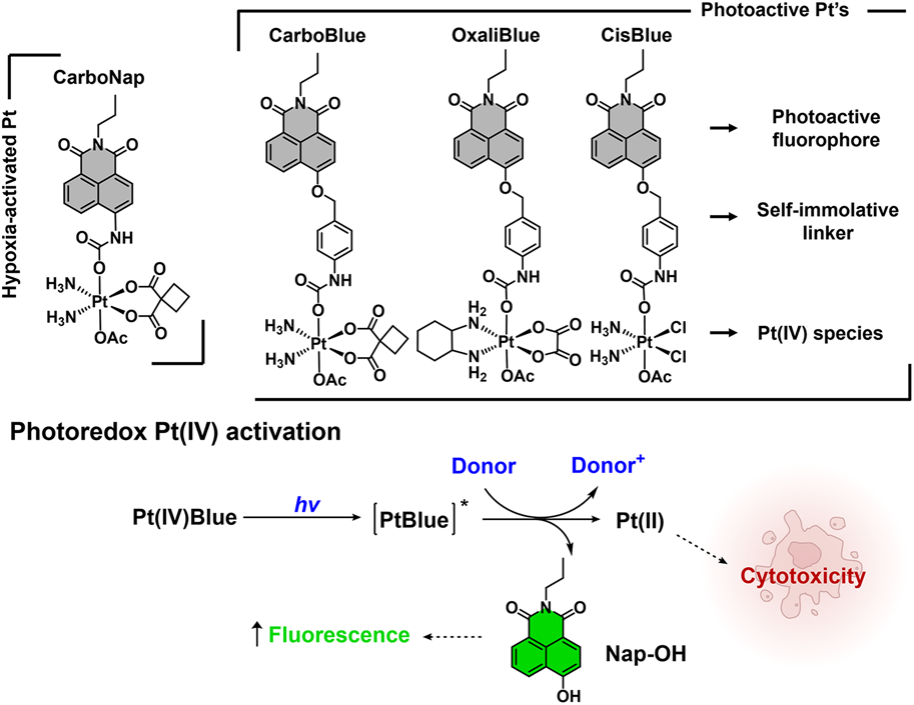
Top: chemical structures of hypoxia-activated CarboNap, and light-activated CarboBlue, OxaliBlue and CisBlue. Below: general schematic of the proposed mechanism of the photoreduction of Pt(IV) complexes, releasing fluorescent reporter Nap-OH and cytotoxic Pt(II) species.

In this study, the green-emitting fluorophore 4-hydroxy-*N*-propyl-1,8-naphthalimide (Nap-OH) was chosen. Several reports have demonstrated functionalisation of Nap-OH with stimuli-responsive self-immolative (traceless) linkers^20^ to afford ratiometric fluorescent probes with a measurable change from blue to green emission.^21,22^ This ratiometric feature is desirable as it would enable the potential monitoring of both Pt(IV) species (blue emission) and the release of Pt(II) species (green emission). In brief, Nap-OH was synthesised following an established synthetic protocol by Pfeffer and co-workers,^23^ and the desired fluorogenic Pt(IV) complexes, CarboBlue, OxaliBlue and CisBlue were isolated following established synthetic protocols (Schemes S1–S3).^24,25^ The full synthetic procedures and characterisation can be found in the SI.

While synthetic intermediate Nap-Bn-COOH (Scheme S1), displayed the expected blue emission, the Pt(IV)-functionality on CarboBlue, OxaliBlue and CisBlue resulted in significant fluorescent quenching of the blue fluorescence emission intensity (Fig. S1 and Scheme S4). This observed fluorescence quenching by Pt(IV) is attributed to the heavy-atom effect and the detection of singlet oxygen production from CarboBlue is suggestive of potential intersystem crossing of the singlet excited state (Fig. S2).^10,26^ Due to the quenching of the blue emissive states, we therefore focused on monitoring the changes in fluorescence emission at the expected 550 nm emission of Nap-OH with and without sodium ascorbate (NaAsc).^22^CarboBlue, OxaliBlue, and CisBlue were separately incubated with NaAsc (10 mM, 1 h) in PBS buffer (pH = 7.4, 0.01 M) and the fluorescence emission intensity at 550 nm was monitored every 10 minutes over a time period of 1 hour. Surprisingly, when compared to our previously reported hypoxia-activated CarboNap,^11^ which readily activates with NaAsc, only CisBlue showed a noticeable increase in fluorescence emission intensity (12.3 fold-change); minimal fluorescence changes were observed for OxaliBlue and CarboBlue (Fig. 2a and S3–S5). Considering these newly synthesised complexes lacked sensitivity towards NaAsc-mediated reduction, we wanted to understand the differences in reactivity compared to NaAsc-sensitive CarboNap.^11^ Cyclic voltammetry experiments were performed and the reduction potentials (cathodic peaks) of CarboBlue and OxaliBlue were measured in DMF as −0.35, and −0.21 *vs.* Fc^0^/Fc^+^, respectively (Fig. S6). Efforts to obtain a suitable reduction potential for CisBlue proved difficult due to solubility issues. However, in the instance of CarboNap under the same measurement conditions, a more positive reduction potential (−0.005 V *vs.* Fc^0^/Fc^+^) was observed supporting its observed greater sensitivity towards NaAsc-mediated reduction (Fig. S7).

**Fig. 2:**
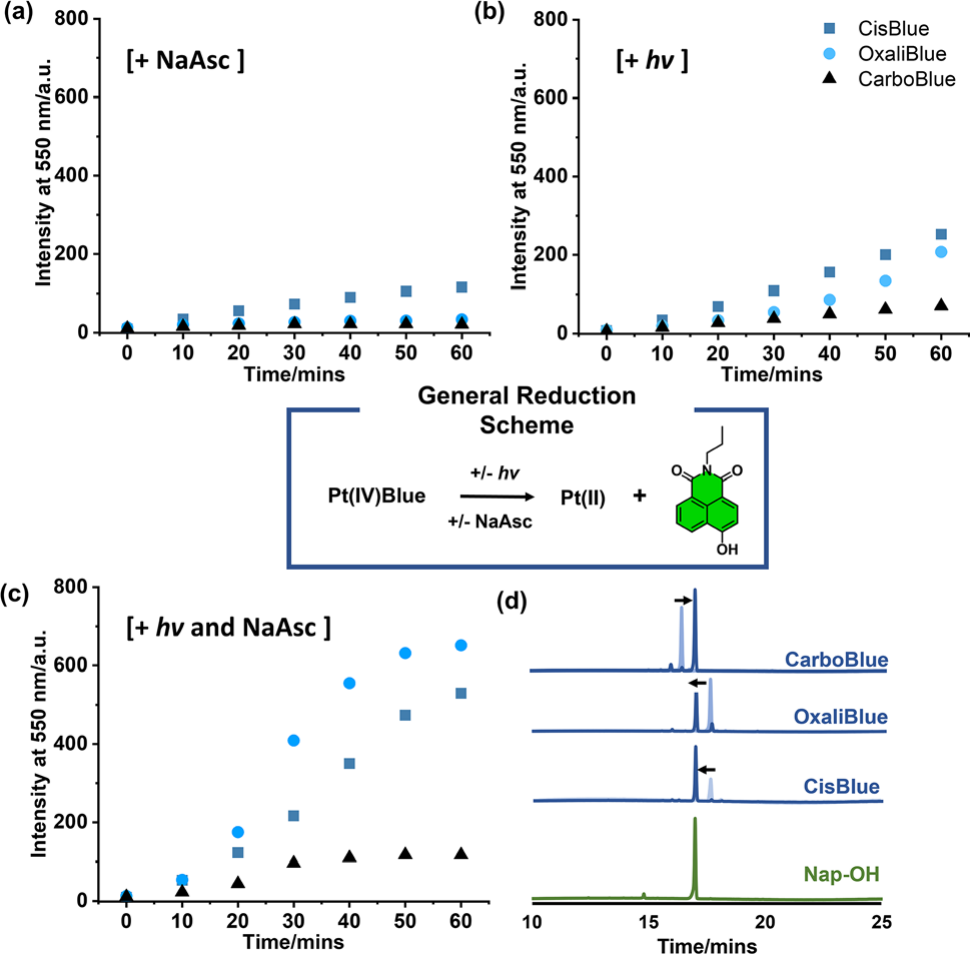
Changes in fluorescence emission intensity of CisBlue, OxaliBlue and CarboBlue (all 5 μM) at 550 nm over time (0, 10, 20, 30, 40, 50, and 60 min) (a) incubated with NaAsc (10 mM), (b) irradiated with blue light and (c) irradiated with blue light in the presence of NaAsc (10 mM). (d) HPLC chromatograms of Nap-OH (green), CisBlue, OxaliBlue and CarboBlue (all blue) (all 50 μM) irradiated with blue light and incubated with NaAsc (50 mM) for 1, 2 and 4 h, respectively, superimposed with their initial 0 h timepoint (shaded blue). All fluorescence spectra were performed in PBS (pH = 7.4, 0.01 M), *λ*_ex_ = 450 nm, slit widths: 10 nm and 5 nm; HPLC studies were performed in a H_2_O/MeCN (70/30%) solution.

Next, the reactivity of CarboBlue, OxaliBlue and CisBlue towards light-mediated reduction was tested. An aqueous solution (PBS, pH = 7.4, 0.01 M) of each complex (5 μM) was subjected to blue light irradiation (spectral width: 400–500 nm, 4 W, 1 h) and fluorescent measurements were studied over the same time course. As seen in Fig. 2b and S8–S10, increasing the length of light exposure time resulted in gradual increases in fluorescence emission intensities at 550 nm. In contrast, the co-treatment of light irradiation and NaAsc (10 mM) resulted in much greater changes in fluorescence emission when compared to light irradiation or NaAsc alone (Fig. 2c and S11–13), with a 54.2, 52.0 and 7.96-fold-change for CisBlue, OxaliBlue and CarboBlue, respectively. A trend in reactivity was observed; CarboBlue was found least reactive and required higher concentrations of NaAsc (50 mM) with light irradiation to observe noticeable increases in fluorescence emission (Fig. S11). This synergistic effect between light and NaAsc was further validated *via* UV-Vis spectroscopy (Fig. S14–S16) and HPLC analysis (Fig. 2d and S17–19). Minimal change to the fluorescence emission spectra and HPLC traces were seen in the absence of light and reductant, suggestive of good aqueous stability (Fig. S20–S25).

With the above data showcasing the potential of these complexes as promising photoactivated chemotherapeutics, we wanted to first determine the dark cytotoxicity of each complex (without light irradiation). In the absence of light, both CisBlue and OxaliBlue (10 μM) demonstrated significant cytotoxicity in a colorectal cancer cell line (HCT116). However, at the same concentration, CarboBlue appeared non-toxic after an incubation period of 64 h (Fig. S26). For these reasons, only CarboBlue was investigated further in this study.

In an effort to understand the photochemistry of CarboBlue, the excited-state reduction potential was estimated by combining the electrochemical and spectroscopic data (Fig. S27),^19,27^ which was calculated as 2.77 V *vs.* Fc^0^/Fc^+^. Interestingly, the excited-state reduction potential of previously reported CarboNap was calculated to be 2.86 V, which led to the identification of a similar light-mediated activation mechanism (Fig. S28 and S29). Time-dependent density functional theory (TD-DFT) calculations and natural transition orbitals (NTOs) identified excited states (>400 nm) for both CarboNap and CarboBlue corresponding to a ligand-to-metal charge transfer (LMCT)^28^ involving a ligand-based π orbital and the Pt dz^2^-based orbital, which supports the observed photomediated reduction mechanism for CarboNap and CarboBlue (See SI – computational analysis). Notably, the calculated excited-state properties of CarboBlue also suggested potential photooxidative behaviour, reinforcing the role of NaAsc as a sacrificial electron donor to facilitate and accelerate intramolecular photoreduction.^19,29^CarboBlue was therefore next tested with the known biological electron donor, NADH. The light irradiation of CarboBlue with increasing concentrations of NADH (0–4 mM) resulted in a dose-dependent increase in fluorescence emission intensity at 550 nm (blue lines: light + NADH, black line: light only, Fig. 3). High-resolution mass spectrometry and fluorescence spectroscopy (NADH emission) supported the potential photooxidation of NADH to NAD^+^*via*CarboBlue (Fig. S30–S32). Other biologically reducing species such as l-cysteine were also found to enhance the light-mediated increases in fluorescence emission intensities for CarboBlue (Fig. S33). Thus, with the known abundance of biological reductants in cancer cells,^30^ we believe these findings warranted testing CarboBlue as a potential phototherapeutic in cell studies.

**Fig. 3:**
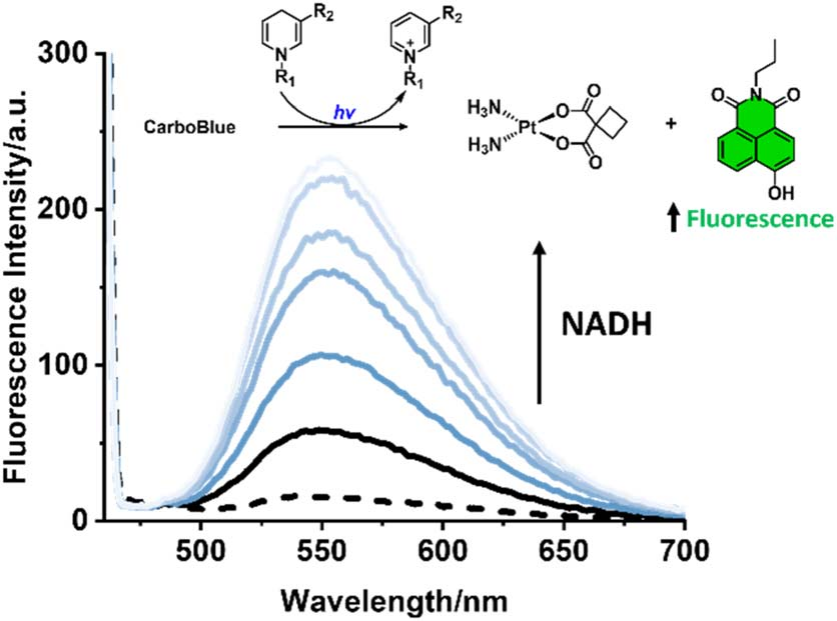
General reduction scheme of CarboBlue irradiated with blue light and NADH to release Pt(II), Nap-OH and NAD^+^. Fluorescence emission spectra of CarboBlue alone (dashed line); (ii) CarboBlue irradiated with blue light (black); and (iii) CarboBlue irradiated with blue light with increasing concentrations of NADH (0.05, 0.1, 1, 2, and 4 mM, blue) for 1 h in PBS (pH = 7.4, 0.01 M), *λ*_ex_ = 450 nm, slit widths: 10 nm and 5 nm.

Fluorescence imaging is crucial to understanding the influence of light and oxygen concentrations on the activation of CarboBlue within cells. As anticipated, HCT116 cells treated with CarboBlue and NaAsc (2 mM), followed by light irradiation (400–500 nm, 4 W, 30 min), resulted in significant enhancement in fluorescence emission intensity (determined by flow cytometry) (Fig. S34). We next wanted to determine and compare the ability of CarboBlue to undergo hypoxia-mediated reduction against light-mediated activation. HCT116 cells were treated with CarboBlue (2.5, 5, 10 μM) for 16 h at 21%, or < 0.1% O_2_ and then analysed *via* flow cytometry (Fig. 4a). Unlike our previously reported fluorogenic Pt(IV) complexes,^11,12^ no significant difference was observed in fluorescence emission intensity between 21% and <0.1% O_2_ concentrations, suggestive of no hypoxia-mediated activation. However, when HCT116 cells were treated with CarboBlue (10 μM) and irradiated with light under normoxic (21% O_2_) and hypoxic conditions (<0.1% O_2_), increases in fluorescent emission were observed regardless of oxygen concentrations (Fig. S35), indicating light as the most effective method for the activation of CarboBlue in cells.

**Fig. 4:**
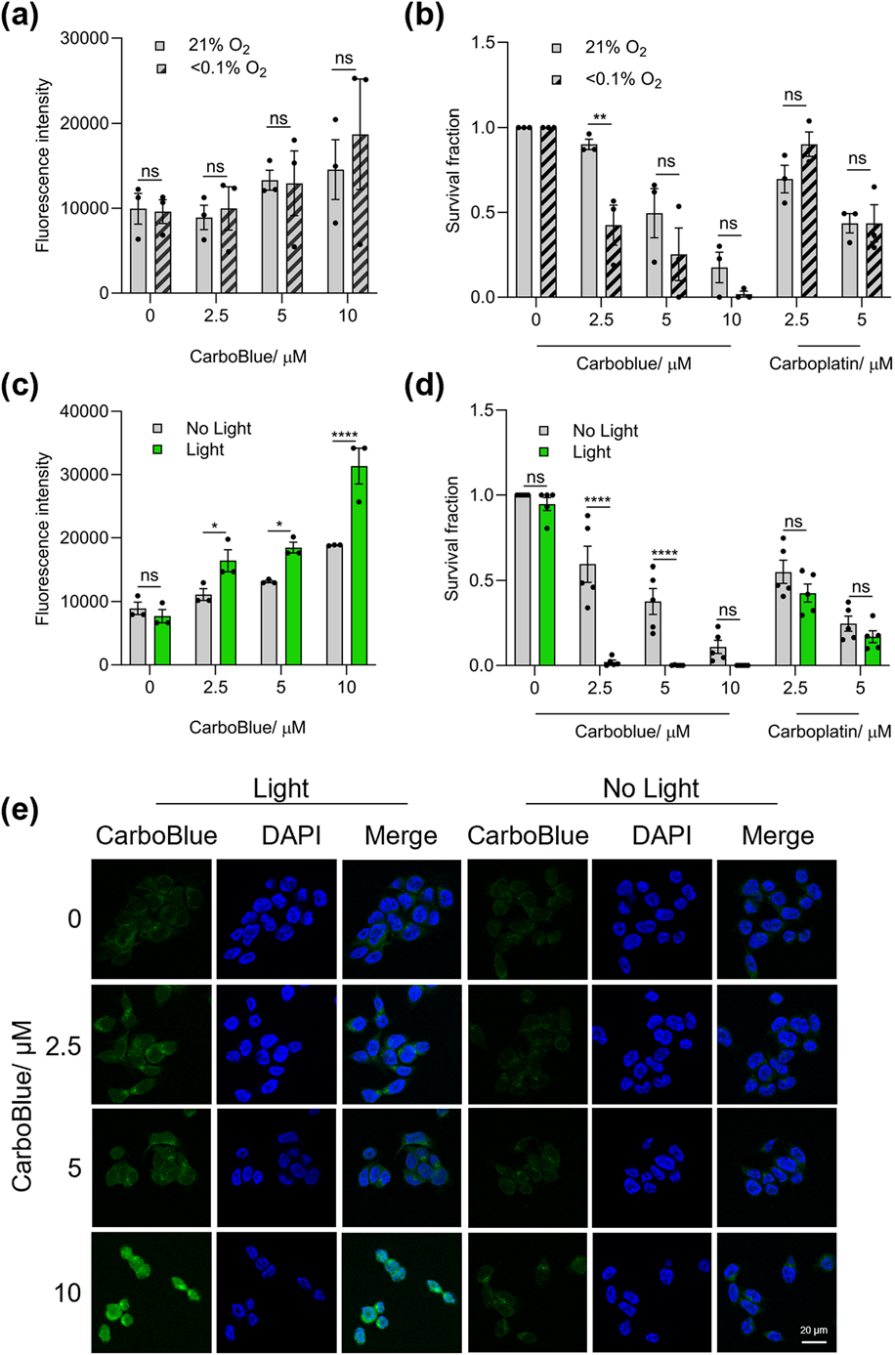
HCT116 cells treated with CarboBlue (0, 2.5, 5, 10 μM) for 16 h (a) under 21 or <0.1% O_2_ and analysed *via* flow cytometry (b) colony survival assay under 21 or <0.1% O_2_ and then incubated at 21% O_2_ (media changed after an additional 48 h) including carboplatin (2.5, 5 μM) (c) under 21% O_2_ and then exposed to blue light (>400 nm) (30 min) before analysis *via* flow cytometry – light is shown by green bar (d) under 21% O_2_ and then exposed to blue light (>400 nm) (30 min), media was then changed after an additional 48 h and a colony survival assay was undertaken (e) under 21% O_2_ and then exposed to blue light (400 nm) (30 min) before analysis *via* confocal microscopy, with representative pictures shown (scale bar = 20 μM). Data shown in a, b, c and d are *n* “≥” 3 and e is *n* = 1. Black dots on the graphs shown represent biological repeats (each of which was carried out in triplicate), and data is presented as mean + SEM. Statistical testing was done using two-way ANOVAs, where ns = non-significant, *p < 0.05, **p < 0.01, ****p < 0.0001.

While fluorescence analysis offers insight into the rate of Pt(IV) activation and a preliminary indication of the phototherapeutic potential of CarboBlue, accurate assessment of the efficacy of Pt-based therapeutics requires significantly longer evaluation periods (>24 hours).^31^ We therefore needed to determine whether the fluorescence response of CarboBlue exposed to light or hypoxic conditions correlated to cytotoxicity. Even though previously we had shown CarboBlue having minimal impact on cell viability, MTT assays do not accurately reflect long-term cell survival, as it primarily measures metabolic activity. Clonogenic assays are considered the gold standard for assessing long-term cell survival and provide a more comprehensive assessment of the long-term effects of Pt-based drugs.^32^ For these reasons, we evaluated the cytotoxicity of CarboBlue*via* a colony survival assay. HCT116 cells were treated with CarboBlue at various concentrations (2.5, 5, 10 μM) and conditions (hypoxia or blue light), and after 48 h, the plates were incubated at 21% O_2_ to allow colonies to form (14 days). While no hypoxia-mediated increase in fluorescence emission was observed, CarboBlue displayed modest hypoxia-induced cytotoxicity (Fig. 4b). In addition, with increasing concentrations of CarboBlue, an increase in dark toxicity was observed. Since imaging experiments are conducted over a much shorter timescale than colony survival assays (16 hours *vs.* 14 days), the observed increase in cytotoxicity suggests that intracellular reduction may occur progressively over the full duration of the 14 day study. Nonetheless, CarboBlue (2.5, 5, 10 μM) with light irradiation displayed significantly greater cytotoxicity (Fig. 4d). Confocal microscopy further confirmed the increase in fluorescence emission intensity of CarboBlue upon light irradiation (Fig. 4e) showcasing excellent intracellular photoactivation. Together with the synthetic versatility of the Nap-OH fluorescent scaffold, these findings support the potential of CarboBlue as a promising platform for optimisation as a photoactivated chemotherapeutic.^33^

In conclusion, we report the synthesis of novel light-activated Pt(IV) complexes—CarboBlue, OxaliBlue, and CisBlue. Functionalisation of the Nap-OH fluorophore with Pt(IV) resulted in pronounced quenching of blue fluorescence emission, suggestive of potential intersystem crossing (*via* singlet oxygen detection) and the initiation of an intramolecular photoredox process. TD-DFT calculations and analysis of natural transition orbitals (NTOs) identified a ligand-to-metal charge transfer (LMCT) band around ∼400 nm for CarboBlue. Comparable calculations also revealed an additional photo-mediated reduction pathway in our previously reported CarboNap. Upon light irradiation, CarboBlue-treated HCT116 cells exhibited fluorescence turn-on, consistent with intracellular activation. In parallel, CarboBlue demonstrated significant cytotoxicity under light irradiation, underscoring its potential as a photoactivated chemotherapeutic. Collectively, this study highlights the non-innocent nature of chromophores and proposes a broader design strategy for photoactivated Pt(IV) therapeutics through the simple conjugation of Pt(IV) complexes with known fluorophores.

## Author contributions

JWM synthesised all the compounds and carried out the solution experiments shown in the study. LH and SAT conducted all cellular experiments. JAV, SH and APL performed background experiments to support the direction of the project. CAR, IDP and RAM provided support for electrochemical and theoretical experiments. JWM, EMH and ACS designed the experiments and analysed the data. Both JWM and ACS contributed to writing the manuscript.

## Conflicts of interest

There are no conflicts to declare.

## Supplementary Material

SC-016-D5SC02879E-s001

## Data Availability

Data will be made available by the corresponding authors upon request. Supplementary information is available: Detailed procedures, characterisation, and NMR spectra are provided in the ESI. See DOI: https://doi.org/10.1039/d5sc02879e.
