# Supplementary material for: Identification of a photoredox-active Pt(IV) complex that induces light-mediated cell death
Source: Chem Sci. 2025 Sep 1;16(41):19187–91. doi: 10.1039/d5sc02879e (PMC12442281; doi:10.1039/d5sc02879e)
Supplement: SC-016-D5SC02879E-s001 [file SC-016-D5SC02879E-s001.pdf]

Supplementary Information (SI) for Chemical Science.
This journal is © The Royal Society of Chemistry 2025

Supporting Information

Identification of a photoredox-active Pt(IV) complex that induces light-mediated cell death

Jevon W. Marsh,[a,†] Lina Hacker,[b,†] Sophie A. Twigger,[b,†] Jake A. Vickery,[c] Shitong Huang,[a] Claudia Almuzara Romero,[c] Aaron P. Langston,[c] Ismael Diez-Perez,[c] Rebecca A. Musgrave,[c] Ester M. Hammond*[b] and Adam C. Sedgwick*[a,c]

[a]Chemistry Research Laboratory, University of Oxford, Mansfield Road, OX1 3TA, United Kingdom

[b]Department of Oncology, University of Oxford, Old Road Campus Research Building, Oxford, OX3 7DQ, United Kingdom

[c]Department of Chemistry, King's College London, 7 Trinity Street, London, SE1 1DB, United Kingdom

Emails: ester.hammond@oncology.ox.ac.uk and adam.sedgwick@kcl.ac.uk

Table of Contents

1. **General Information and Methods** **S3**

2. **Synthetic Schemes and Procedures** **S6**

3. **Additional Analyses** **S16**

4. **NMR and HRMS Spectra** **S50**

5. **Computational Details** **S71**

6. **References** **S79**

1. General Information and Methods

All chemicals and reagents were of analytical grade and purchased commercially. Fluorescence and Absorbance spectra were collected on a PerkinElmer LS55 Luminescence spectrometer and Jasco V-770 spectrophotometer, respectively, using quartz cuvettes (1 cm path length). Complexes were purified by column chromatography on Merck® silica gel 60 under a positive pressure of N_2 and eluent ratios are reported as volume percentages. NMR spectra were recorded on Bruker AVIII 400, Bruker AVII 500 (with cryoprobe) and Bruker AVIII 500 spectrometers, and chemical shifts are reported as δ values in ppm. HPLC analyses was carried out using a Thermo Fisher Scientific Vanquish HPLC system with an Ascentils C-18 column [15 cm x 4.6 mm, 5 µm]; HPLC conditions: injection volume: 40 µL, flow rate: 1.5 mL/min, 25 °C, 0–35 min [2:98 MeCN:H_2O (both with 0.1 % acetic acid), 5 min hold → 100% MeCN with 0.1 % acetic acid for 20 min, 2 min hold → 2:98 MeCN:H_2O (both with 0.1% acetic acid), 2 min → 2:98 MeCN:H_2O (both with 0.1 % acetic acid), 6 min hold]. High Resolution Mass Spectrometry (HRMS) spectra were obtained using Waters Micromass LCT and Bruker microTOF spectrometers. For analytical work, all complexes investigated were dissolved in DMSO to prepare 5 mM stock solutions. Each stock solution was then diluted in PBS (pH 7.4, 0.01 M). A solution of PBS was made by dissolving one tablet (Sigma Aldrich) in H_2O (200 mL) to yield 0.01 M phosphate buffer, 0.0027 M potassium chloride and 0.137 M sodium chloride solution. Fluorescence analysis used 5 µM working solutions of each probe. A 70/30 ratio of milli-Q water/MeCN mixture was used to provide 50 µM working solutions of each probe for HPLC analysis. Light irradiation studies were performed 5 cm away from the light source in quartz cuvettes for the relevant time points with a 4 Watts Blue GU10 LED bulb (spectra width 400 – 500 nm) held in a standard desktop lamp. Singlet oxygen sensor green was purchased from Invitrogen™.

1.1. Electrochemical experiments

Electrochemical experiments were carried out using an Autolab IMP PGSTAT128N potentiostat (Metrohm, Netherlands) equipped with a three-electrode configuration comprised of two 3 mm glassy carbon working electrodes and a platinum wire counter electrode, with ferrocene (10 mM) as a reference. The working electrodes were polished before each scan with alumina and deionised water prior to being immediately transferred to

a 1 mM solution of each compound in DMF. Prior to each run, the electrolyte solutions were deaerated with N_2. A total volume of 2 mL was used in each experiment, consisting of 1 mM of compound and 200 mM of Tetrabutylammonium hexafluorophosphate.

1.2. Cell Culture

Human colorectal carcinoma cells HCT116 (Prof. Bert Vogelstein, John Hopkins, USA) were cultured in DMEM supplemented with 10 % FBS in humified incubators at 37 °C with 5 % CO_2. Cells were verified to be negative for mycoplasma using a Lonza MycoAlert® mycoplasma detection assay (LT07-318, Lonza). For experiments where light irradiation was used, cells were seeded in FluorBrite™ DMEM (Gibco, A1896701) to circumvent absorption of light <450 nm by phenol red. Chemical reduction experiments were performed using sodium ascorbate (Sigma Aldrich).

1.3. Hypoxia exposure

Cells were exposed to < 0.1 % O_2 using a Bactron II anaerobic chamber (Shel Labs). Glass dishes were used where possible for hypoxic experiments. The oxygen concentration of the chamber was periodically checked using anaerobic oxygen indicator strips (Thermo Fisher) and an OxyLite (Oxford Optronix).

1.4. Flow Cytometry

Cells were treated with **CarboBlue** (2.5, 5, 10 μM) and exposed to either 21 % O_2, < 0.1% O_2 (16 h), NaAsc (2 mM, 2 h) and then exposed to blue light (30 min) if required. Cells were then washed with PBS, scraped into PBS the pellet was collected. The cell pellet was then fixed with 4% PFA (10 min), washed twice with PBS and diluted in fresh PBS. Samples were then analysed on a CytoFLEX flow cytometer (Beckman Coulter) using CytExpert software, with the FITC filter set (excitation 488 nm/ emission 525/540 nm). Analysis was carried out for a cell count of 10,000 events for each treatment condition in triplicate. FloJo software (BD Biosciences was used to analyse the data.

1.5. Colony Survival Assay

Cells were seeded at a density of 200 cells/ well in 6-well plates, to give a plating efficiency of >50% for the untreated condition at the experiment end. Each treatment condition was performed in triplicate. After treatment (**CarboBlue**/ Carboplatin/ hypoxia/ blue light), the media of the cells was replaced after an additional 48 h, and then the plates were incubated at 21% O_2 to allow colonies to form (14 days). The media was removed, and they were stained with crystal violet (0.5% w/v in 50% MeOH and 20% EtOH) for 1 h, before washing with water. Colonies were counted manually (cell counter, Stuart Scientific) and the survival fraction was calculated by dividing the no. of colonies counted for the treatment condition by the number of cells seeded multiplied by the plating efficiency for the untreated control.

1.7. MTT Assay

Cells were seeded in 96 well plates (3000 cells/well) and allowed to adhere. After treatment with **CarboBlue** at the indicated concentrations (16 h), the cells were exposed to blue light (30 min). After an additional 48 h, MTT reagent (0.5 mg/mL, Invitrogen) was added for 3h (37 ^{o}C) with the plate protected from light. MTT containing media was then removed and formazan crystals were solubilised with DMSO (100 μL, 15 min, 37 ^{o}C), protected from light. The absorbance was read at 570 nm (POLARstar, BMG LabTech). Data are shown as percentage viability relative to the untreated control.

1.8. Fluorescent Microscopy

Cells were seeded into chamber slides and treated with **CarboBlue** (2.5, 5, 10 μM) before exposure to blue light (30 min) or no exposure. The cells were then fixed with 4 % PFA (10 min), washed three with PBS, Hoechst (Sigma Aldrich) stained and mounted with Prolong^{TM} Diamond Antifade Mountant (Invitrogen). Cells were visualised with an LSM710 confocal microscope (Carl Zeiss Microscopy Ltd) at 60x magnification.

2. Synthetic Schemes and Procedures

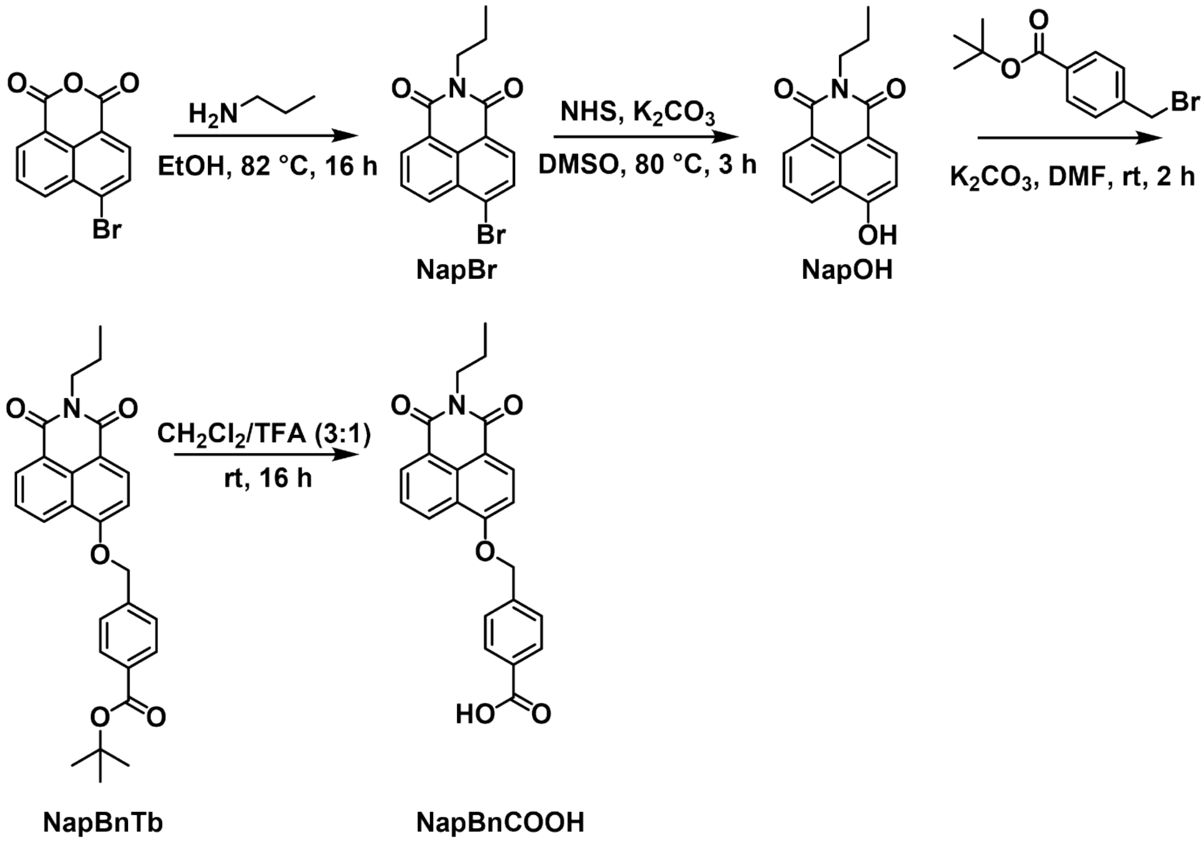

Scheme S1. Synthetic route showing access to the key intermediate **Nap-Bn-COOH**.

6-Bromo-2-propyl-1H-benzo[de]isoquinoline-1,3(2H)-dione (Nap-Br)

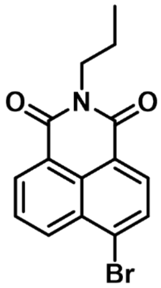

Following previously published procedures,[1,2] 4-Bromo-1,8-naphthalic anhydride (1 g, 3.67 mmol) was suspended in ethanol (14 mL) and propylamine (235 mg, 3.97 mmol, 1.1 eq) was added. The reaction was refluxed for 16 h and then cooled to room temperature to precipitate the desired product that was collected by vacuum filtration and rinsed with diethyl ether to yield **Nap-Br** as a beige solid (597 mg, 52 % yield). The product was characterised by ^{1}H NMR spectroscopy, was consistent with literature reports and used in subsequent reactions without further purification.[1] ^{1}H NMR (400 MHz, $CDCl_3$) δ (ppm) 8.66 (dd, *J* = 7.3, 1.2 Hz, 1H), 8.57 (dd, *J* = 8.5, 1.2 Hz, 1H), 8.42 (d, *J* = 7.9 Hz, 1H), 8.05 (d, *J* = 7.9 Hz, 1H), 7.85 (dd, *J* = 8.5, 7.3 Hz, 1H), 4.18 – 4.10 (m, 2H), 1.84 – 1.70 (m, 2H), 1.02 (t, *J* = 7.4 Hz, 3H).

6-hydroxy-2-propyl-1H-benzo[de]isoquinoline-1,3(2H)-dione (Nap-OH)

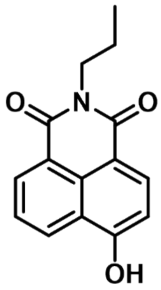

Nap-OH was synthesised according to previous published procedures.[3] **Nap-Br** (1.13 g, 3.66 mmol) was dissolved in DMSO (15 mL) and *N*-hydroxysuccinimide (NHS, 463 mg, 4.02 mmol, 1.10 eq) and K_2CO_3 (1.67 g, 12.08 mmol, 3.3 eq) were added. The reaction was heated to 80 °C for 3 h, cooled to room temperature and diluted with H_2O (150 mL). The solution was acidified with 1 M HCl to pH 1.0, which resulted in the precipitation of the desired product. The solid was filtered, washed with water and dried to yield **Nap-OH** (1.51 g) as a brown solid (quantitative). The product was characterised by ^{1}H NMR spectroscopy and was consistent with literature reports and used in subsequent reactions without further purification.[3] ^{1}H NMR (400 MHz, DMSO-d_6) δ (ppm) 11.92 (s, 1H), 8.50 (ddd, *J* = 24.2, 7.8, 1.2 Hz, 2H), 8.35 (d, *J* = 8.2 Hz, 1H), 7.76 (dd, *J* = 8.4, 7.3 Hz, 1H), 7.18 (d, *J* = 8.2 Hz, 1H), 4.02 – 3.94 (m, 2H), 1.70 – 1.56 (m, 2H), 0.91 (t, *J* = 7.4 Hz, 3H). **HRMS** $[M+H]^+$: expected 294.0737; observed 294.0741.

***tert*-Butyl-4-(((1,3-dioxo-2-propyl-2,3-dihydro-1H-benzo[de]isoquinolin-6-yl)oxy)methyl)benzoate (Nap-Bn-Tb)**

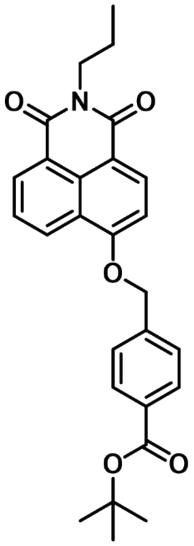

Nap-OH (1.62 g, 6.34 mmol) was dissolved in DMF (10 mL) and 4-Bromomethylbenzoic acid tert-butyl ester (1.89 g, 6.97 mmol, 1.1 eq.) and K_2CO_3 (2.63 mg, 19.02 mmol, 3 eq.) was added. The reaction was stirred at room temperature and monitored by TLC. Once deemed complete, the reaction was diluted with EtOAc (50 mL) and brine (50 mL); the organic layer was collected and dried over $MgSO_4$ and the solvent reduced to obtain a beige solid (1.60 g, 57 %). ^{1}H NMR (400 MHz, $CDCl_3$) δ (ppm) 8.65 – 8.58 (m, 2H), 8.53 (d, *J* = 8.3 Hz, 1H), 8.10 – 8.02 (m, 2H), 7.72 (dd, *J* = 8.3, 7.4 Hz, 1H), 7.60 – 7.53 (m, 2H), 7.09 (d, *J* = 8.3 Hz, 1H), 5.43 (s, 2H), 4.18 – 4.09 (m, 2H), 1.83 – 1.69 (m, 2H), 1.61 (s, 9H), 1.01 (t, *J* = 7.5 Hz, 3H); ^{13}C NMR (101 MHz, $CDCl_3$) δ (ppm) 165.42, 164.64, 164.04, 159.56, 140.13, 133.35, 132.36, 131.80, 130.11, 129.62, 128.69, 127.14, 126.29, 123.76, 122.71, 115.77, 106.59, 81.47, 77.48, 77.16, 76.84, 70.36, 41.97, 28.33, 21.56, 11.67. **HRMS** $[M+H]^+$: expected 446.1962; observed 446.1970.

4-(((1,3-dioxo-2-propyl-2,3-dihydro-1H-benzo[de]isoquinolin-6-yl)oxy)methyl)benzoic acid (NapBnCOOH)

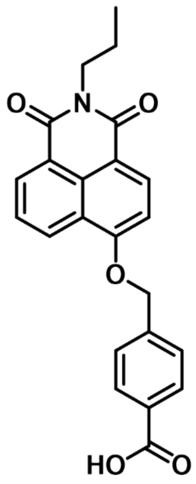

NapBnTb (400 mg, 0.90 mmol) was dissolved in CH_2Cl_2 (20 mL) and trifluoroacetic acid (6 mL) was added. The solution was stirred at room temperature and monitored by TLC. Once complete, the solvent was removed under reduced pressure and the solid rinsed with toluene to yield a beige solid (320 mg, 91 %). The product was used in subsequent reactions without further purification. ^{1}H NMR (400 MHz, DMSO-d$_6$) δ (ppm) 8.65 (dd, *J* = 8.4, 1.2 Hz, 1H), 8.53 (dd, *J* = 7.3, 1.2 Hz, 1H), 8.48 (d, *J* = 8.3 Hz, 1H), 8.02 (d, *J* = 8.2 Hz, 2H), 7.86 (dd, *J* = 8.4, 7.3 Hz, 1H), 7.71 (d, *J* = 8.1 Hz, 2H), 7.44 (d, *J* = 8.4 Hz, 1H), 5.59 (s, 2H), 4.00 (dd, *J* = 8.4, 6.5 Hz, 2H), 1.65 (q, *J* = 7.5 Hz, 2H), 0.92 (t, *J* = 7.4 Hz, 3H); ^{13}C NMR (101 MHz, DMSO-d$_6$) δ (ppm) 167.05, 163.55, 162.90, 159.00, 140.98, 133.07, 131.12, 130.53, 129.63, 128.62, 128.35, 127.46, 126.58, 122.90, 121.93, 114.59, 107.48, 69.74, 41.04, 40.15, 39.93, 39.84, 39.73, 39.52, 39.31, 39.10, 38.89, 20.89, 11.38. **HRMS** $[M+H]^+$: expected 390.1336; observed 339.1329.

General procedure for the oxidation of Platinum(II) to Platinum(IV)

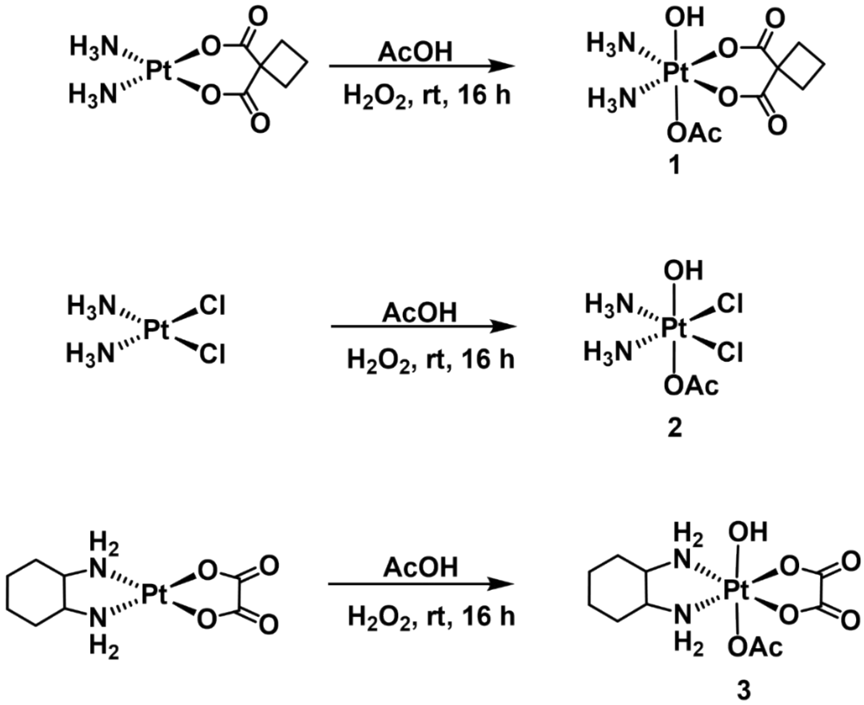

Scheme S2. Synthesis of CarboPt(IV)(OH)(OAc) (**1**, Top), CisPt(IV)(OH)(OAc) (**2**, middle) and OxaliPt(IV)(OH)(OAc) (**3**, bottom).

CisPt(IV)(OH)(OAc), OxaliPt(IV)(OH)(OAc) and CarboPt(IV)(OH)(OAc) (complexes **1**–**3**, respectively) were synthesised according to literature procedures (**Scheme S2**).[4] The corresponding Pt(II) precursors (300 mg of either cisplatin(II), oxaliplatin(II) or carboplatin(II)) were suspended in acetic acid (200 mL) and aq. H_2O_2 (4 mL, 30% w/w; 50% w/w for carboplatin(II)) and stirred at room temperature for 16 h in the absence of light. The solvent was then removed under reduced pressure to obtain a yellow oil, to which cold ethanol and diethyl ether was added to induce precipitation. The obtained solid was then rinsed with diethyl ether and acetone, centrifuged and decanted to yield the desired product. All complexes were characterised by ^{1}H NMR spectroscopy, were consistent with literature reports and used in subsequent reactions without further purification.

General procedure for the synthesis of Pt(IV)-fluorophore conjugates

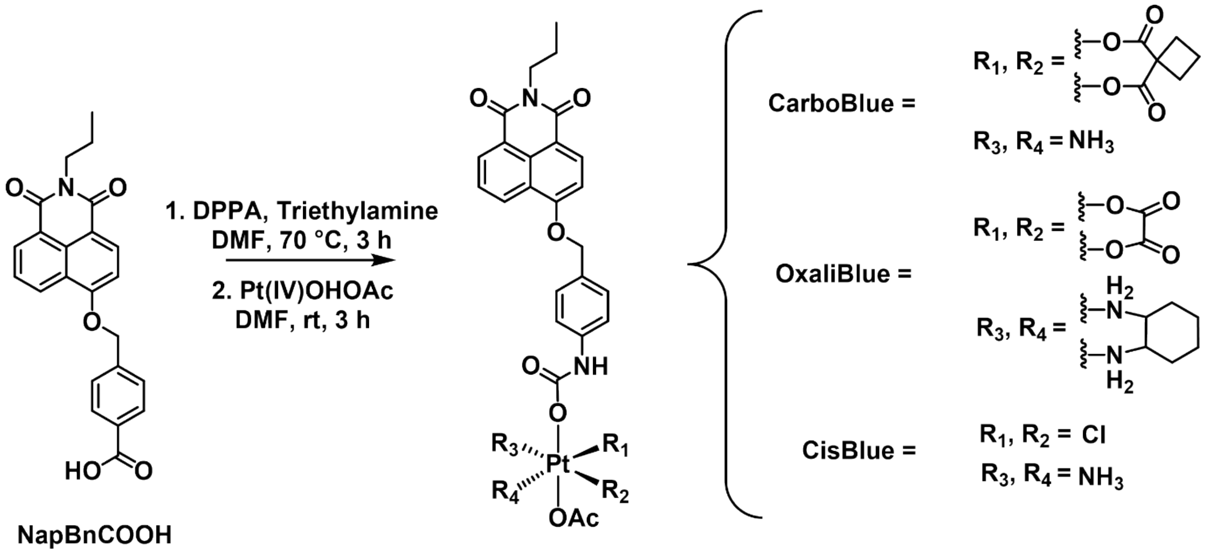

Scheme S3. General synthetic scheme for **CarboBlue** (top), **OxaliBlue** (middle) and **CisBlue** (bottom).

CarboBlue, **OxaliBue** and **CisBlue** were synthesised following a modified protocol based on a previous report from our group.[4] **Nap-Bn-COOH** (62 mg, 0.16 mmol) was dissolved in DMF (2 mL) and DPPA (131 mg, 0.477 mmol, 3 eq.) and triethylamine (66 µL, 0.477 mmol, 3 eq.) was added and the suspension was stirred at room temperature for 30 minutes, then gradually increased to 70 °C for 3 h, where it became a clear brown solution. The solution was allowed to cool to room temperature to yield a suspension before the relevant Pt(IV)(OH)(OAc) (1 eq.) was added and the temperature gradually increased to 70 °C and monitored by TLC. After *ca.* 3 h, the dark brown solution was cooled to room temperature and immediately added to a column on silica gel (100% Hexane → 100 % EtOAc→ 100 % CH_2Cl_2 → 10 % MeOH in CH_2Cl_2) to obtain the product which was then triturated with ***minimal*** cold MeOH yielding a yellow solid. Note - all complexes have some solubility in MeOH; yields reported are after trituration.

[Pt(NH$_3$)$_2$(CBDCA)(4-(((1,3-dioxo-2-propyl-2,3-dihydro-1H-benzo[de]isoquinolin-6-yl)oxy)methyl)phenyl)carbamic acid)(OAc)] (CarboBlue)

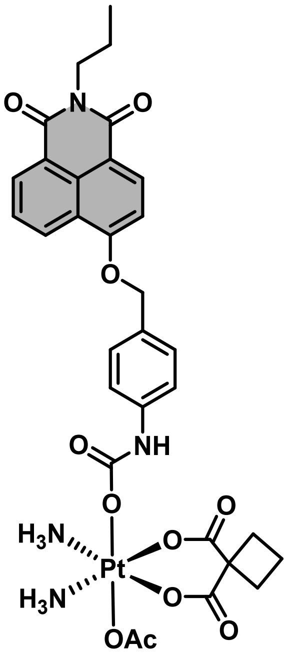

Yellow solid (34 mg, 26 % yield). ^{1}H NMR (400 MHz, DMSO-d$_6$) δ (ppm) 9.13 (s, 1H), 8.57 – 8.43 (m, 3H), 7.80 (t, *J* = 7.9 Hz, 1H), 7.52 – 7.37 (m, 5H), 6.53 (br, 6H), 5.36 (s, 2H), 4.00 (t, *J* = 7.6 Hz, 2H), 2.58 (t, *J* = 8.1 Hz, 2H), 2.45 (t, *J* = 8.2 Hz, 2H)*, 1.87 – 1.78 (m, 2H), 1.64 (q, *J* = 7.4 Hz, 2H), 0.91 (t, *J* = 7.4 Hz, 3H); *paritally overlapped with DMSO peak. ^{13}C NMR (101 MHz, DMSO-d$_6$) δ (ppm) 176.18, 163.62, 162.96, 159.46, 140.80, 133.19, 131.09, 128.65, 128.58, 128.45, 128.33, 126.46, 123.00, 121.90, 118.08, 114.26, 107.46, 70.57, 55.41, 41.04, 32.85, 29.51, 22.46, 20.91, 15.75, 11.40. ^{195}Pt NMR (86 MHz, DMSO-d$_6$) δ (ppm) 1968. **HRMS** [M+H]$^+$: expected 834.1945; observed 834.1914.

[Pt(DACH)(ox)(4-(((1,3-dioxo-2-propyl-2,3-dihydro-1H-benzo[de]isoquinolin-6-yl)oxy)methyl)phenyl)carbamic acid)(OAc)] (OxaliBlue)

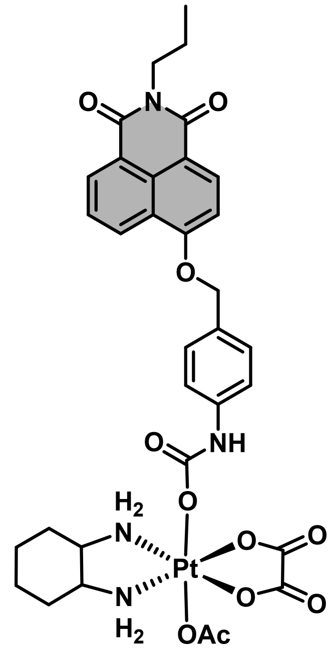

Yellow solid (29 mg, 21 % yield). ^{1}H NMR (400 MHz, DMSO-d$_6$) δ (ppm) 9.24 (s, 1H), 8.89 (s, 1H), 8.56 – 8.41 (m, 5H), 8.30 (s, 1H), 7.80 (t, *J* = 7.9 Hz, 1H), 7.49 – 7.39 (m, 5H), 5.37 (s, 2H), 3.99 (t, *J* = 7.6 Hz, 2H), 2.73 (s, 1H), 2.62 (s, 1H), 2.15 (d, *J* = 12.1 Hz, 2H), 1.97 (s, 3H), 1.64 (q, *J* = 7.5 Hz, 2H), 1.50 (s, 4H), 1.18 (s, 2H), 0.91 (t, *J* = 7.4 Hz, 3H). ^{13}C NMR (151 MHz, DMSO-d$_6$) δ (ppm) 178.28, 163.66, 163.50, 163.45, 163.00, 161.00, 159.43, 140.17, 133.22, 131.14, 128.96, 128.69, 128.62, 128.46, 126.51, 123.06, 121.96, 118.41, 114.33, 107.54, 70.45, 61.11, 60.98, 41.06, 30.96, 30.88, 23.57, 23.51, 22.88, 20.92, 11.40. ^{195}Pt NMR (86 MHz, DMSO-d$_6$) δ (ppm) 1625.02. **HRMS** [M+H]$^+$: expected 860.2101; observed 860.2097.

[Pt(Cl)$_2$(NH$_3$)$_2$(4-(((1,3-dioxo-2-propyl-2,3-dihydro-1H-benzo[de]isoquinolin-6-yl)oxy)methyl)phenyl)carbamic acid)(OAc)] (CisBlue)

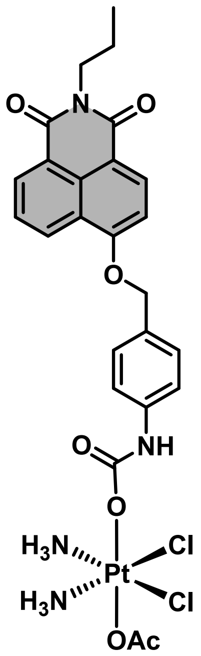

Yellow solid (27 mg, 22 % yield). ^{1}H NMR (400 MHz, DMSO-d$_6$) δ (ppm): 9.16 (br s, 1H), 8.57 – 8.43 (m, 3H), 7.81 (t, *J* = 7.8 Hz, 1H), 7.52 (d, *J* = 8.1 Hz, 2H), 7.42 (dd, *J* = 14.0, 8.4 Hz, 3H), 6.65 (br s, 6H), 5.36 (s, 2H), 4.00 (t, *J* = 7.5 Hz, 2H), 1.93 (s, 3H), 1.64 (q, *J* = 7.4 Hz, 2H), 0.91 (t, *J* = 7.4 Hz, 3H). ^{13}C NMR (151 MHz, DMSO-d$_6$) δ (ppm) 178.40, 163.66, 163.01, 159.49, 140.97, 133.22, 131.12, 128.67, 128.61, 128.57, 128.47, 128.20, 128.17, 126.49, 123.04, 121.92, 118.09, 114.27, 107.52, 70.62, 41.07, 20.94, 11.42. ^{195}Pt NMR (86 MHz, DMSO-d$_6$): δ (ppm) 1253. **HRMS** [M+H]$^+$: expected 762.1056; observed 762.1034.

3. Additional Analyses

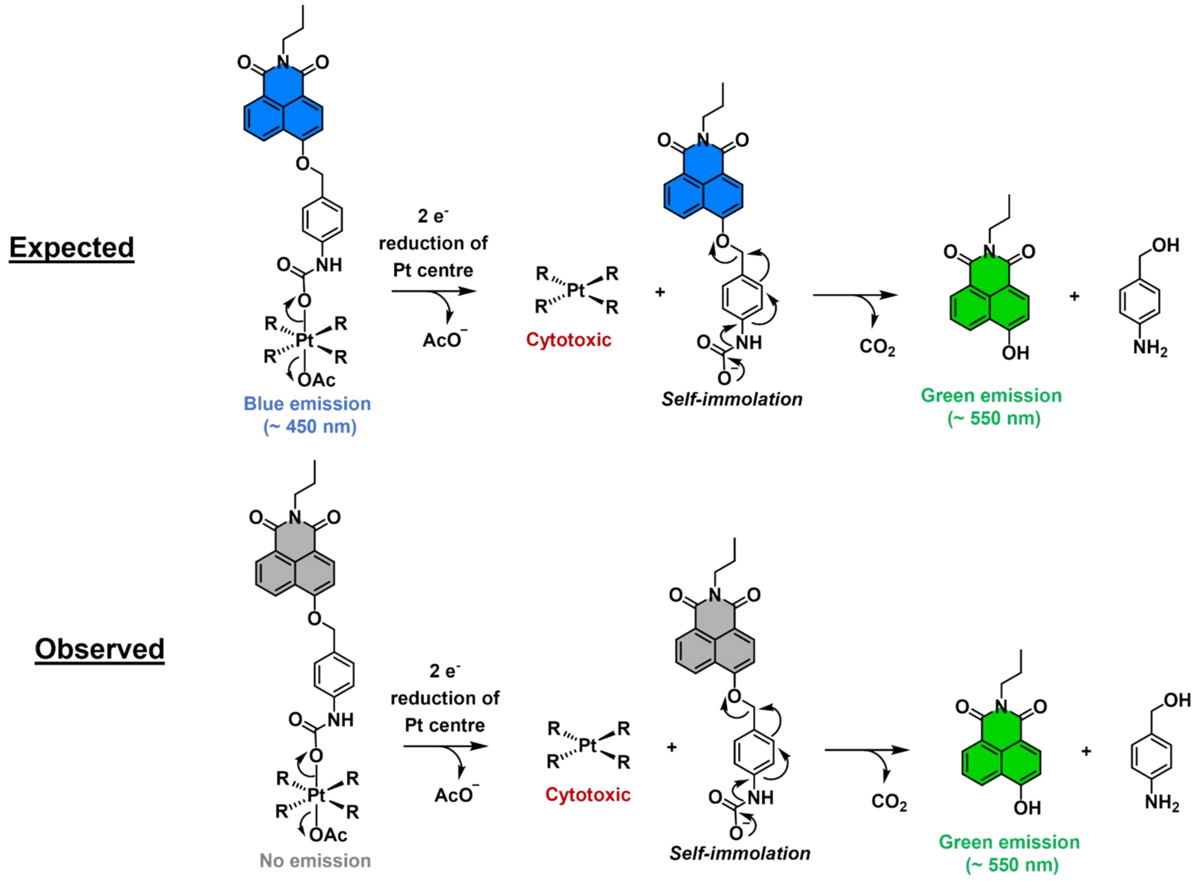

Scheme S4. Top: Schematic of the proposed ratiometric probes' showing blue emission of initial Pt(IV) complex to green emission due to release of fluorescent Nap-OH fluorophore caused by reduction of Pt centre and release of the self-immolative linker. Bottom: Schematic for the observed emission profiles, where minimal fluorescence emission was observed for each Pt(IV) complex and the green emission of Nap-OH fluorophore.

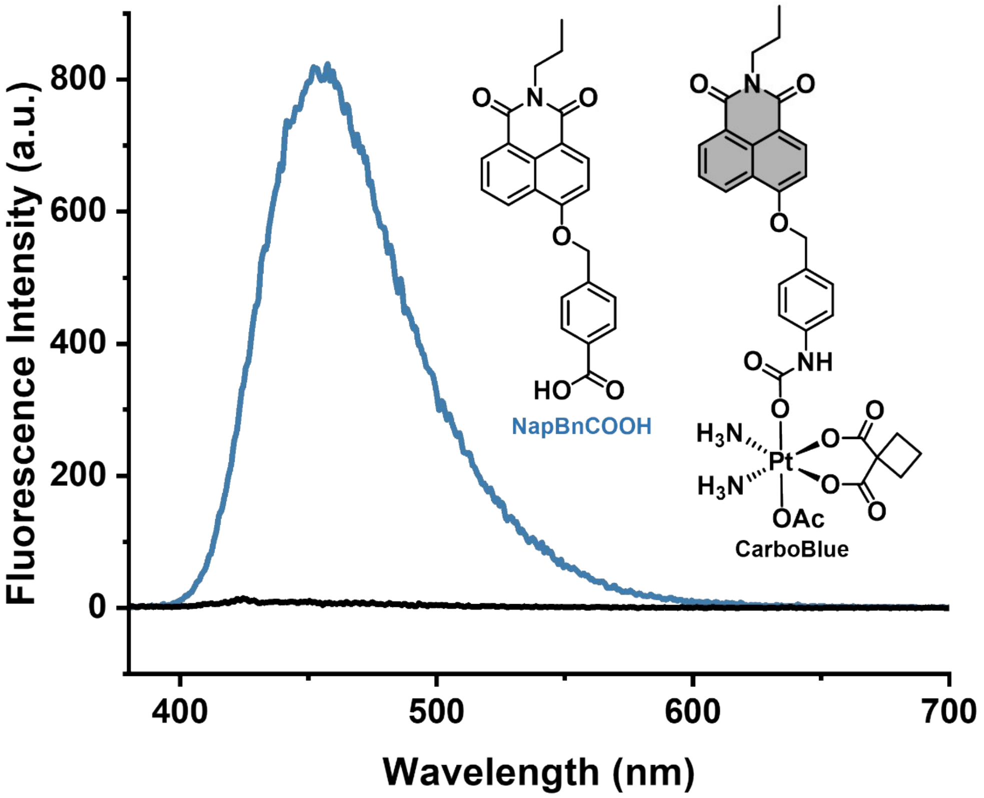

Figure S1. Fluorescence emission spectra of **NapBnCOOH** and **CarboBlue** (both 0.42 μM) in PBS (pH = 7.4, 0.01 M) at 25 °C. λ_{exc} = 370 nm; slit widths: 5 nm and 2.5 nm.

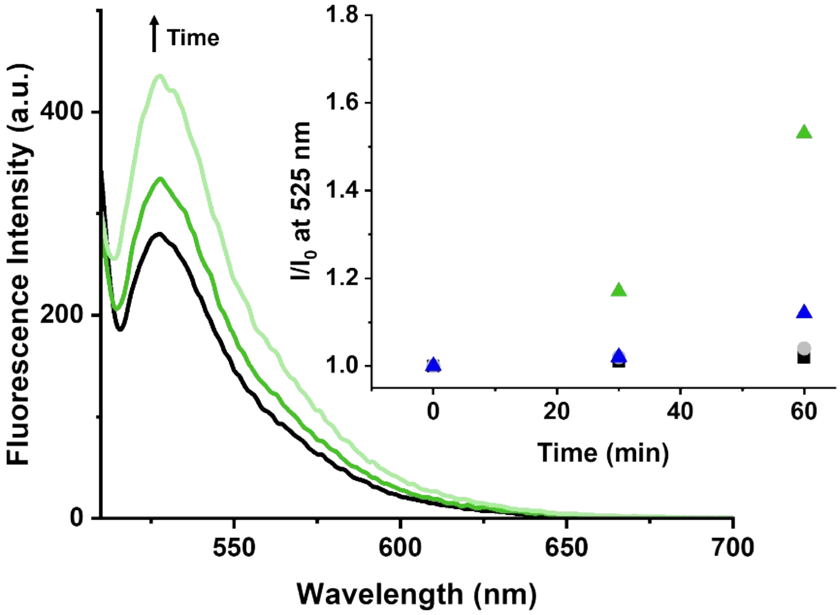

Figure S2. Changes in fluorescence emission intensity of Singlet Oxygen Sensor Green (SOSG, 1 µM) incubated with **CarboBlue** (5 µM) and irradiated with blue light (spectral width: 400–500 nm, 4 W) at different time points (0, 30 and 60 mins). Inset: Fold change in fluorescence emission intensity at 525 nm of SOSG and **CarboBlue** with (green triangles) and without (grey circles) blue light irradiation; and SOSG incubated in the absence (black squares) and presence (blue triangles) of light irradiation. All solutions prepared in PBS buffer (pH = 7.4). λ_{ex} = 504 nm; λ_{em} = 525 nm; slit widths 10 nm and 2.5 nm.

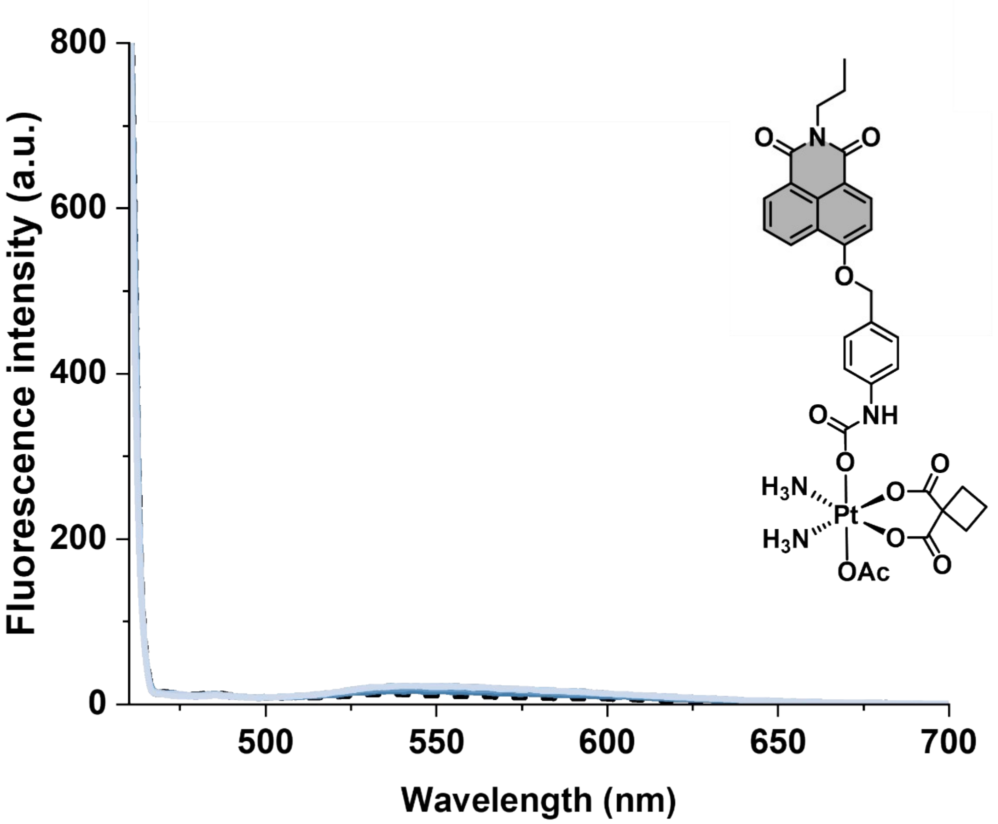

Figure S3. Fluorescence emission spectra of **CarboBlue** (5 µM) incubated with NaAsc (10 mM) and measured at different time points (0 ,10, 20, 30, 40, 50, and 60 min) in PBS (pH = 7.4) at 25 °C. λ_{ex} = 450 nm; slit widths 10 nm and 5 nm.

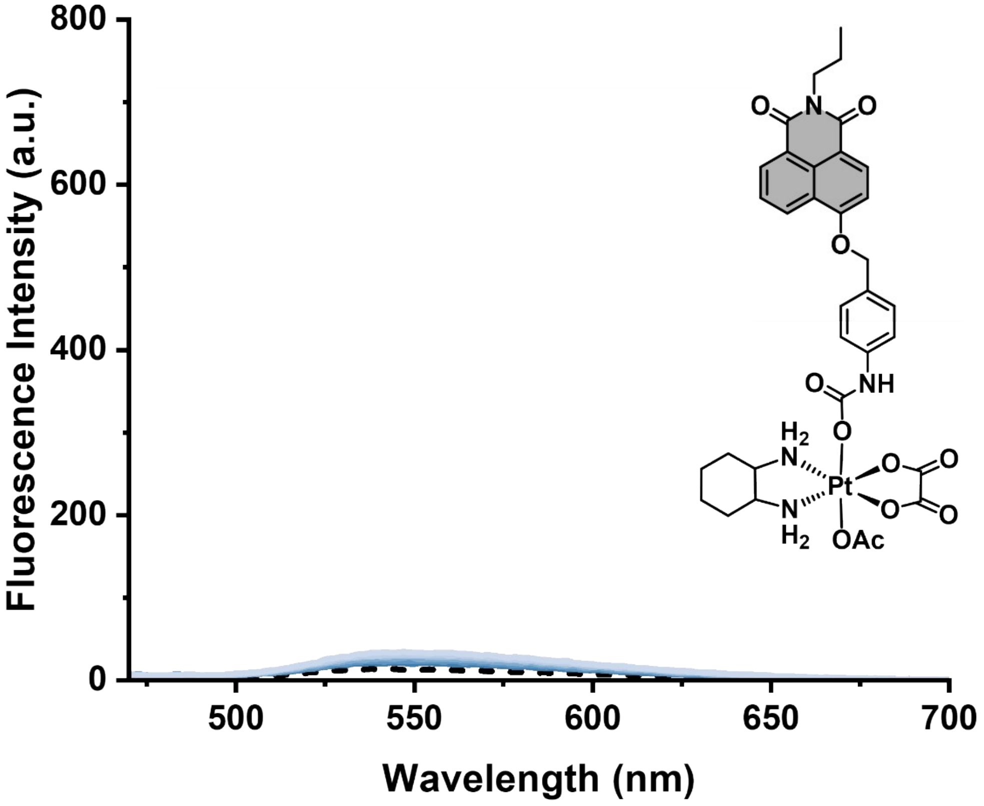

Figure S4. Fluorescence emission spectra of **OxaliBlue** (5 µM) incubated with NaAsc (10 mM) and measured at different time points (0 ,10, 20, 30, 40, 50, and 60 min) in PBS (pH = 7.4) at 25 °C. λ_{ex} = 450 nm; slit widths 10 nm and 5 nm.

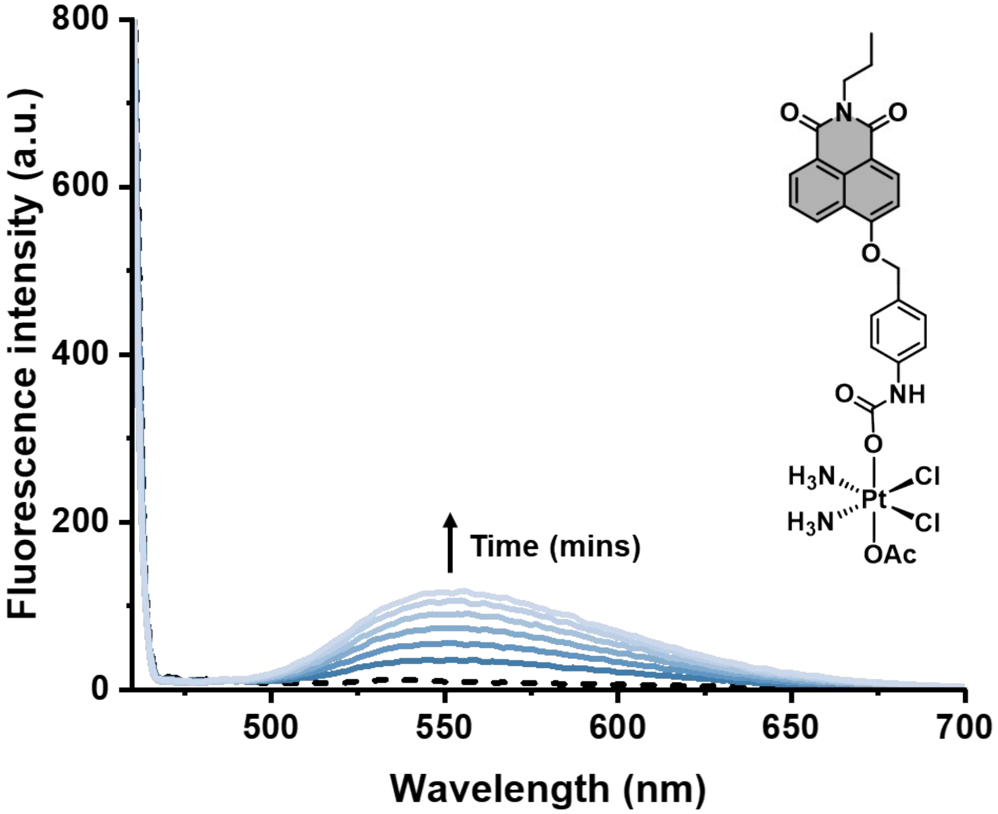

Figure S5. Fluorescence emission spectra of **CisBlue** (5 µM) incubated with NaAsc (10 mM) and measured at different time points (0 ,10, 20, 30, 40, 50, and 60 min) in PBS (pH = 7.4) at 25 °C. λ_{ex} = 450 nm; slit widths 10 nm and 5 nm.

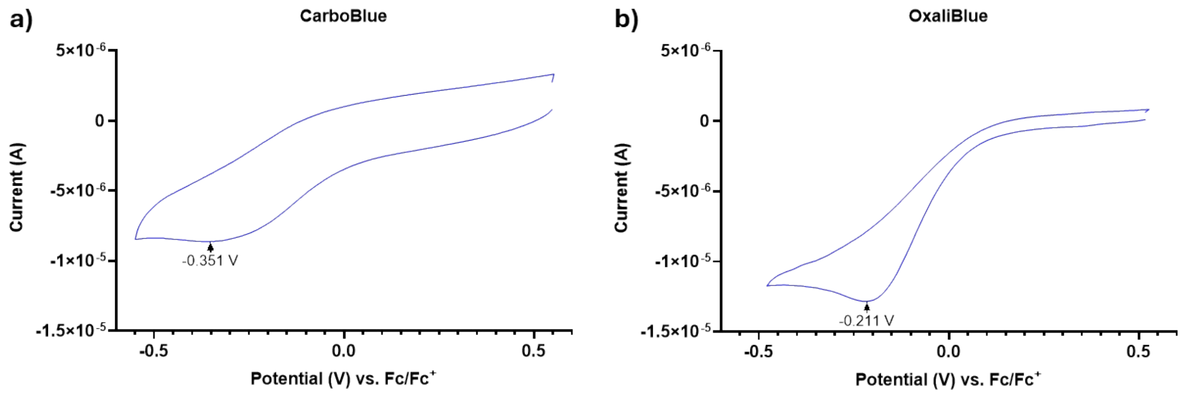

Figure S6. Cyclic voltammogram of a 3 mm glassy carbon electrode immersed in 1 mM solution of (a) **CarboBlue** and (b) **OxaliBlue** in DMF (+ Tetrabutylammonium hexafluorophosphate (200mM)) at a scan rate of 0.1 V/s.

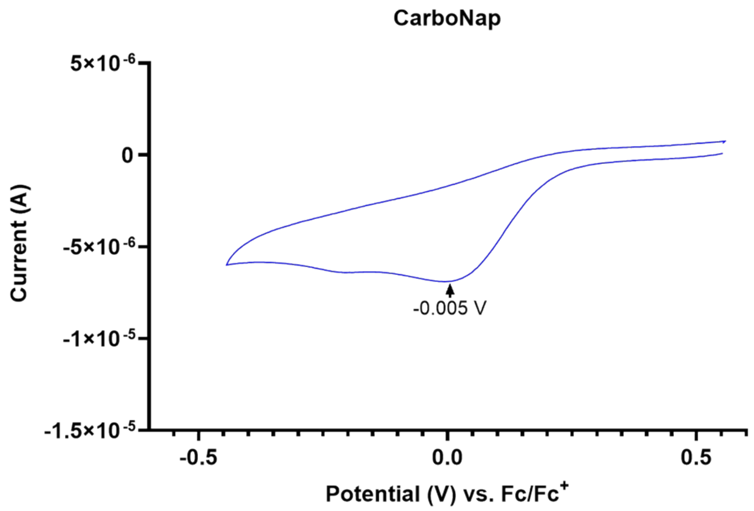

Figure S7. Cyclic voltammogram of a 3 mm glassy carbon electrode immersed in 1 mM solution of **CarboNap** in DMF (+Tetrabutylammonium hexafluorophosphate (200mM)) at a scan rate of 0.1 V/s.

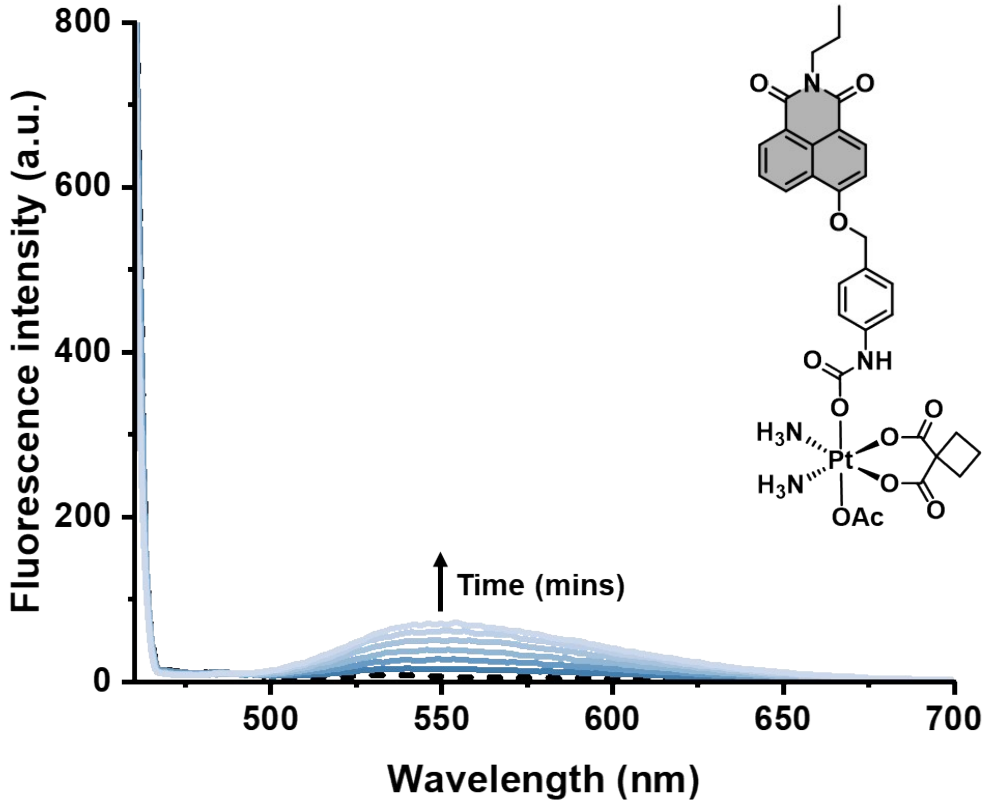

Figure S8. Fluorescence emission spectra of **CarboBlue** (5 μM) irradiated with blue light (spectral width: 400 - 500 nm, 4 W) and measured at 10-minute interval (0 ,10, 20, 30, 40, 50, and 60 mins) in PBS (pH = 7.4) at 25 °C. λ_{ex} = 450 nm; slit widths 10 nm and 5 nm.

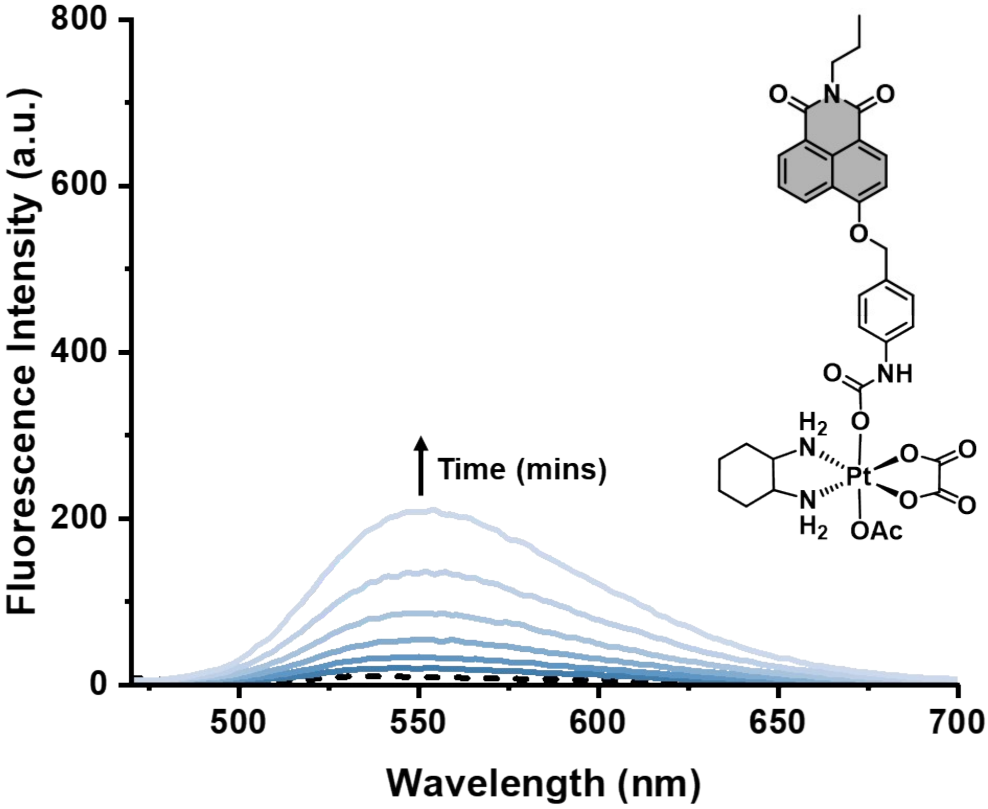

Figure S9. Fluorescence emission spectra of **OxaliBlue** (5 µM) irradiated with blue light (spectral width: 400 - 500 nm, 4 W) and measured at 10-minute intervals (0, 10, 20, 30, 40, 50, and 60 mins) in PBS (pH = 7.4) at 25 °C. λ_{ex} = 450 nm; slit widths 10 nm and 5 nm.

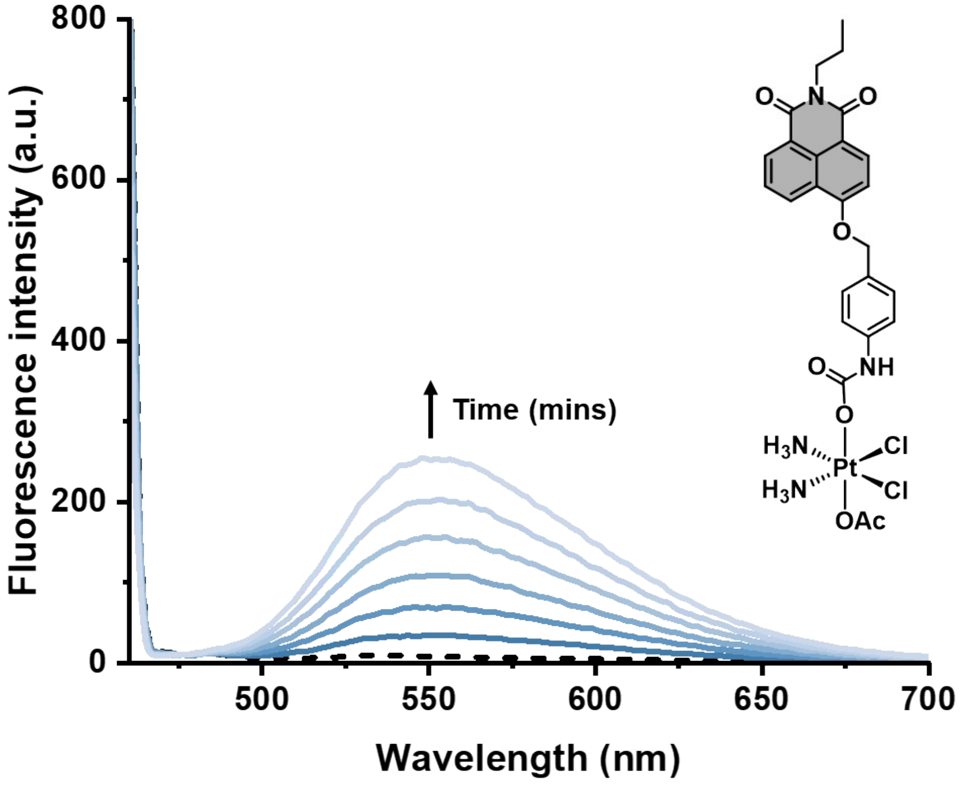

Figure S10. Fluorescence emission spectra of **CisBlue** (5 μM) irradiated with blue light (spectral width: 400 - 500 nm, 4 W) and measured at 10-minute intervals (0, 10, 20, 30, 40, 50, and 60 mins) in PBS (pH = 7.4) at 25 °C. λ_{ex} = 450 nm; slit widths 10 nm and 5 nm.

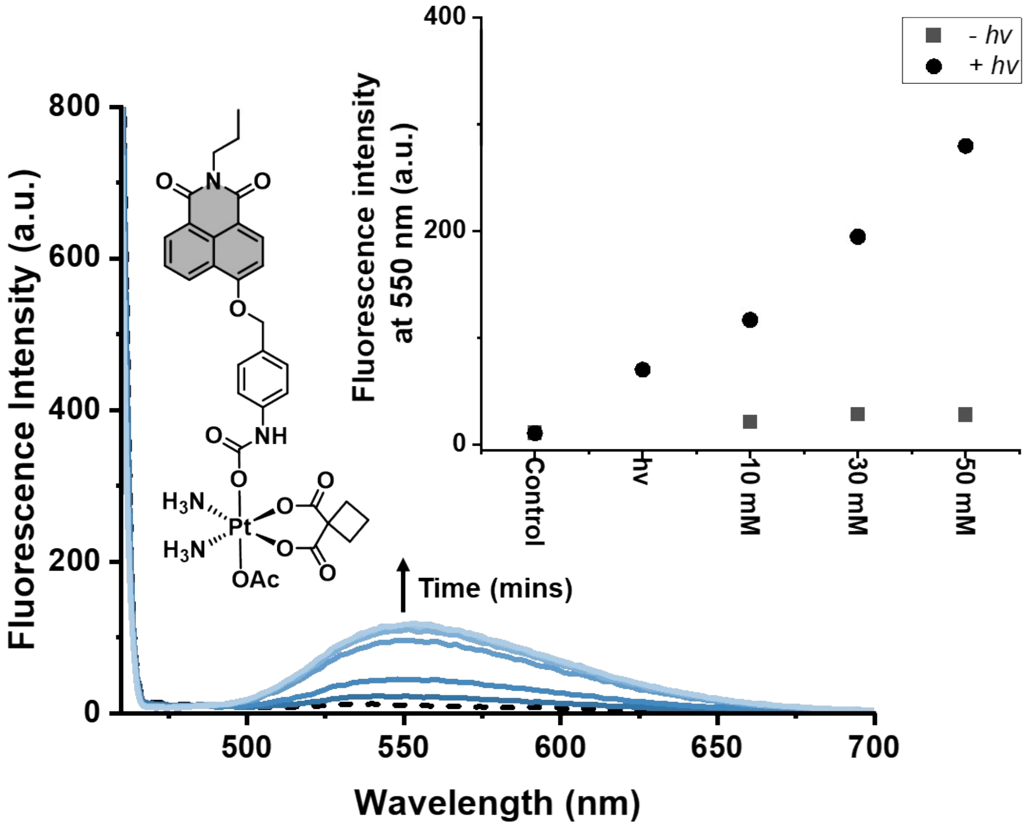

Figure S11. Fluorescence emission spectra of **CarboBlue** (5 μM) irradiated with blue light (spectral width: 400 - 500 nm, 4 W) and NaAsc (10 mM) at different time points (0, 10, 20, 30, 40, 50, and 60 mins) in PBS (pH = 7.4, 0.01 M) at 25 °C. λ_{ex} = 450 nm; slit widths 10 nm and 5 nm. Inset: Fluorescence emission intensity of **CarboBlue** (5 μM) incubated with different concentrations of NaAsc (10, 30, and 50 mM) +/- *hv* (1 h).

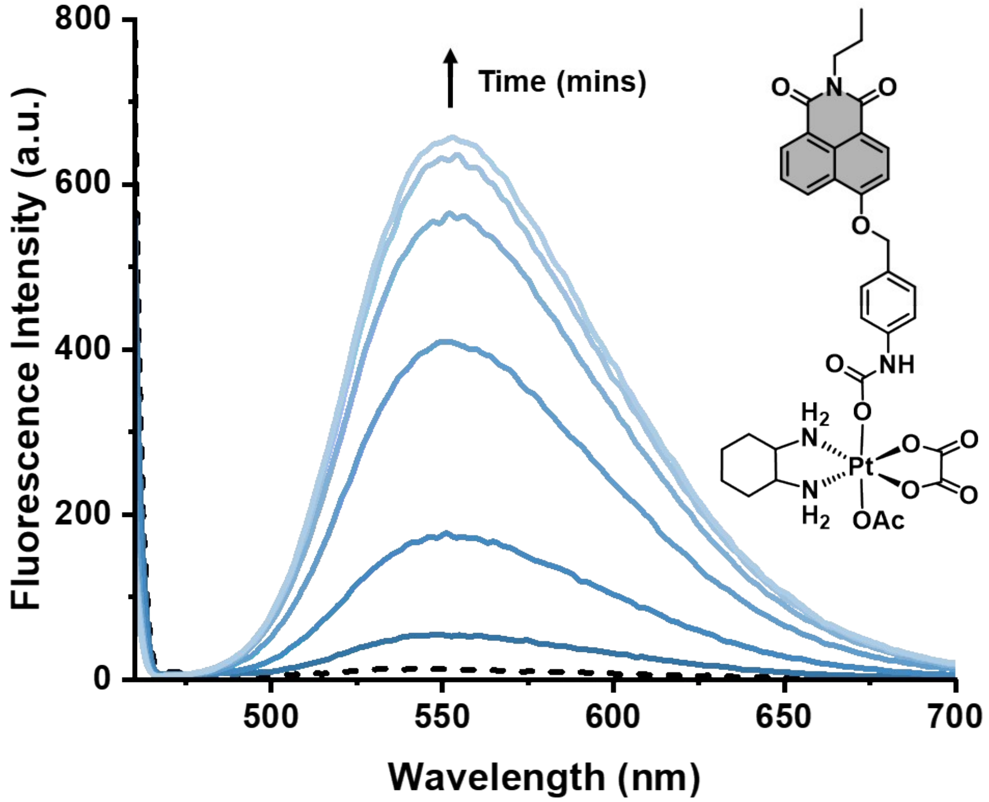

Figure S12. Fluorescence emission spectra of **OxaliBlue** (5 μM) irradiated with blue light (spectral width: 400–500 nm, 4 W) in the presence of NaAsc (10 mM) at different time points (0, 10, 20, 30, 40, 50, and 60 mins) in PBS (pH = 7.4) at 25 °C. λ_{ex} = 450 nm; slit widths 10 nm and 5 nm.

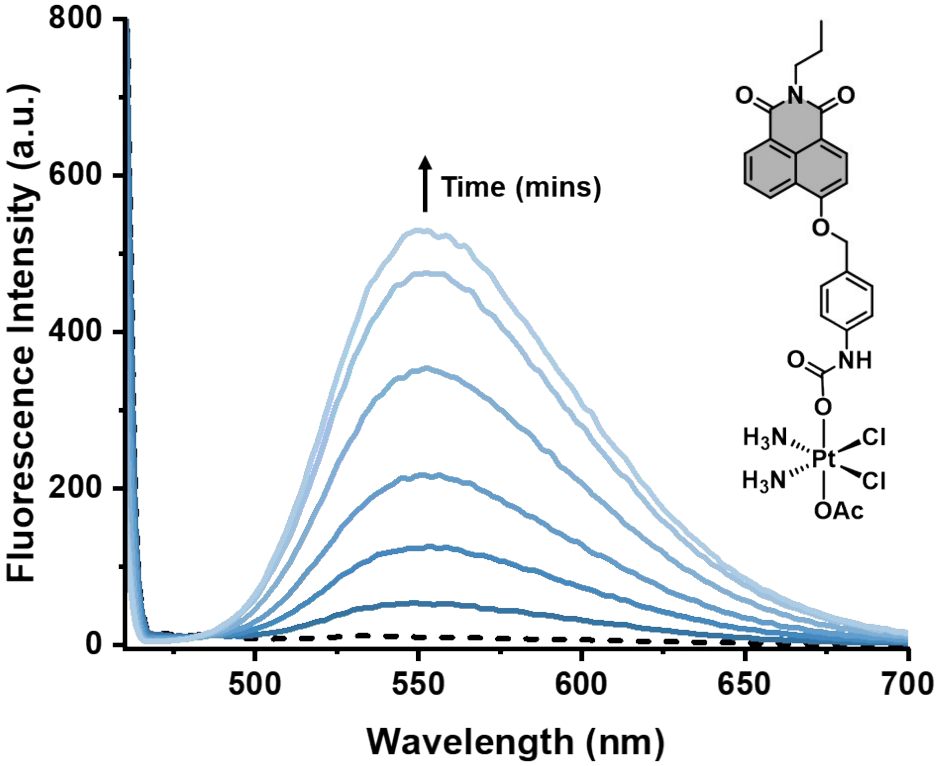

Figure S13. Fluorescence emission spectra of **CisBlue** (5 µM) irradiated with blue light (spectral width: 400–500 nm, 4 W) in the presence of NaAsc (10 mM) at different time points (0, 10, 20, 30, 40, 50, and 60 mins) in PBS (pH = 7.4) at 25 °C. λ_{ex} = 450 nm; slit widths 10 nm and 5 nm.

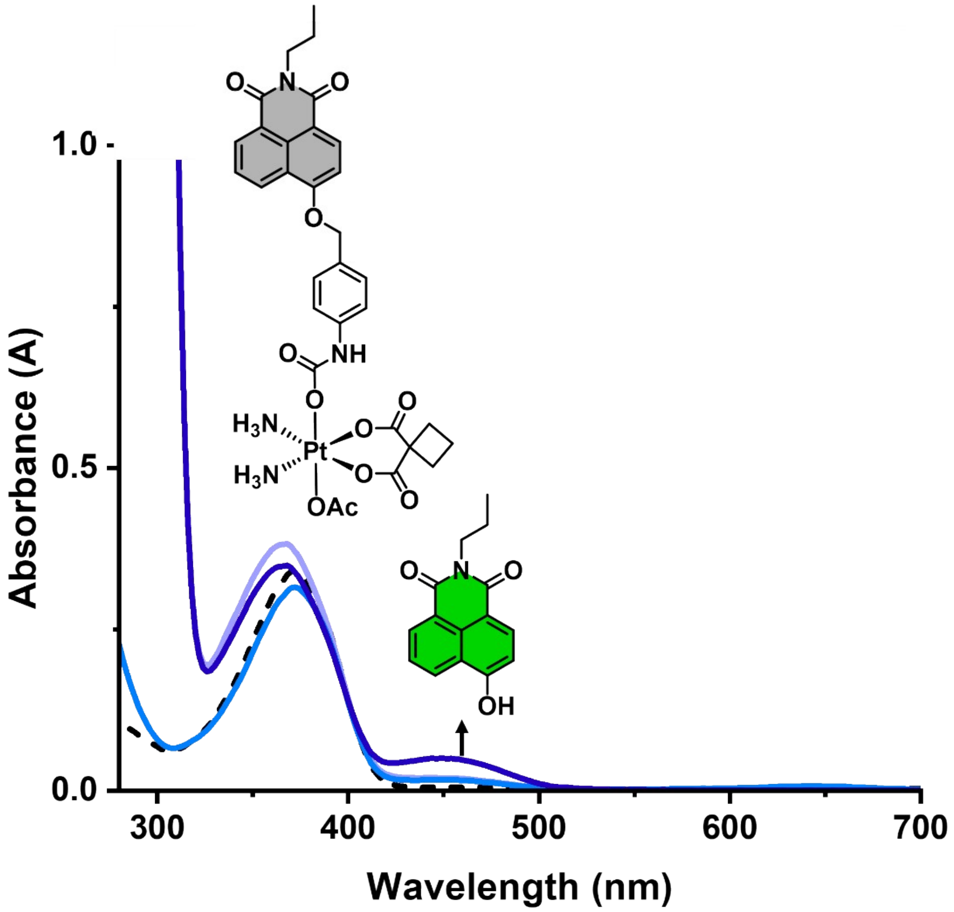

Figure S14. Absorbance spectra of **CarboBlue** (25 µM, dashed lines, λ_{max} = ~ 370 nm) in H_2O/MeCN (70/ 30 %) solution after 1 h incubation with: NaAsc (40 mM, shaded purple); blue light (blue); and both NaAsc (40 mM) and blue light (purple) showing release of **Nap-OH** (λ_{max} = ~ 450 nm). Note - greater concentrations of NaAsc and **CarboBlue** were used in this study so we could observe the release of Nap-OH.

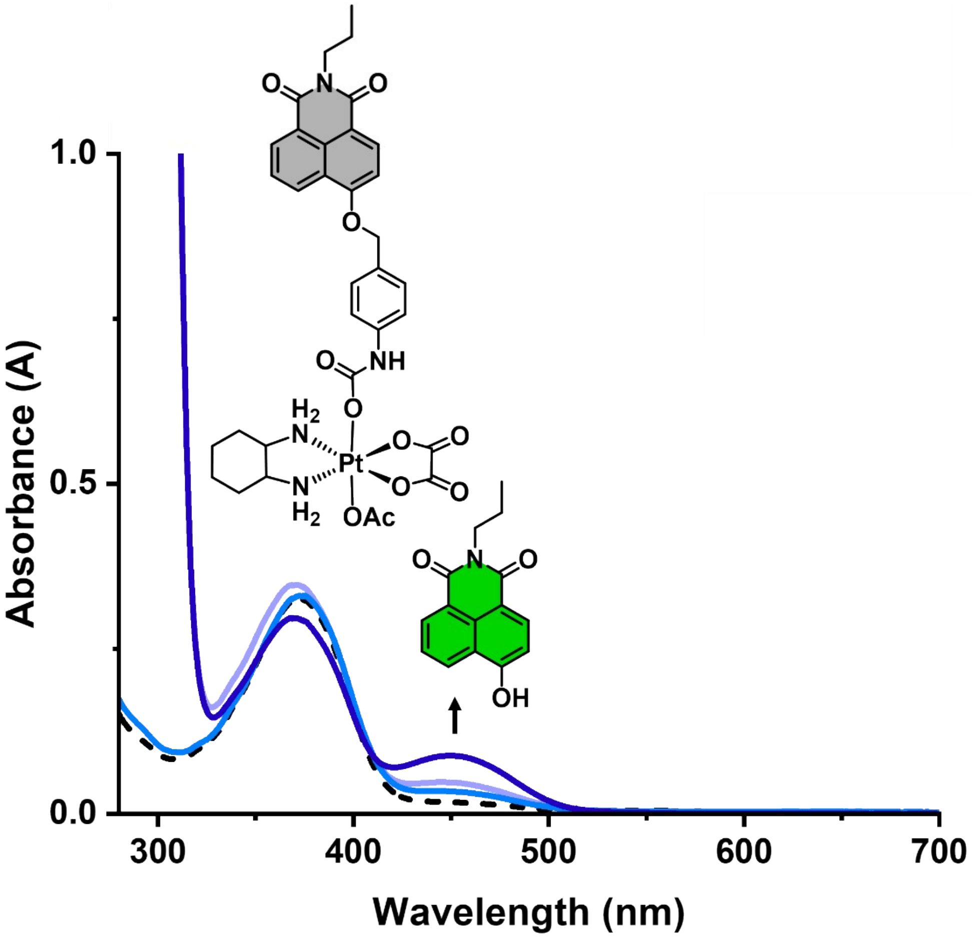

Figure S15. Absorbance spectra of **OxaliBlue** (25 µM, dashed lines, λ_{max} = ~ 370 nm) in a H_2O/MeCN (70/30 %) solution after 1 h incubation with: NaAsc (40 mM, shaded purple); blue light (blue); and both NaAsc (40 mM) and blue light (purple) showing release of **Nap-OH** (λ_{max} = ~ 450 nm). Note - greater concentrations of NaAsc and **OxaliBlue** were used in this study so we could observe the release of Nap-OH.

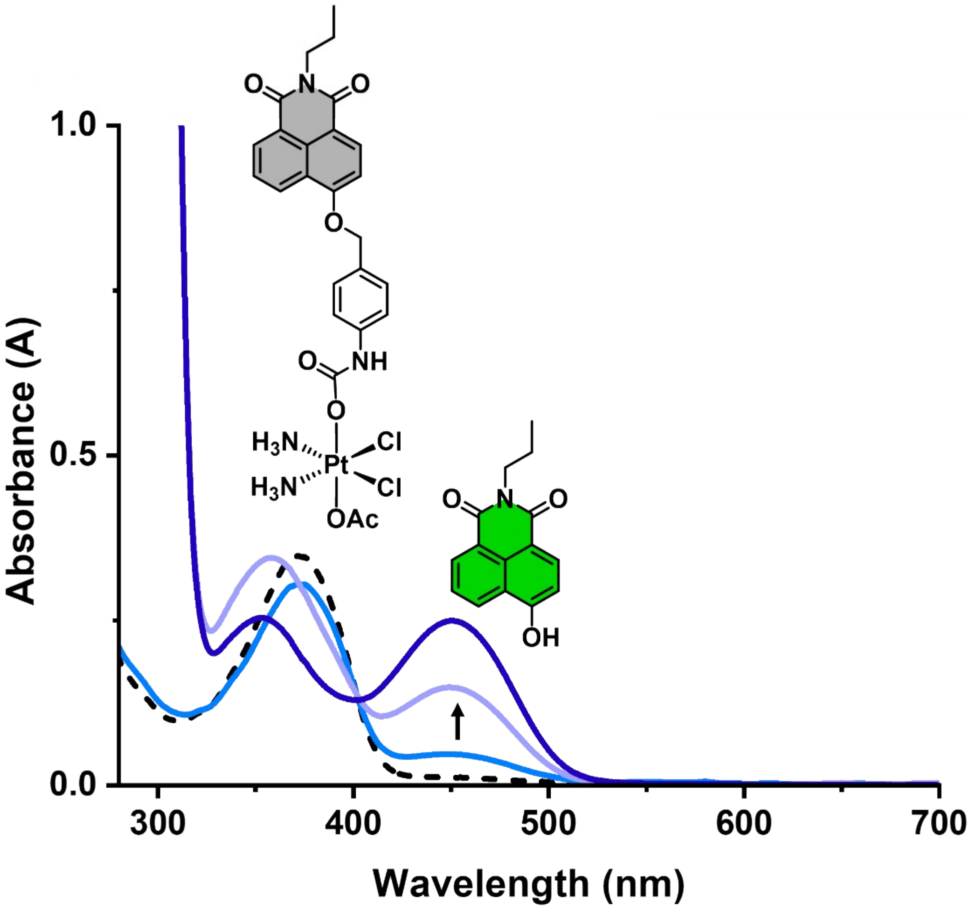

Figure S16. Absorbance spectra **CisBlue** (25 µM, dashed lines, λ_{max} = ~ 370 nm) in a H_2O/MeCN (70/30 %) solution after 1 h incubation with: NaAsc (40 mM, **shaded purple**); blue light (**blue**); and both NaAsc (40 mM) and blue light (**purple**) showing release of **Nap-OH** (λ_{max} = ~ 450 nm). Note - greater concentrations of NaAsc and **CisBlue** were used in this study so we could observe the release of Nap-OH.

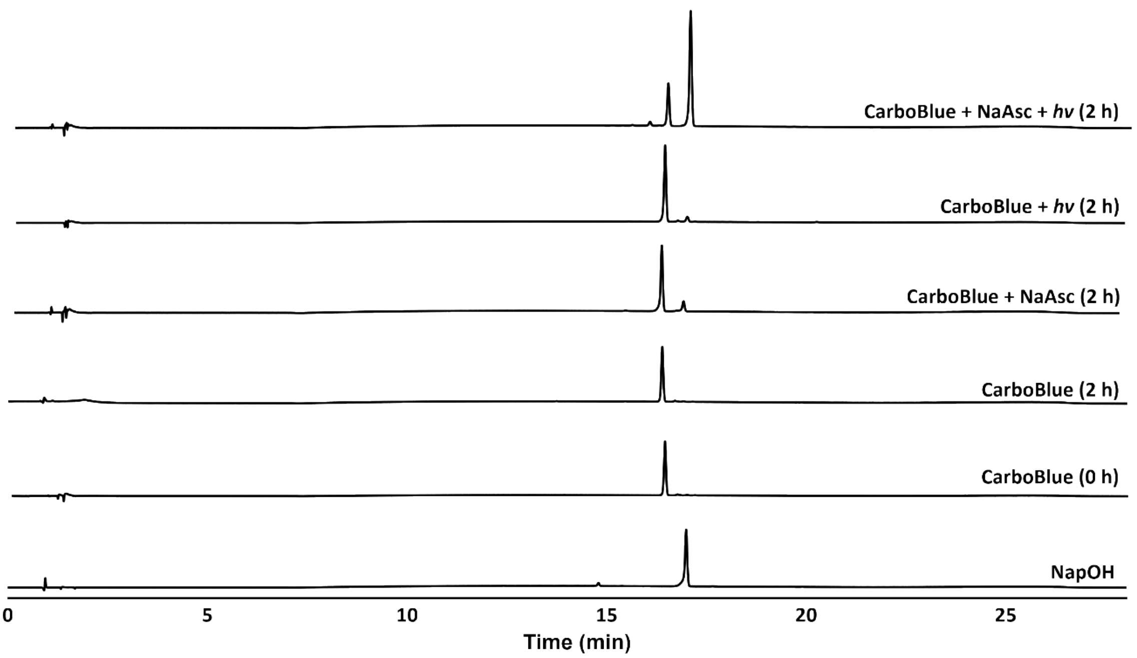

Figure S17. HPLC chromatograms (absorbance 400 nm) of: **Nap-OH** and **CarboBlue** (0 and 2 h), and **CarboBlue** incubated with NaAsc (50 mM, 2 h); irradiated with blue light (2 h); and incubated with NaAsc (50 mM) and blue light irradiation (2 h). All working solutions were prepared in a H_2O/MeCN (70/30 %) solution; [**CarboBlue**] = 50 µM, [**NapOH**] = 50 µM and performed at 25 °C.

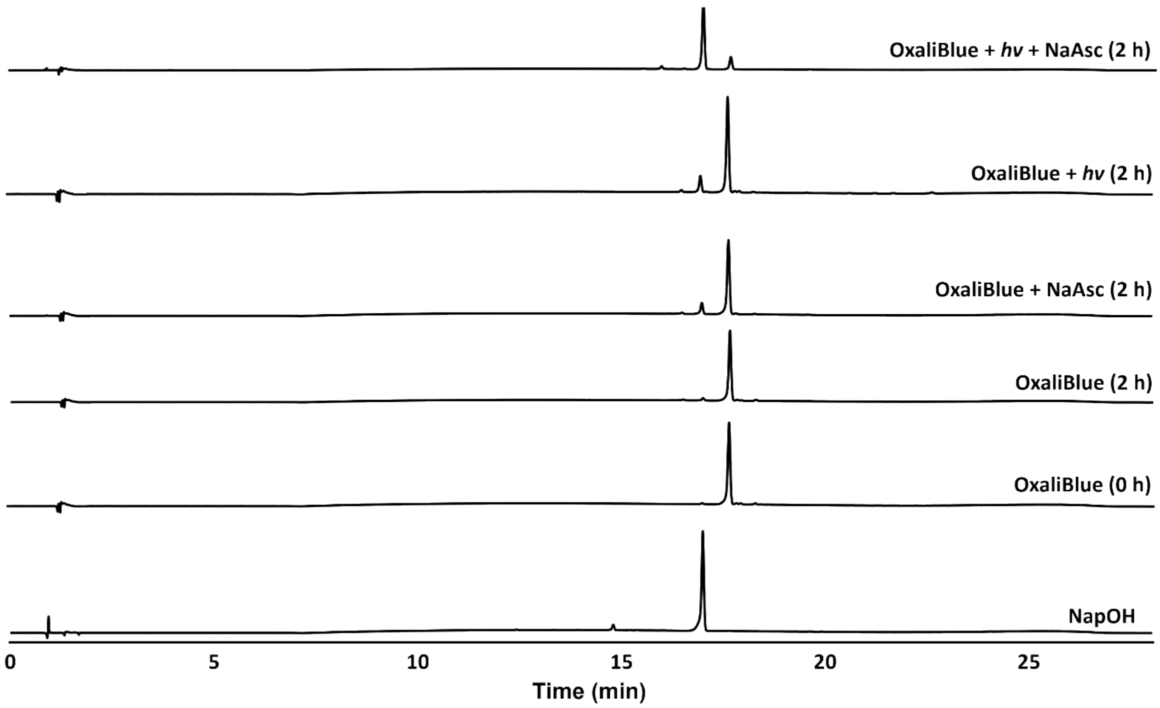

Figure S18. HPLC chromatograms (absorbance 400 nm) of: **NapOH** and **OxaliBlue** (0 and 2 h), and **OxaliBlue** incubated with NaAsc (50 mM, 2 h); irradiated with blue light (2 h); and incubated with NaAsc (50 mM) and blue light irradiation (2 h). All working solutions were prepared in a H_2O/MeCN (70/30 %) solution; [**OxaliBlue**] = 50 µM, [**Nap-OH**] = 50 µM and performed at 25 °C.

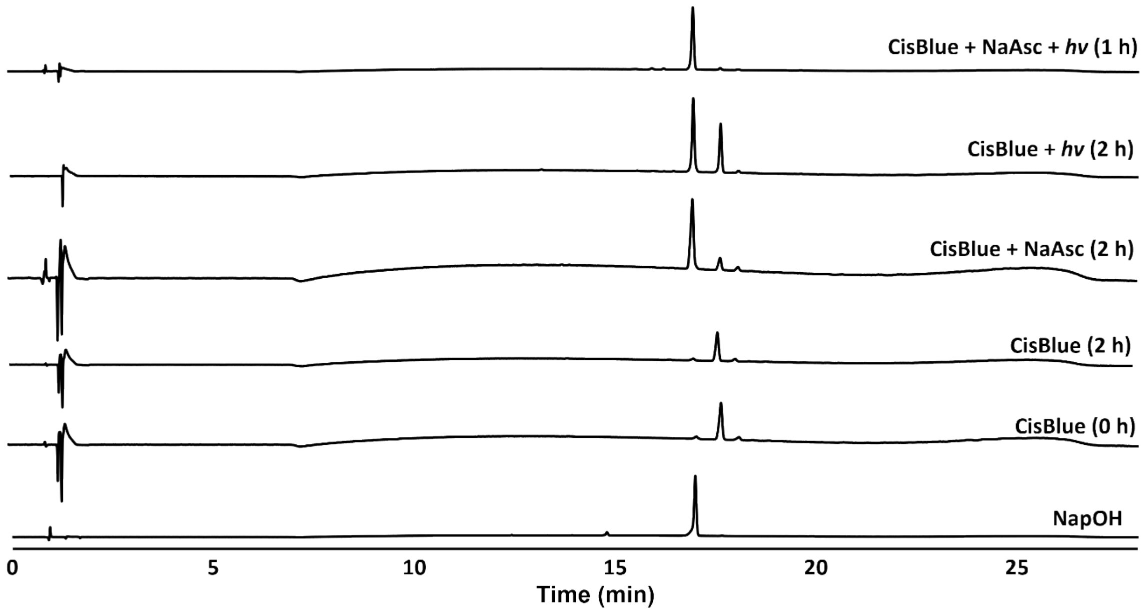

Figure S19. HPLC chromatograms (absorbance 400 nm) of: **Nap-OH** and **CisBlue** (0 and 2 h) and **CisBlue** incubated with NaAsc (50 mM, 2 h); irradiated with blue light (2 h); and incubated with NaAsc (50 mM) and blue light irradiation (1 h). All working solutions were prepared in a H_2O/MeCN (70/30 %) solution; [**CisBlue**] = 50 µM, [**Nap-OH**] = 50 µM and performed at 25 °C.

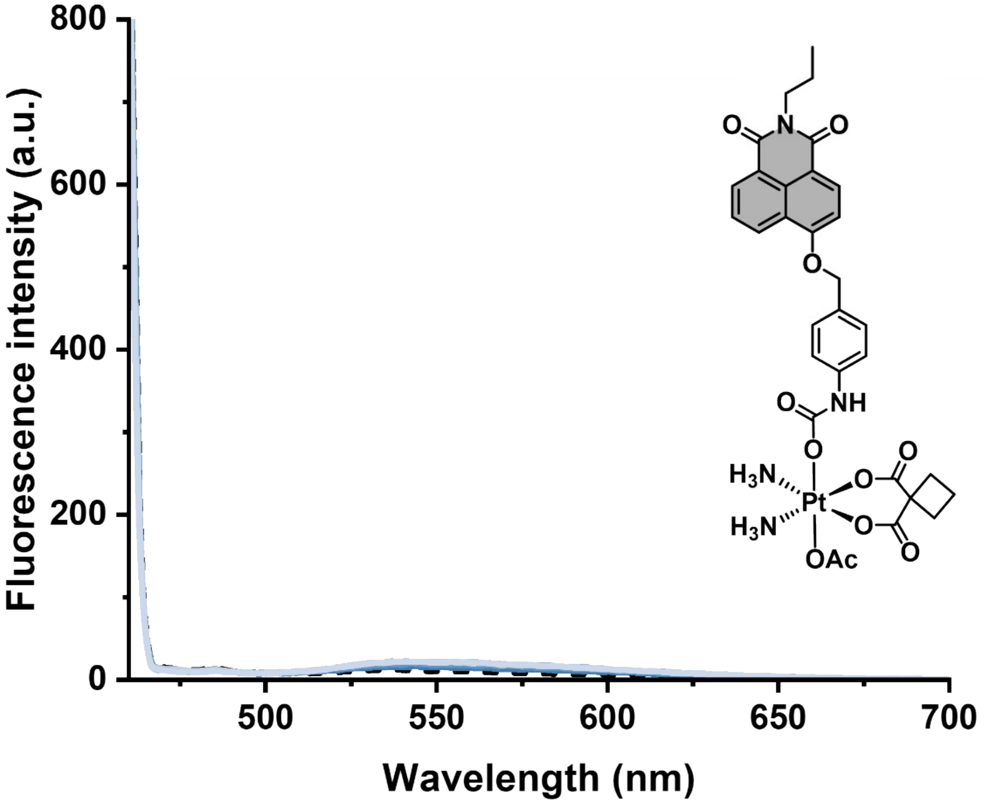

Figure S20. Fluorescence emission spectra of **CarboBlue** (5 µM) at different time points (0, 10, 20, 30, 40, 50, and 60 mins) in PBS (pH 7.4) at 25 °C. λ_{ex}= 450 nm; slit widths 10 nm and 5 nm.

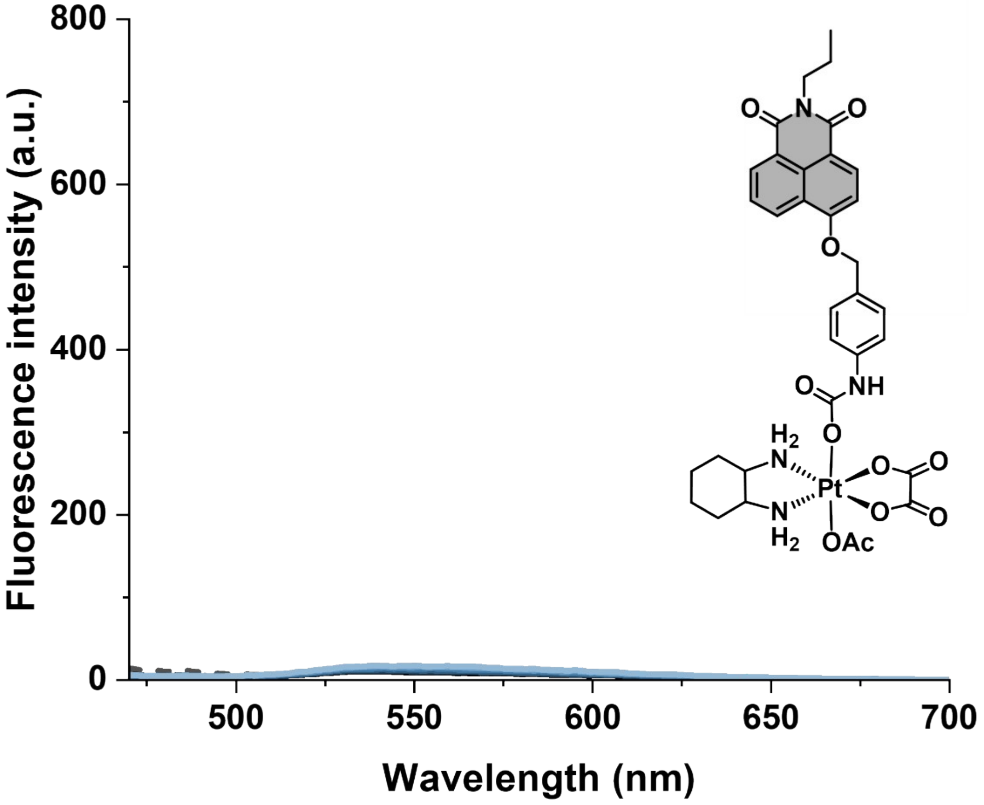

Figure S21. Fluorescence emission spectra of **OxaliBlue** (5 μM) at different time points (0, 10, 20, 30, 40, 50, and 60 mins) in PBS (pH 7.4) at 25 °C. λ_{ex} = 450 nm; slit widths 10 nm and 5 nm.

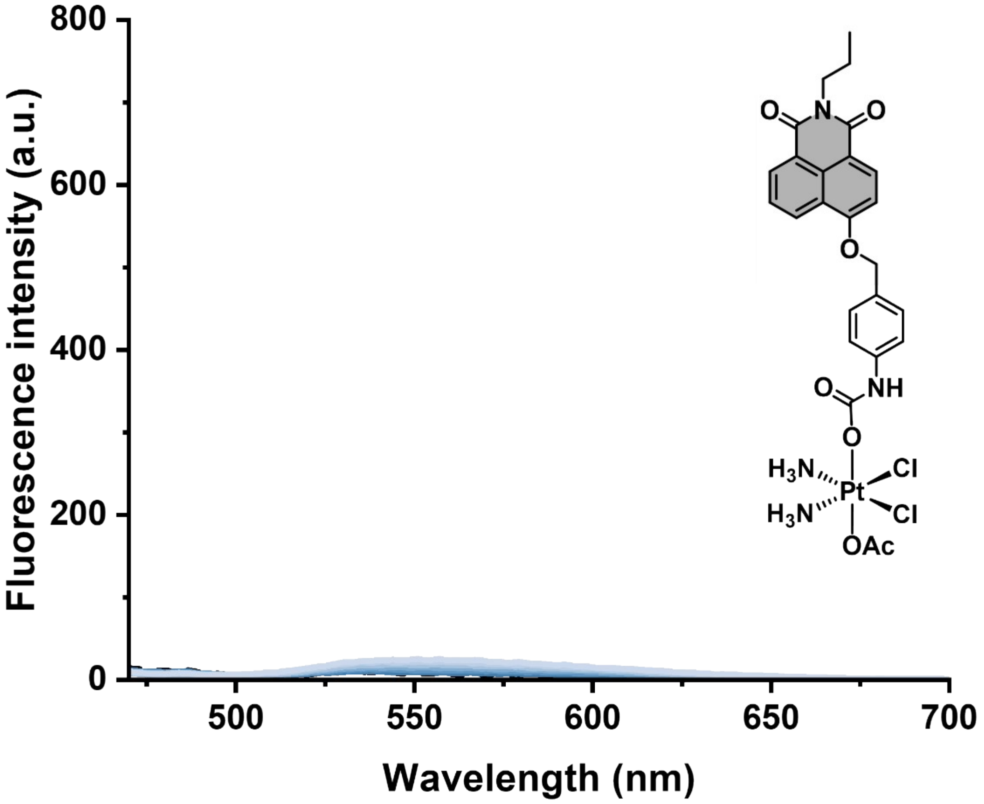

Figure S22. Fluorescence emission spectra of **CisBlue** (5 μM) at different time points (0, 10, 20, 30, 40, 50, and 60 mins) in PBS (pH 7.4) at 25 °C. λ_{ex} = 450 nm; slit widths 10 nm and 5 nm.

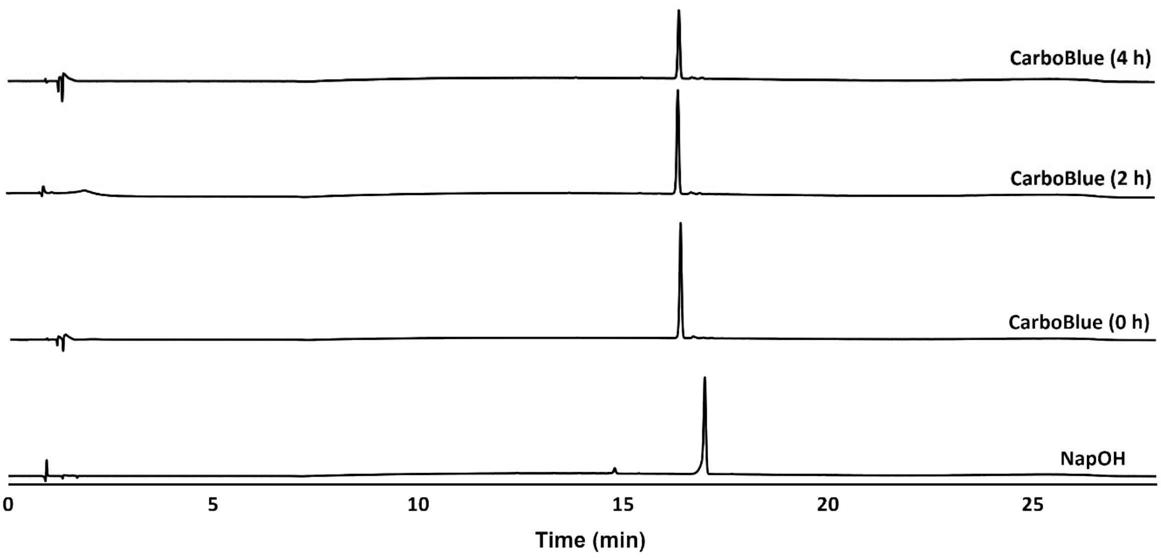

Figure S23. HPLC chromatograms (absorbance 400 nm) of **Nap-OH** (50 µM, bottom) and **CarboBlue** (50 µM) at different time points (0, 2 and 4 h) in a H_2O/MeCN (70/30 %) solution at 25 °C.

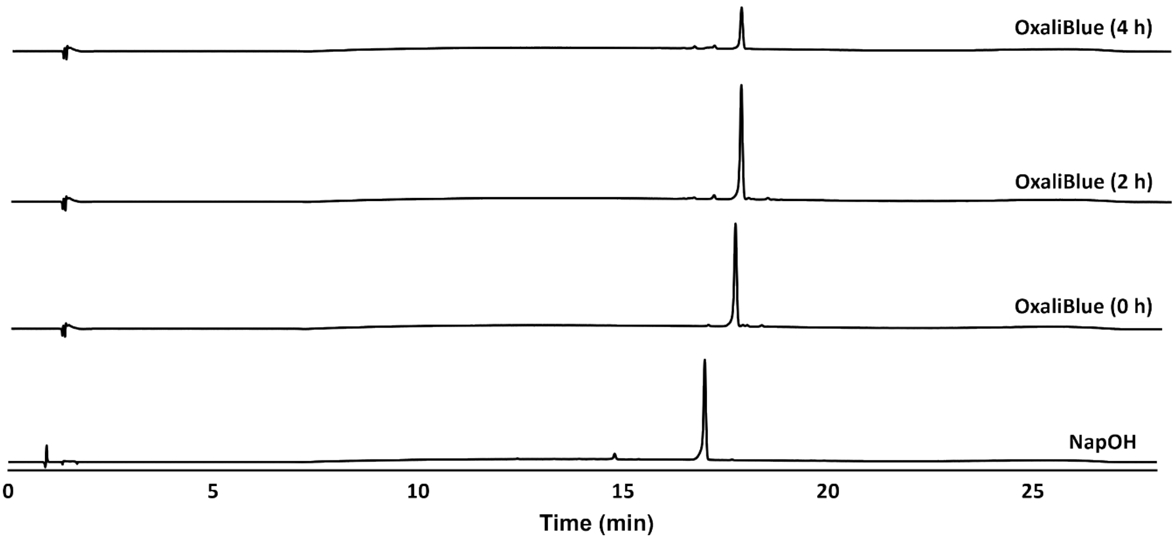

Figure S24. HPLC chromatograms (absorbance 400 nm) of **Nap-OH** (50 µM, bottom) and **OxaliBlue** (50 µM) at different time points (0, 2 and 4 h) in a H_2O/MeCN (70/30 %) solution at 25 °C.

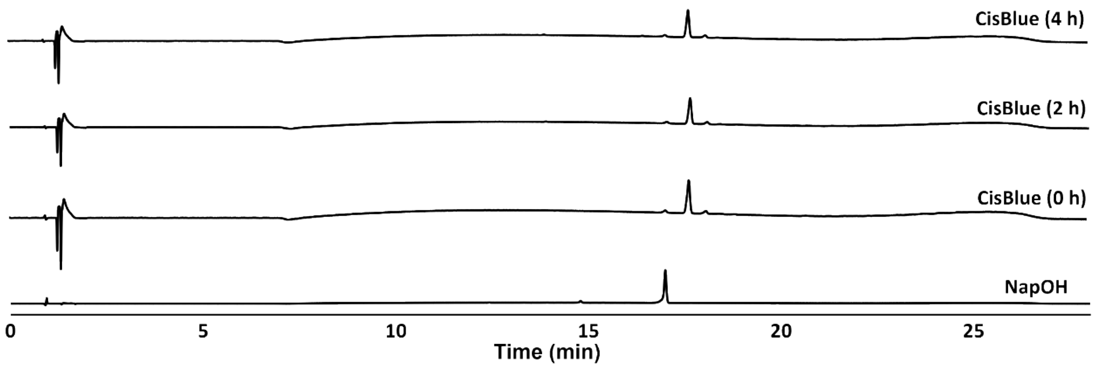

Figure S25. HPLC chromatograms (absorbance 400 nm) of **Nap-OH** (50 µM, bottom) and **CisBlue** (50 µM) at (0, 2 and 4 h) in H_2O/MeCN (70/30 %) at 25 °C.

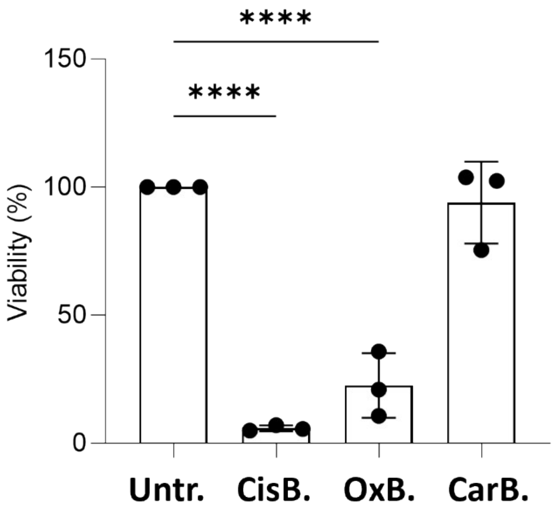

Figure S26. HCT116 cells were treated with **CarboBlue**, **CisBlue** and **OxaliBlue** (10 µM) for 64 hours in normal conditions followed by an MTT assay. Untr. = untreated cells. Error bars represent SD. **** = $p < 0.0001$. n = 3.

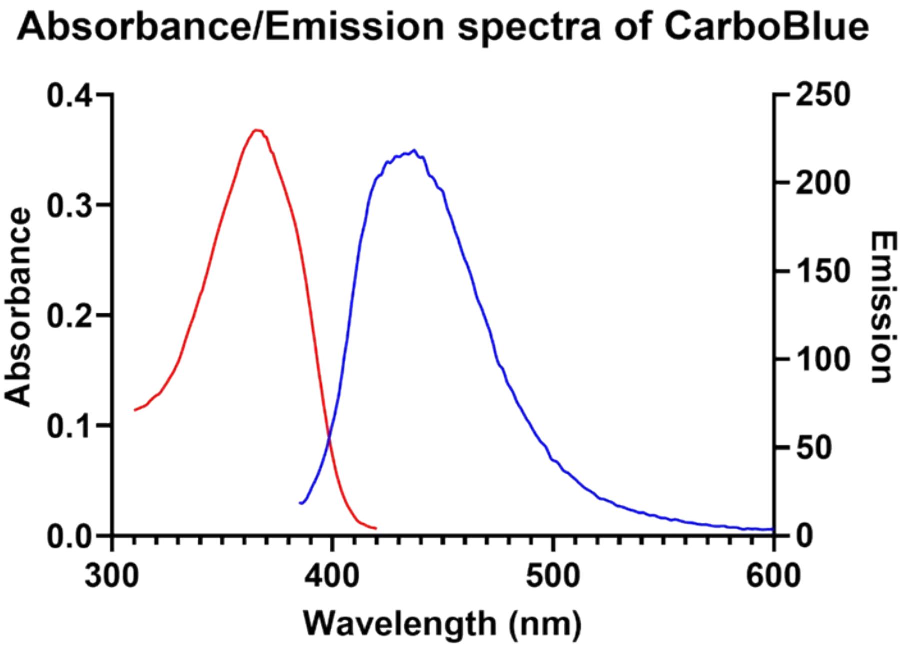

Figure S27. Absorbance and Emission spectra of **CarboBlue** in DMF ([**PtBlue**]$_{em}$ = 5 µM; [**PtBlue**]$_{Abs}$ = 30 µM); λ_{ex} = 450 nm; slit widths 10 nm and 5 nm. $\lambda_{intersection}$ = 398 nm.

The calculation of the excited state reduction potentials was carried out following the Rehm Weller equation.[5]

$$E^{*red} = E^{red} + E_{00}$$

$$E_{00} = \frac{hc}{\lambda}$$

$$E_{00} = \frac{1239.8}{398\ nm} = 3.12\ eV$$

Calculation for excited state reduction potential, E_{red}^{*} for **CarboBlue:**

$$E^{*red} = E^{red} + E_{00}$$

$$E^{*red} =- 0.35 + 3.12$$

$$E^{*red} = 2.77\ V$$

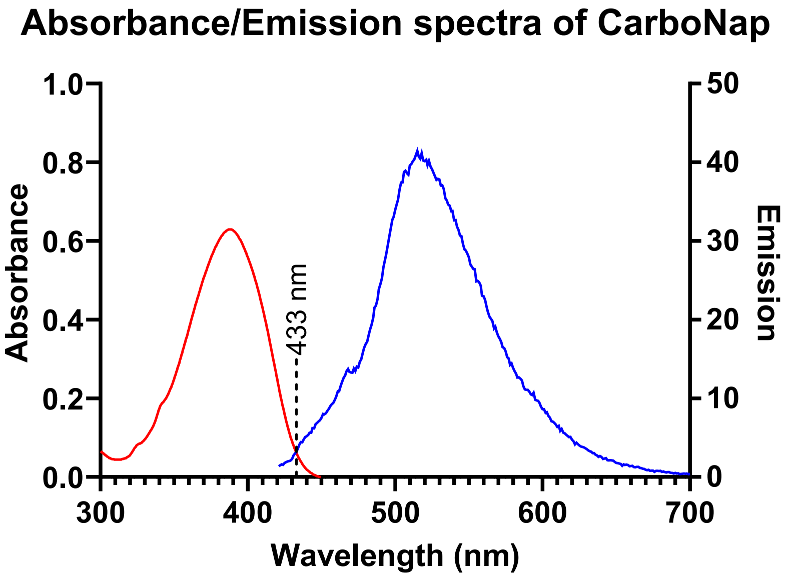

Figures S28. Absorbance and Emission spectra of **CarboNap** in DMF ([**CarboNap**]$_{em}$ = 5 µM; [**CarboNap**]$_{Abs}$ = 50 µM); λ_{exc} = 430 nm; slit widths 10 nm and 3.5 nm; $\lambda_{intersection}$ = 433 nm.

$$E^{*red} = E^{red} + E_{00}$$

$$E_{00} = \frac{hc}{\lambda}$$

$$E_{00} = \frac{1239.8}{433\ nm} = 2.86\ eV$$

Calculation for Excited state reduction potential, E*$_{red}$ for **CarboNap:**

$$E^{*red} = E^{red} + E_{00}$$

$$E^{*red} =- 0.005 + 2.86$$

$$E^{*red} = 2.86\ V$$

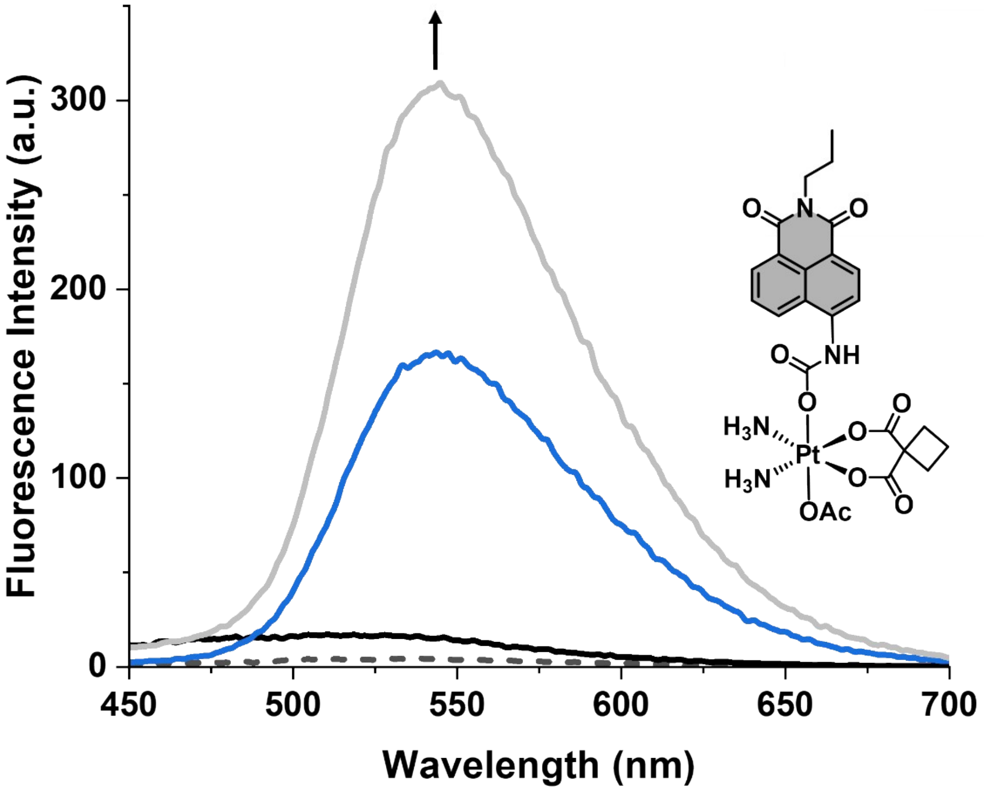

Figure S29. Fluorescence emission spectra of **CarboNap** (5 µM) incubated (1 h) under different conditions: control (dashed lines), NaAsc (10 mM, **black**), blue light only (**blue**) and NaAsc (10 mM) + blue light (grey) in PBS (pH = 7.4, 1 h). λ_{ex} = 430 nm; slit widths 10 nm and 3.5 nm.

Expanded Spectrum RT 0.16, NL 2289097, Peak [2], Target Mass 664.1164

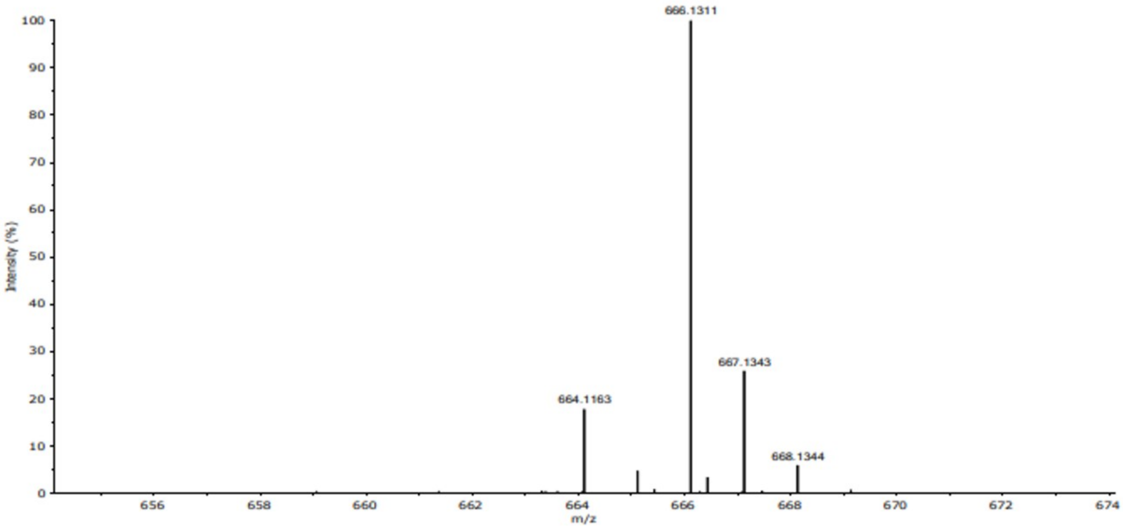

Theoretical Spectrum for C21H28N7O14P2, Minimum Abundance 0.01%

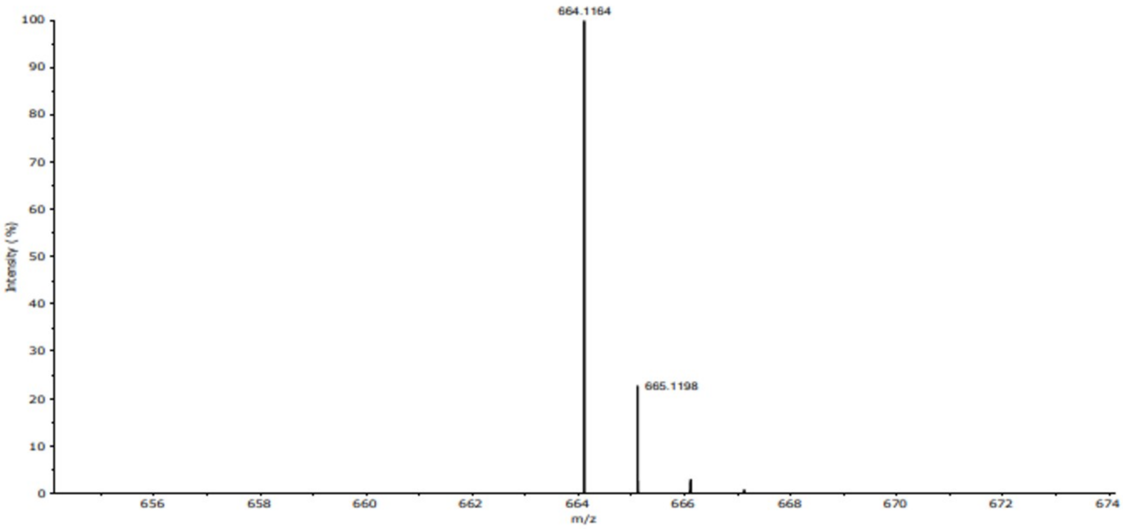

Measured Mass	Calculated Mass	Error (mDa)	Error (ppm)	Formula $[M]^+$	Response
664.1163	664.1164	-0.11	-0.16	$C_{21}H_{28}N_7O_{14}P_2$	722963

The measured m/z value is consistent with your proposed formula for this sample.

Figure S30. HRMS of NAD^+ (M^+, + ion mode) generated after **CarboBlue** (5 µM) was incubated with NADH (10 mM) and irradiated with blue light in H_2O (1 h).

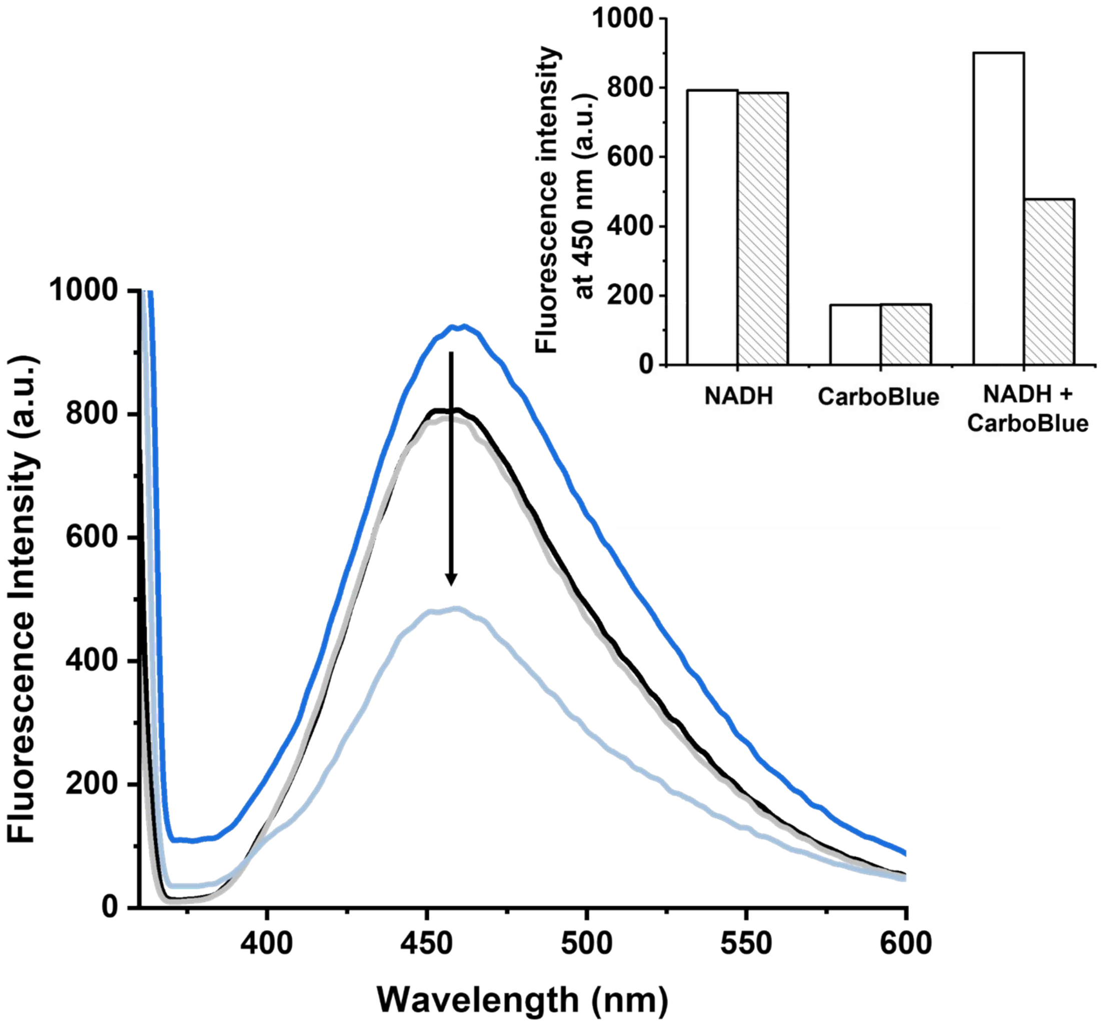

Figure S31. Fluorescence emission spectra of NADH (10 µM) irradiated with blue light at 0 h (black) and 3 h (grey); and NADH (10 µM) incubated with **CarboBlue** (5 µM) at 0 h (blue) and 3 h (shaded blue) in PBS buffer (pH 7.4, 0.01 M). λ_{ex} = 350 nm; slit widths 10 nm and 7.5 nm. Arrow represents decrease in NADH emission after incubation with **CarboBlue** and irradiated with light for 3 h. Inset: Bar chart showing fluorescence emission intensity at 450 nm for NADH; **CarboBlue**; and NADH + **CarboBlue** irradiated with blue light at 0 h (clear box) and 3 h (patterned box).

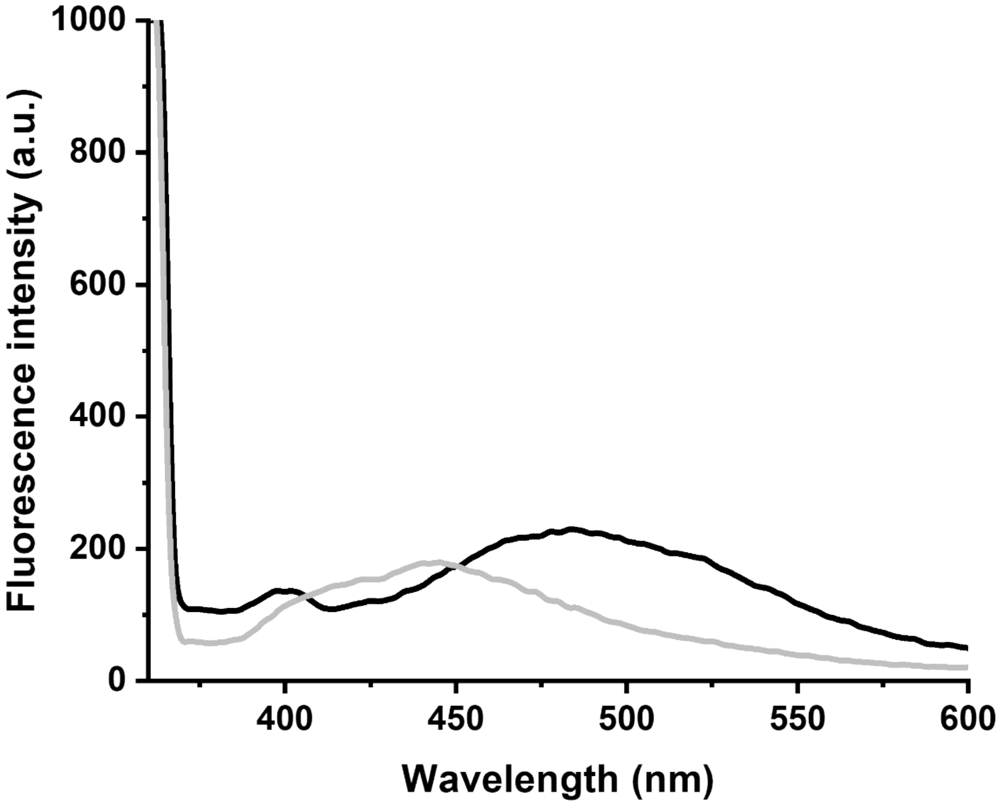

Figure S32. Fluorescence emission spectra of **CarboBlue** (5 µM) before (black) and after light irradiation (3 h, grey) in PBS buffer (pH 7.4). λ_{ex} = 350 nm; slit widths 10 nm and 7.5 nm.

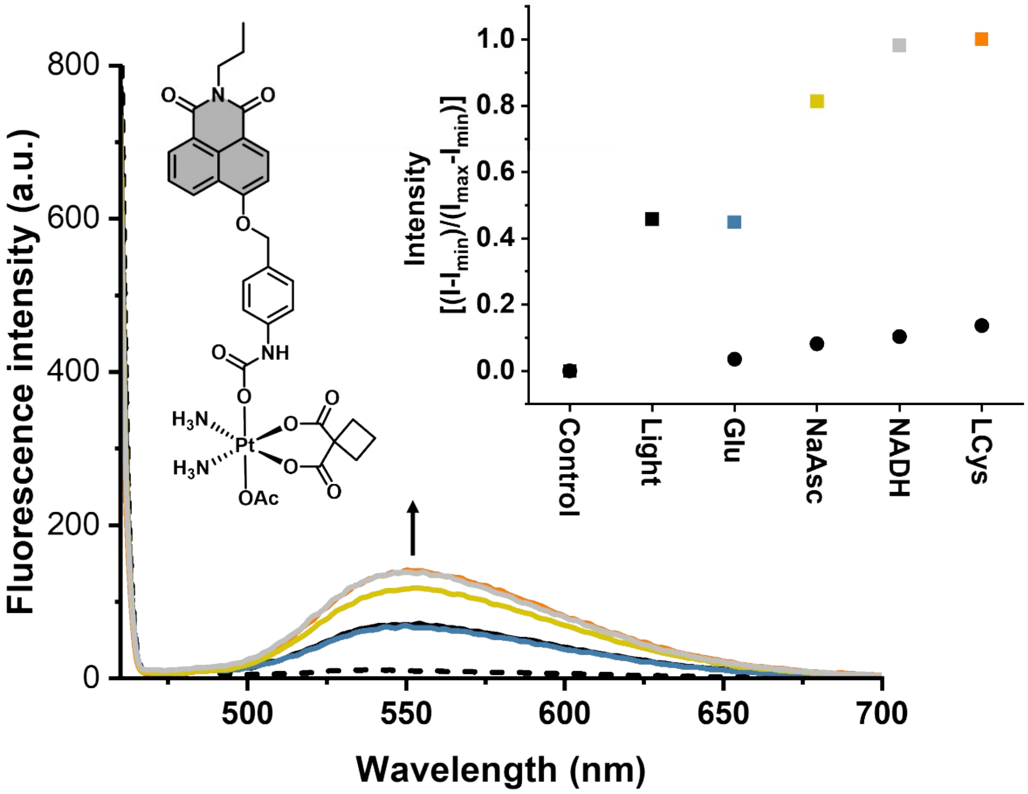

Figure S33. Fluorescence emission spectra of **CarboBlue** (5 µM) after 1 h irradiation with blue light and incubation with biological species (all 10mM): control (dashed lines), blue light (**black**), glucose + *hv* (**blue**), L-Cys + *hv* (**orange**), NaAsc + *hv* (**yellow**), and NADH + *hv* (**grey**). λ_{ex} = 450 nm; slit widths 10 nm and 5 nm. Inset: Changes in fluorescence intensity at 550 nm of **CarboBlue** in the presence of electron donors without (black circles) and with (coloured squares) light irradiation; data was normalised *via* maximum intensity, I_{max} (L-Cys + *hv*) and minimum intensity, I_{min} (control).

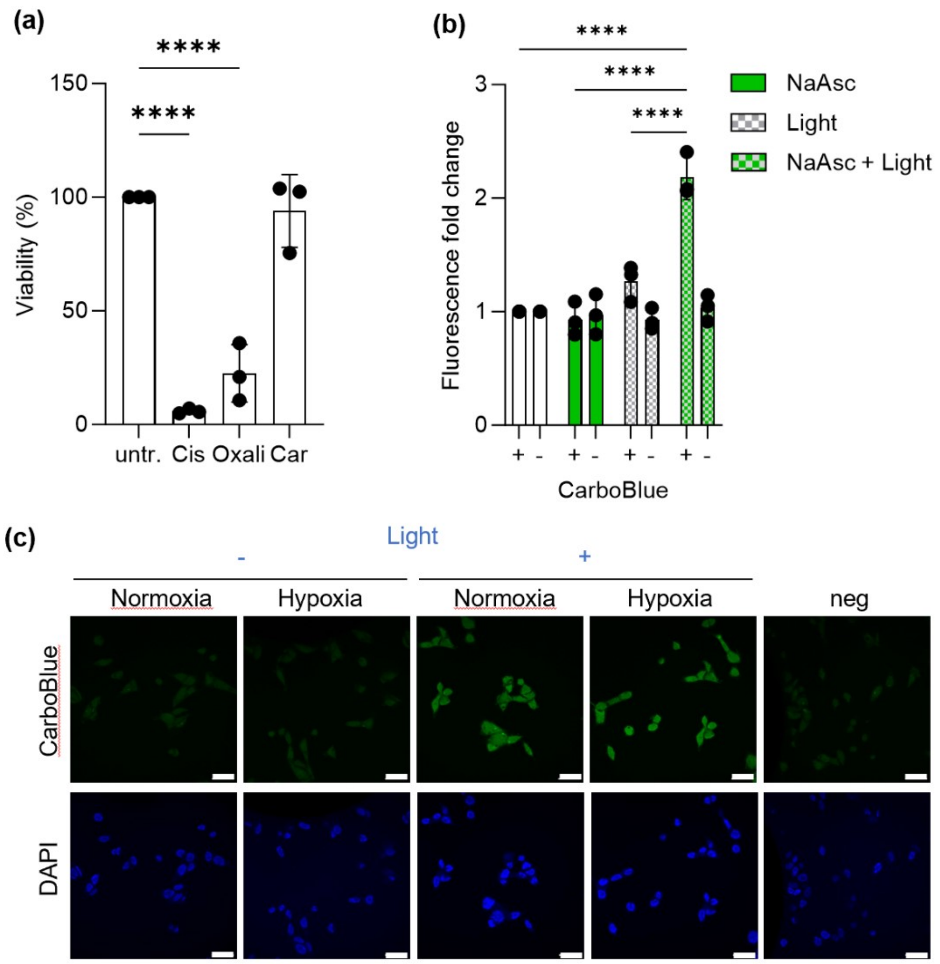

Figure S34. (a) HCT116 cells were treated with **CarboBlue**, **CisBlue** or **OxaliBlue** (10 µM) for 64 hours in normal conditions followed by an MTT assay. Untr. = untreated cells. Error bars represent SD. **** = $p < 0.0001$. n = 3. (b) Flow cytometry analysis of HCT116 cells untreated (-) or treated (+) with **CarboBlue** (10 µM) after treatment with NaAsc (2 mM, 2 hours, green) and light exposure (30 mins, check pattern). Fluorescence fold change of the geometric mean intensity is displayed. Data is normalised to the untreated condition. Error bars represent SD. **** = $p < 0.0001$. n=3. (c) Representative fluorescence images of HCT116 cells untreated (neg) and treated with CarboBlue (10 µM), either exposed to normoxia (21% O_2) or hypoxia (<0.1% O_2) and irradiated with blue light (30 mins). Scale bar represents 12 µm.

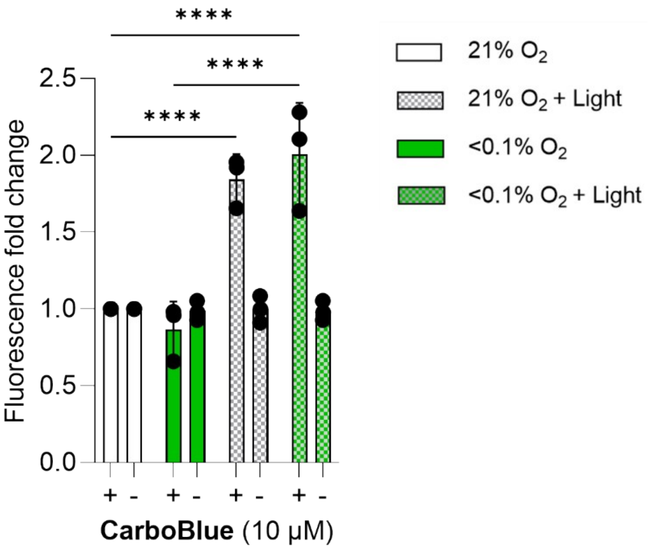

Figure S35. Flow cytometry analysis of HCT116 cells treated with CarboBlue (10 µM) under normoxic (21 % O_2, 16 hours) or hypoxic conditions (<0.1% O_2, 16 hours) with light exposure (30 mins). Fluorescence fold change of the geometric mean intensity is displayed. Data is normalised to the normoxic condition (21% O_2). Error bars represent SD. **** = $p < 0.0001$. n = 3.

4. NMR and HRMS Spectra

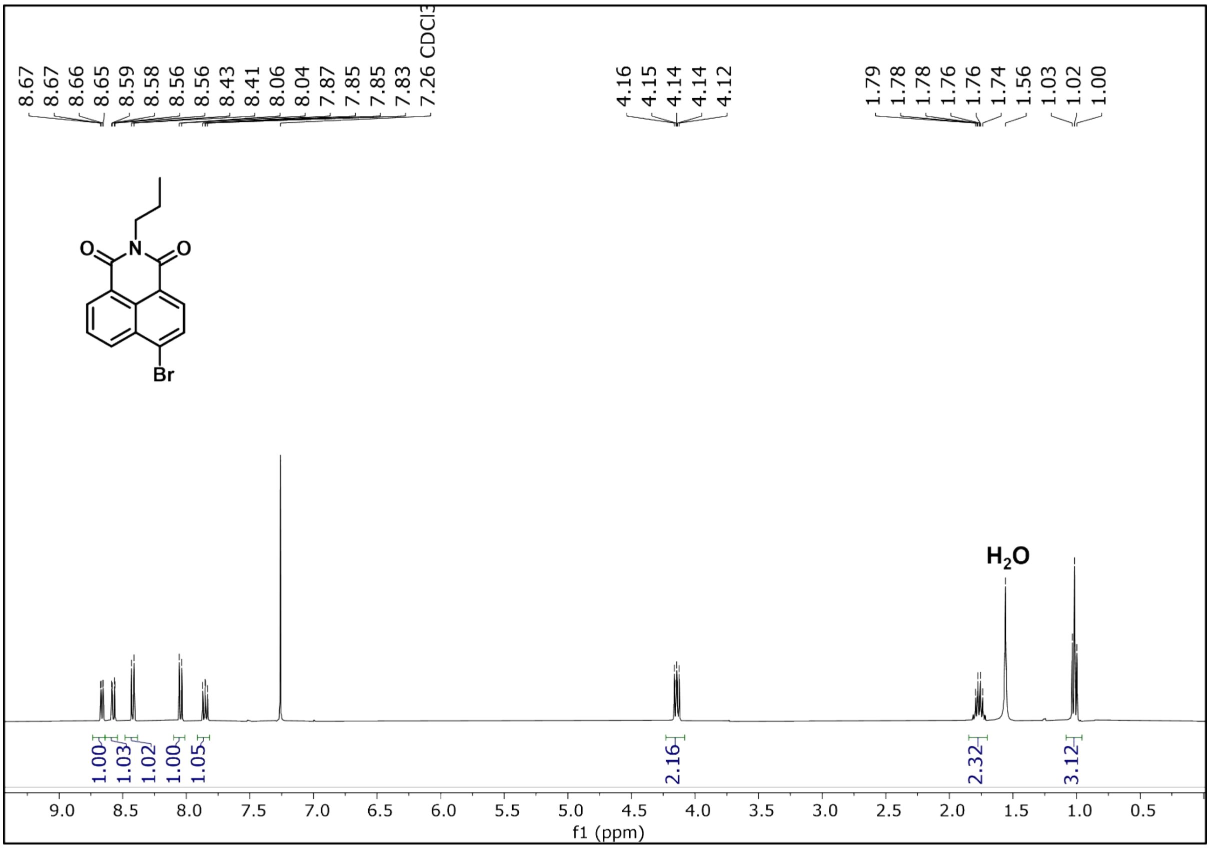

Figure S36. ^{1}H NMR (400 MHz, $CDCl_3$) at 25 °C of **Nap-Br**.

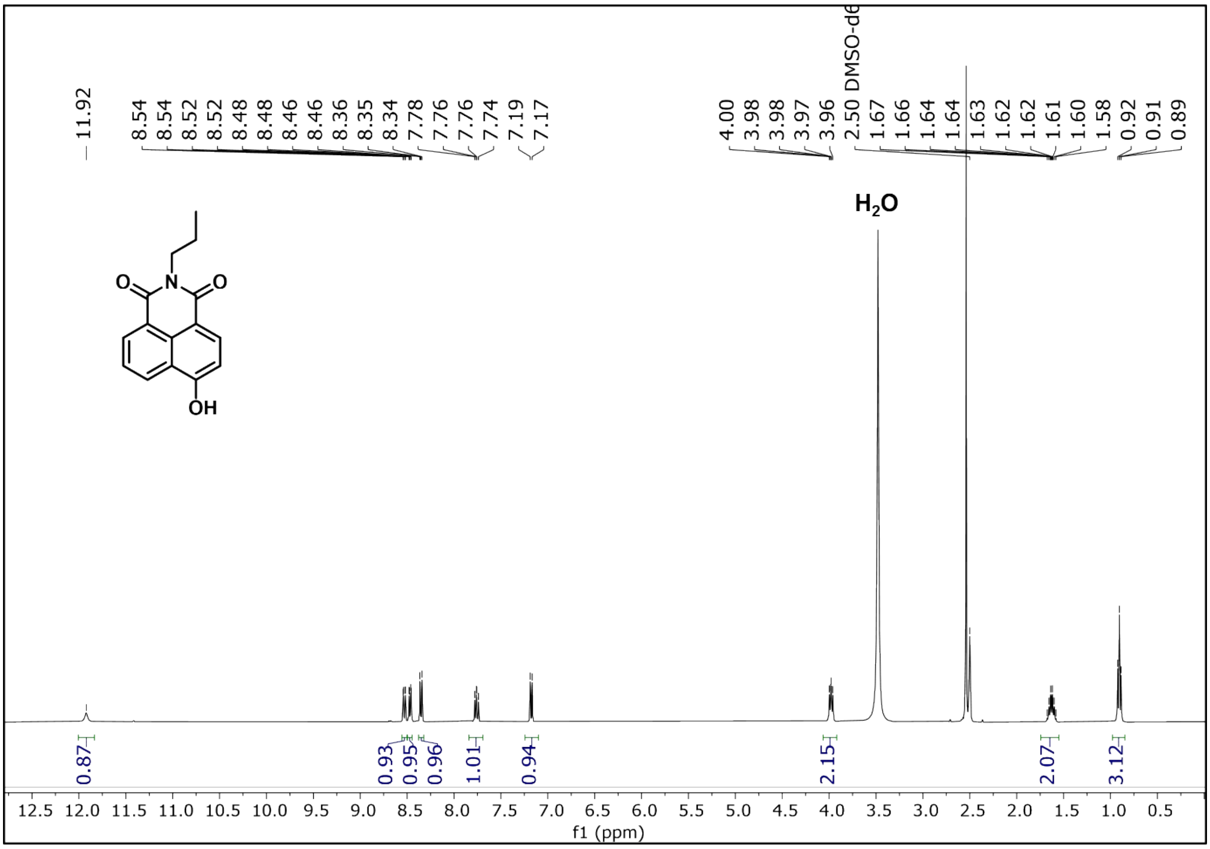

Figure S37. ^{1}H NMR (400 MHz, DMSO-d_6) at 25 °C of **Nap-OH**.

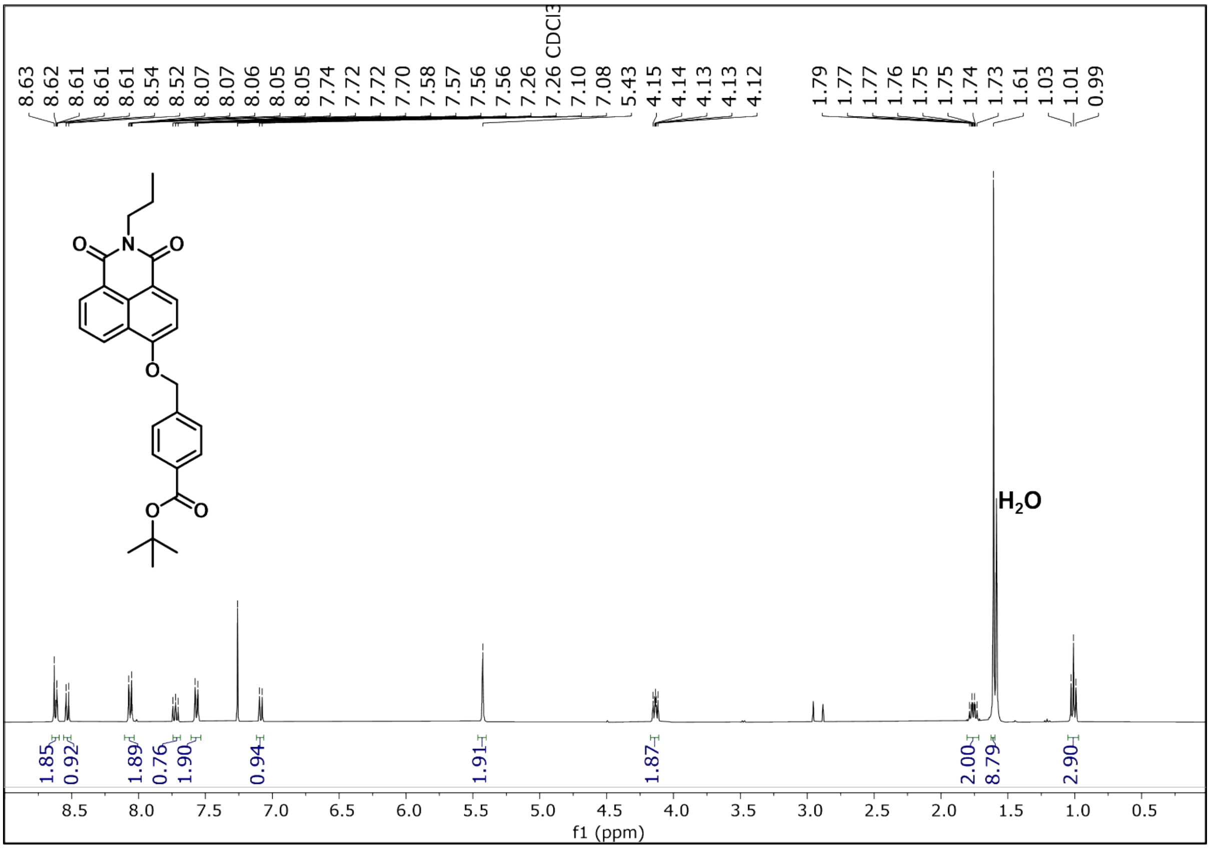

Figure S38. ^{1}H NMR (400 MHz, $CDCl_3$) at 25 °C of **Nap-Bn-Tb**.

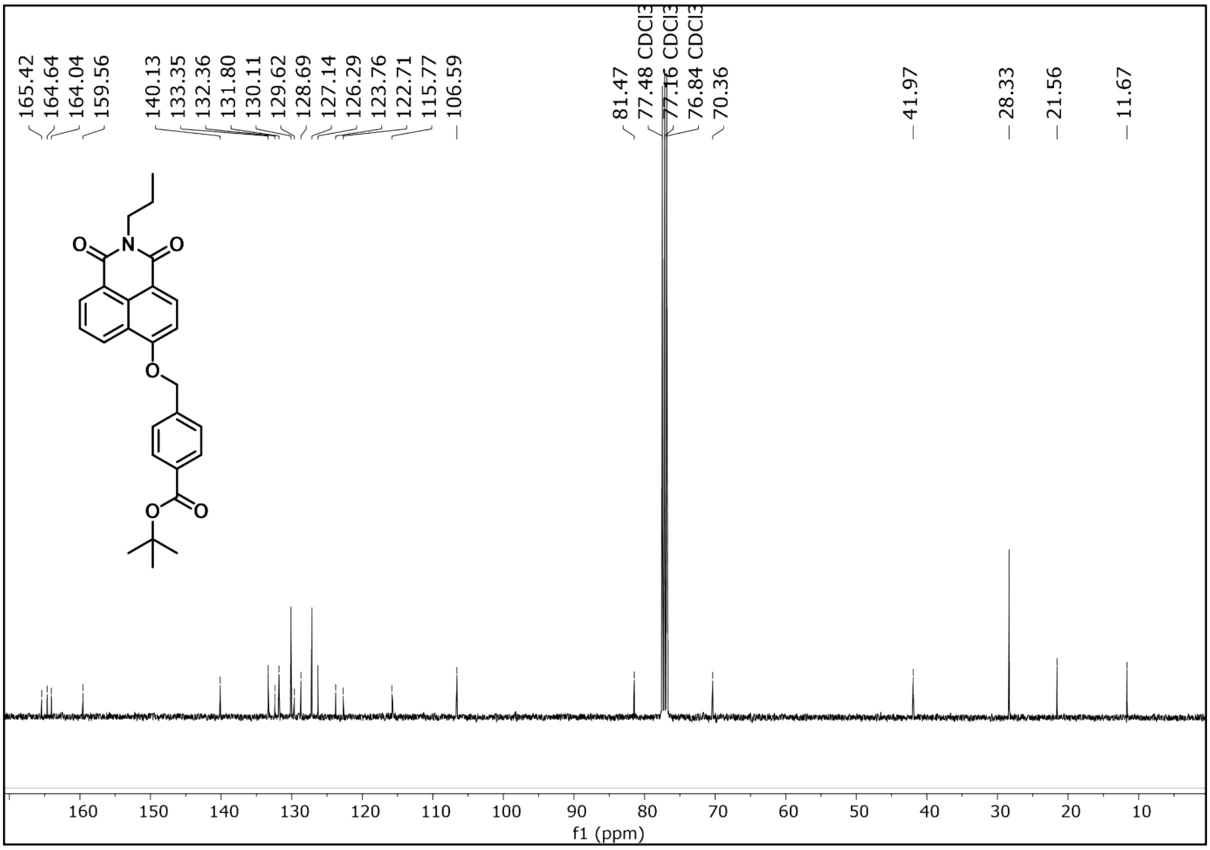

Figure S39. ^{13}C NMR (400 MHz, $CDCl_3$) at 25 °C of **Nap-Bn-Tb**.

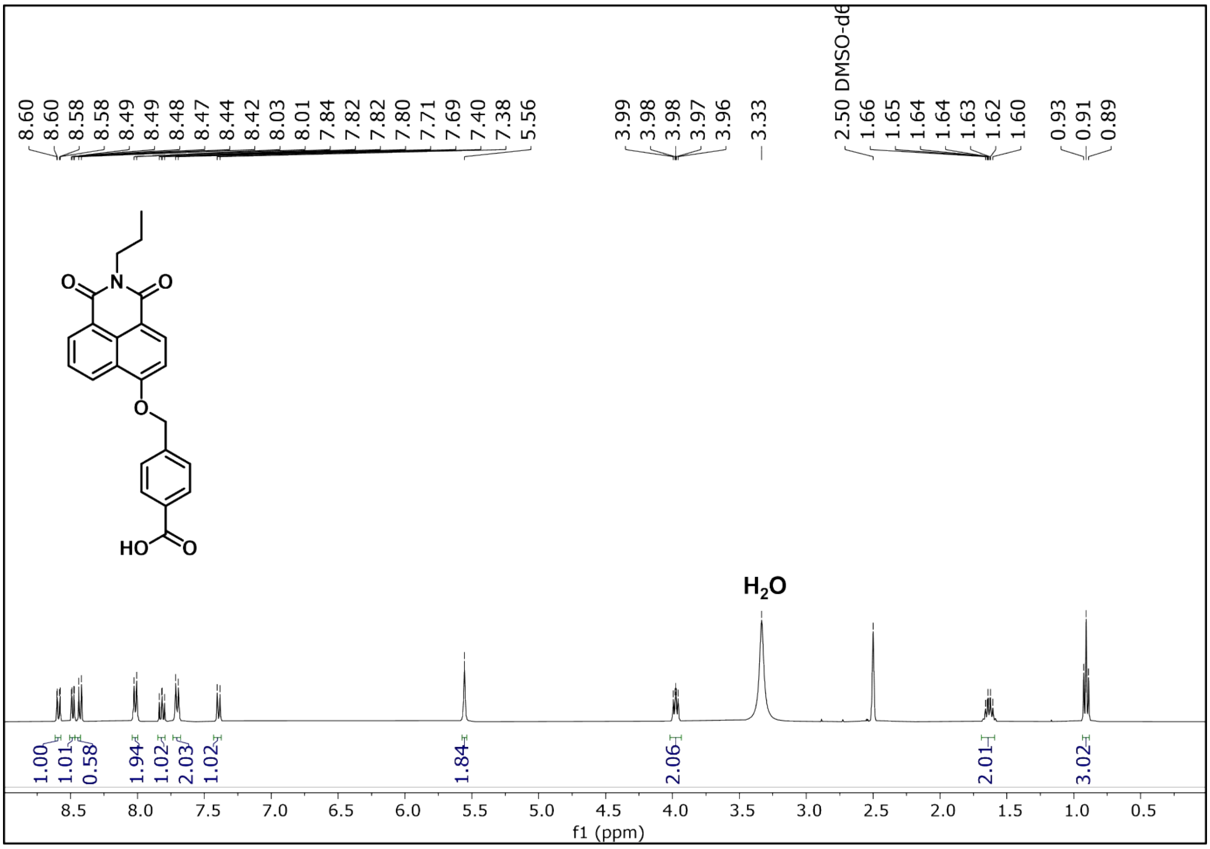

Figure S40. ^1H NMR (400 MHz, DMSO-d_6) at 25 °C of **Nap-Bn-COOH**.

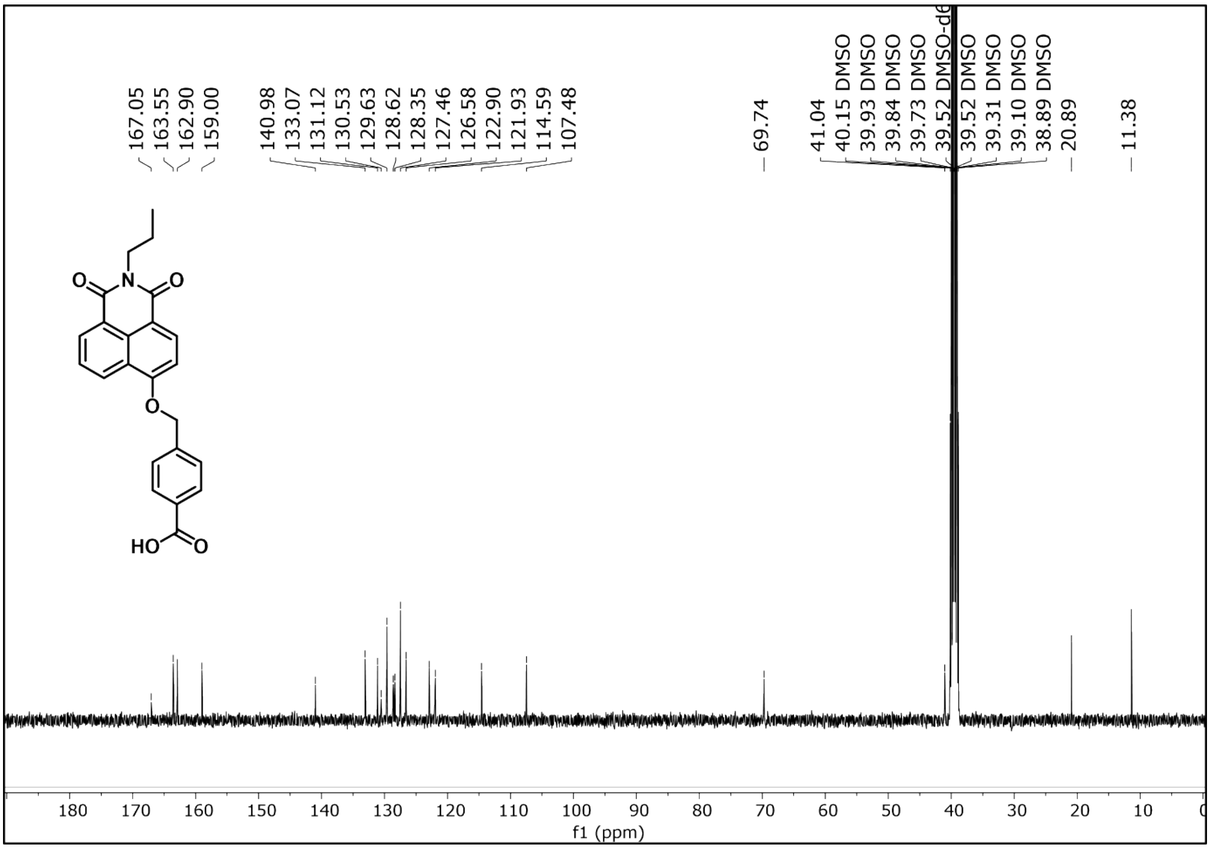

Figure S41. ^{13}C NMR (400 MHz, DMSO-d_6) at 25 °C of **Nap-Bn-COOH**.

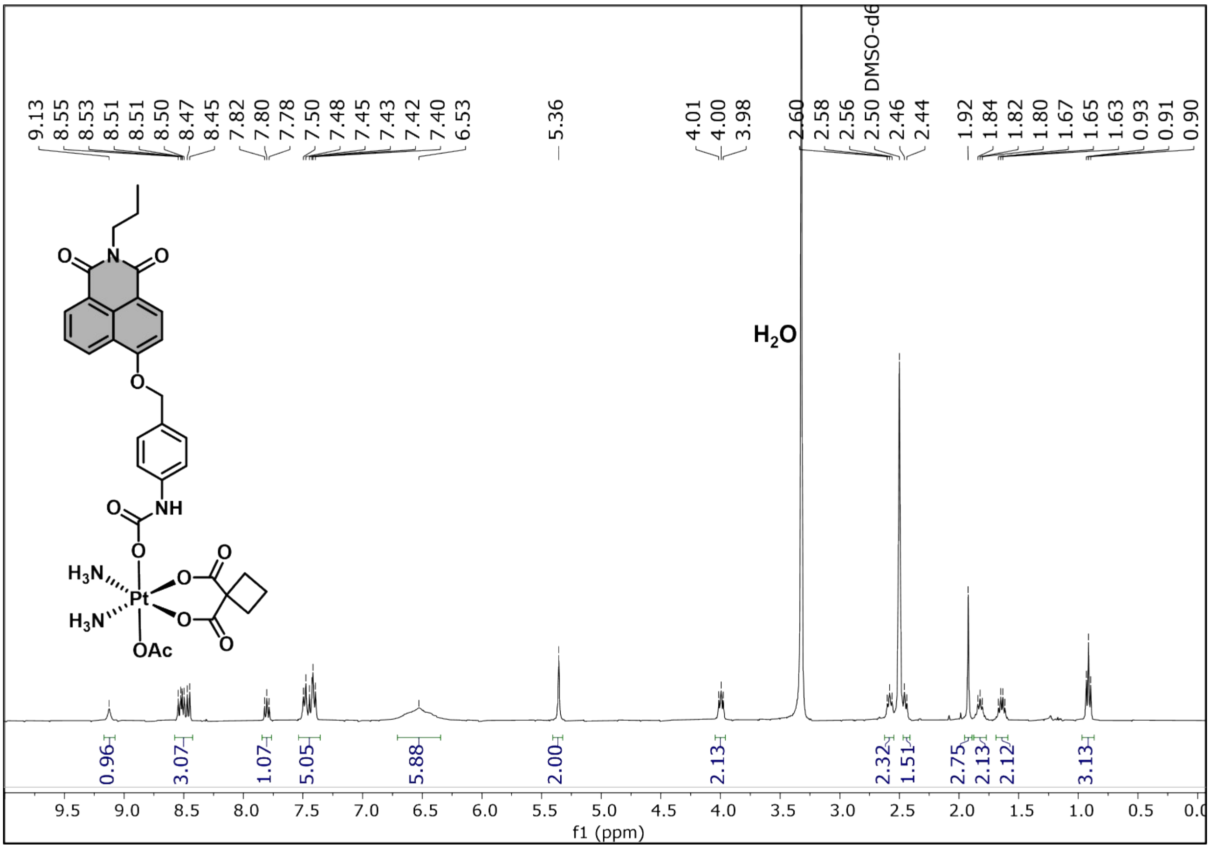

Figure S42. ^1H NMR (400 MHz, DMSO-d_6) at 25 °C of **CarboBlue**.

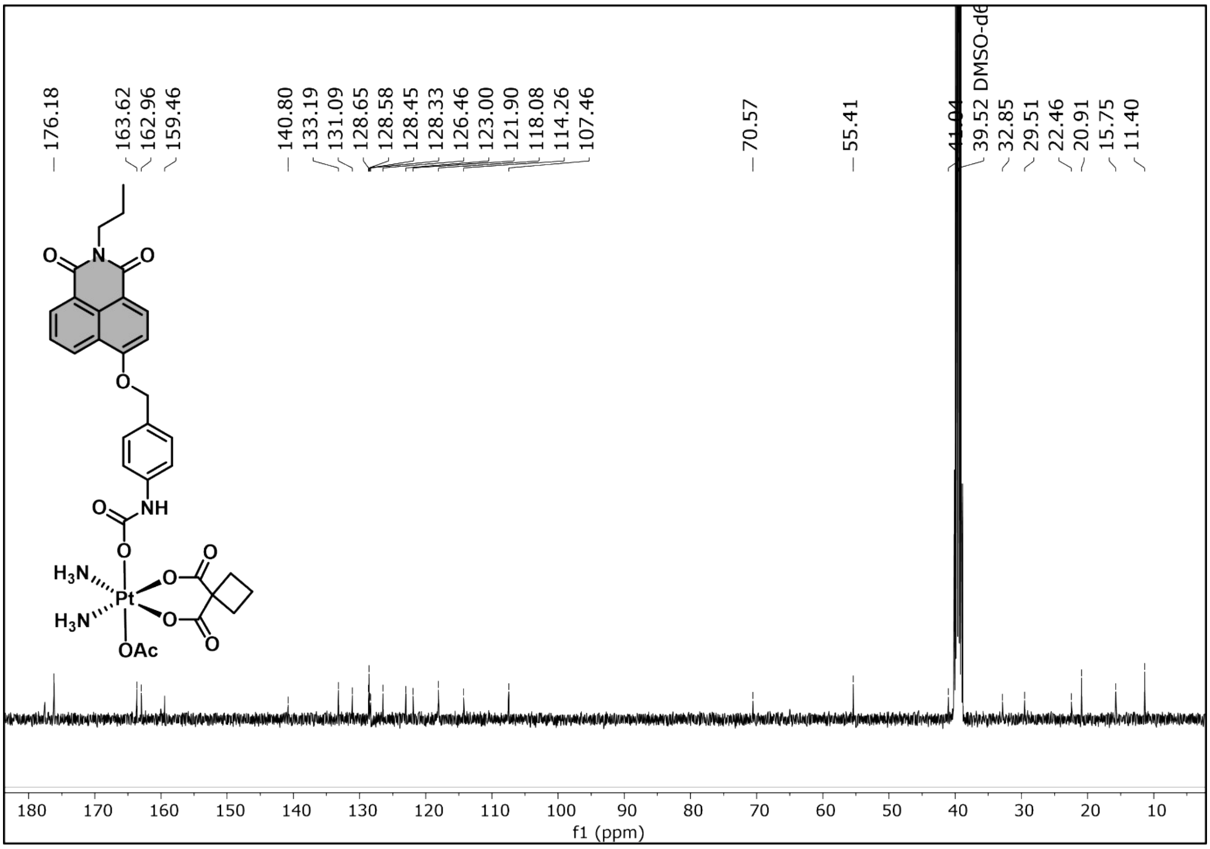

Figure S43. ^{13}C NMR (151 MHz, DMSO-d_6) at 25 °C of **CarboBlue**.

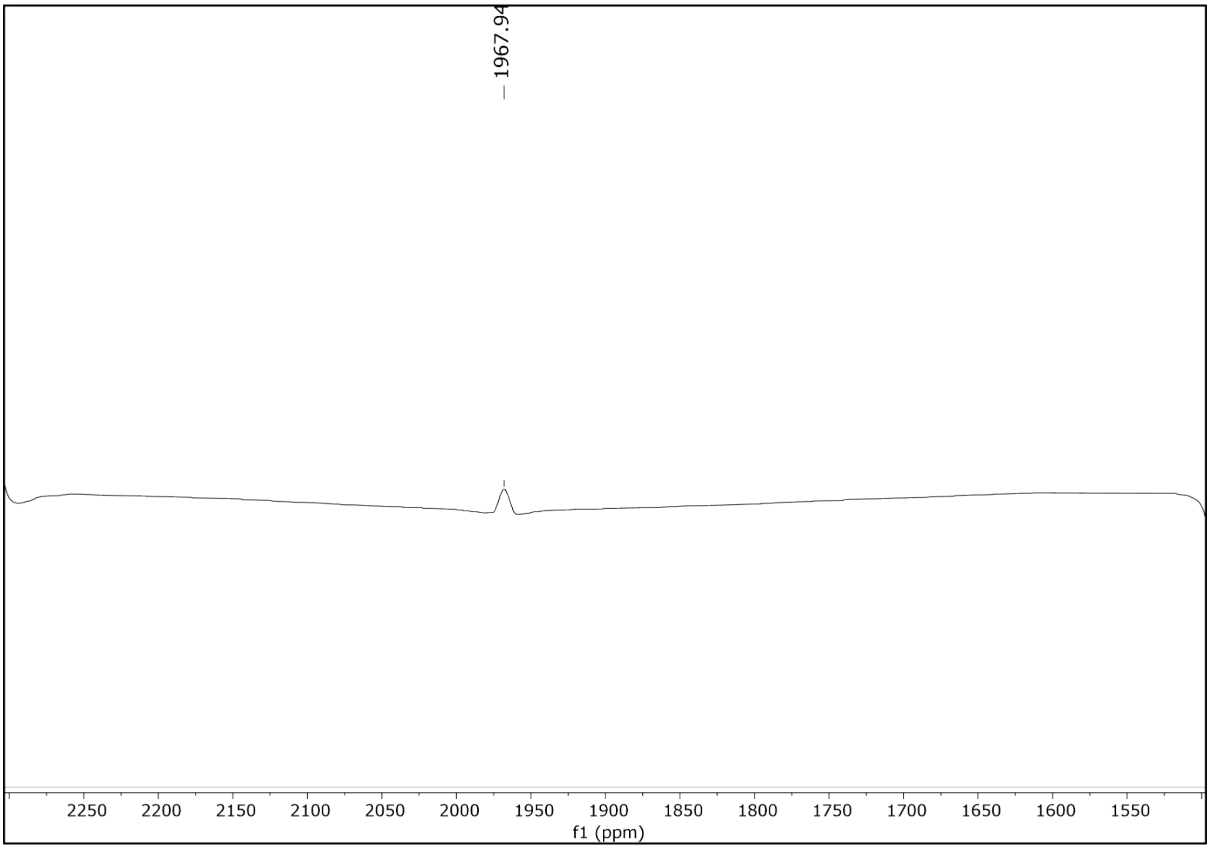

Figure S44. ^{195}Pt NMR (86 MHz, DMSO-d_6) of **CarboBlue**.

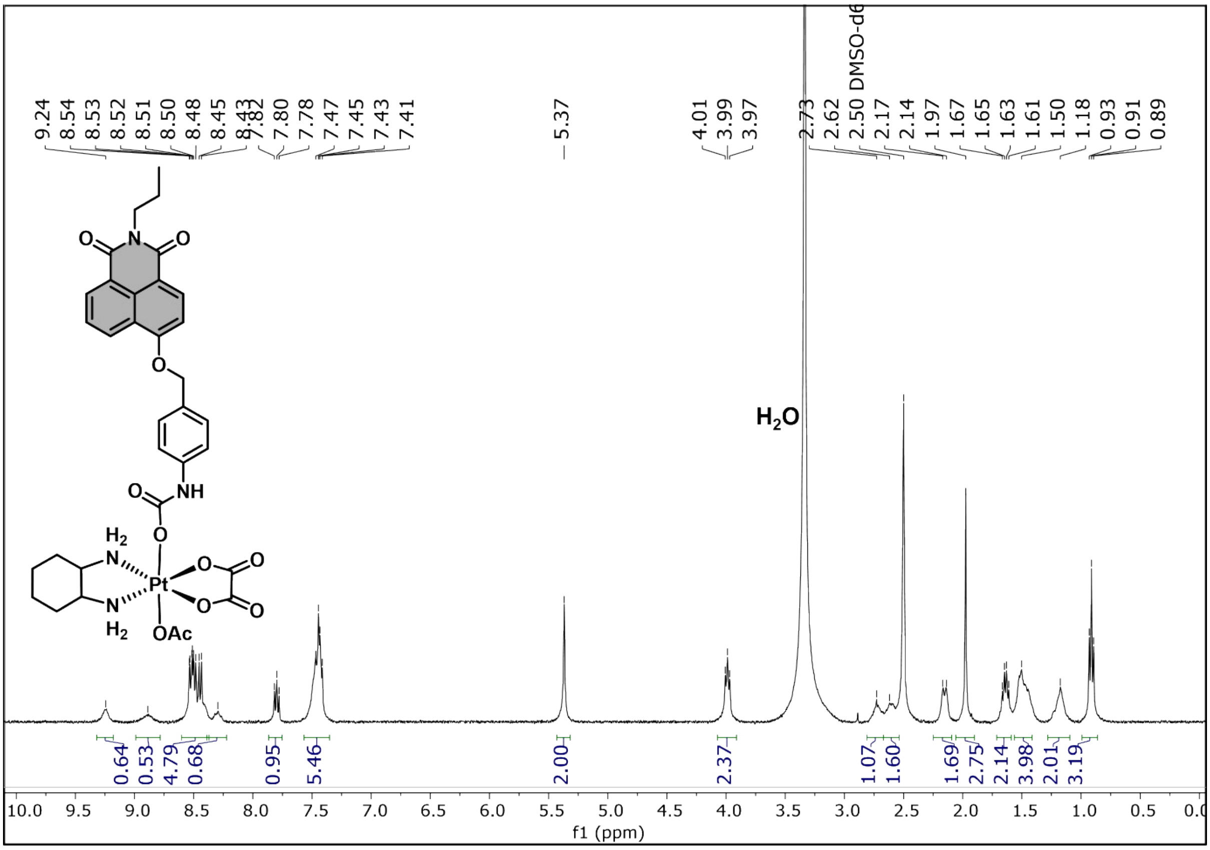

Figure S45. ^{1}H NMR (400 MHz, DMSO-d_6) at 25 °C of **OxaliBlue**.

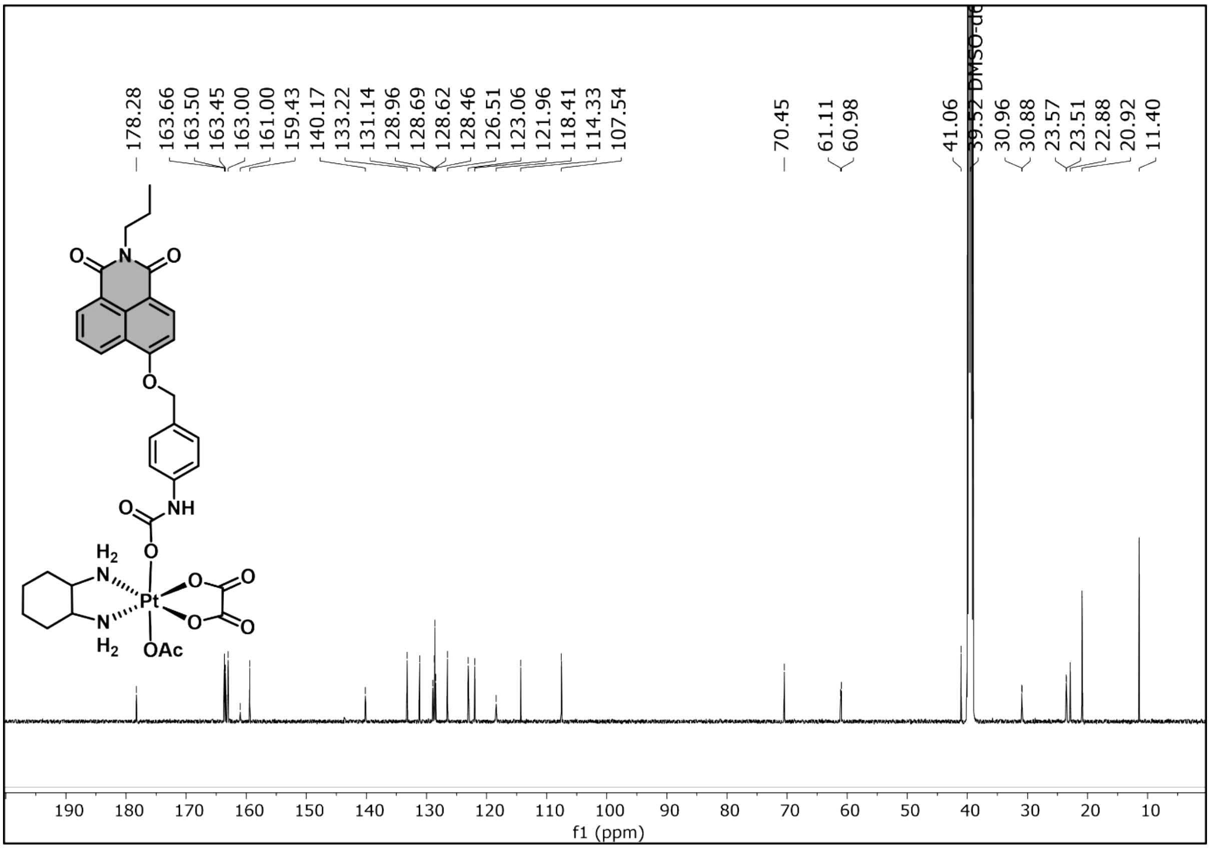

Figure S46. ^{13}C NMR (151 MHz, DMSO-d_6) at 25 °C of **OxaliBlue**.

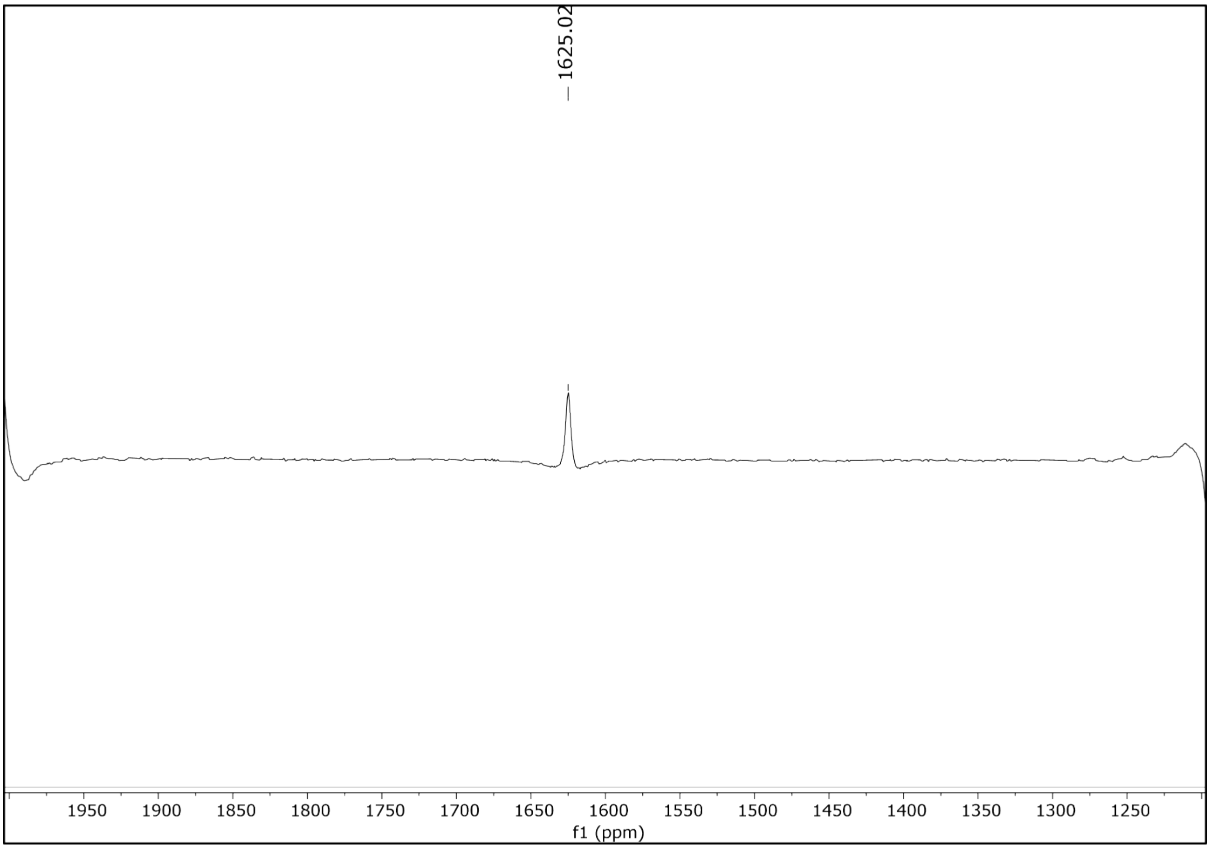

Figure S47. ^{195}Pt NMR (86 MHz, DMSO-d_6) of **OxaliBlue**.

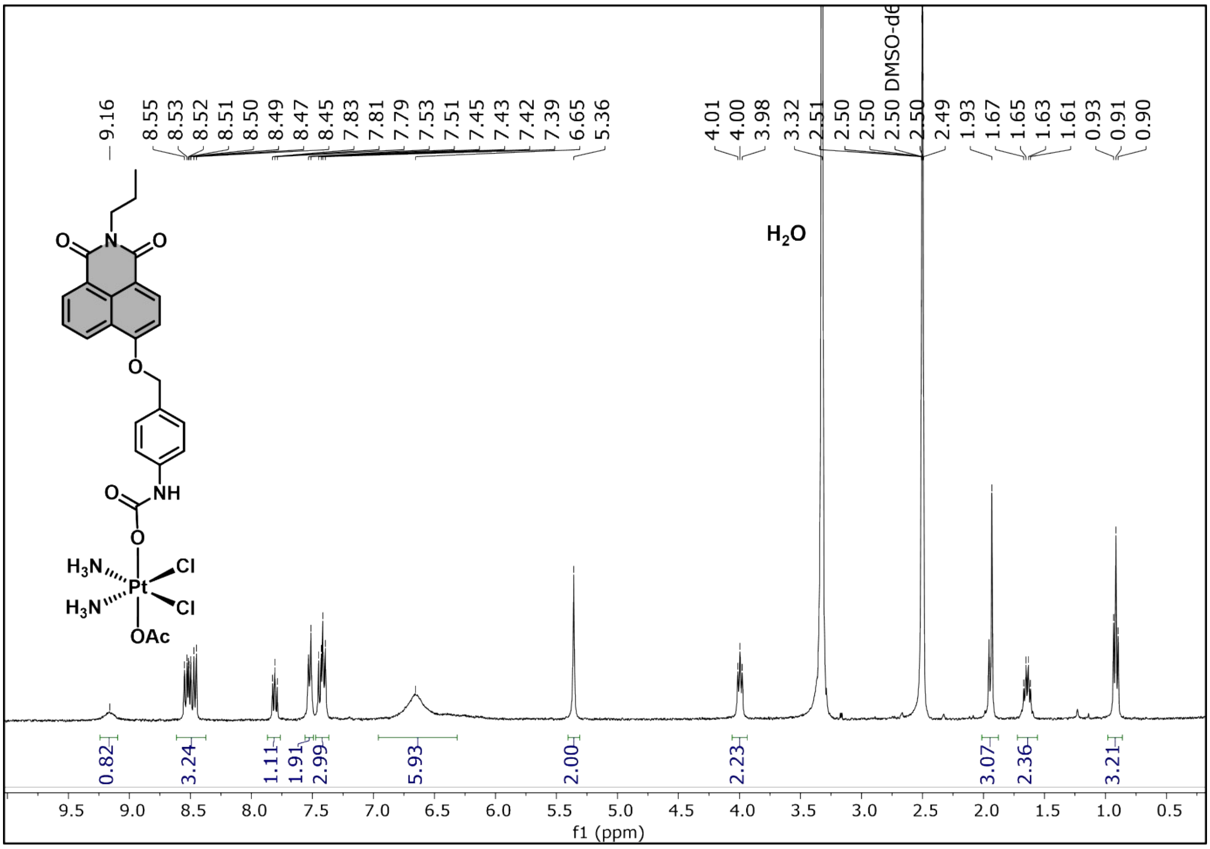

Figure S48. ^{1}H NMR (400 MHz, DMSO-d$_6$) at 25 °C of **CisBlue**.

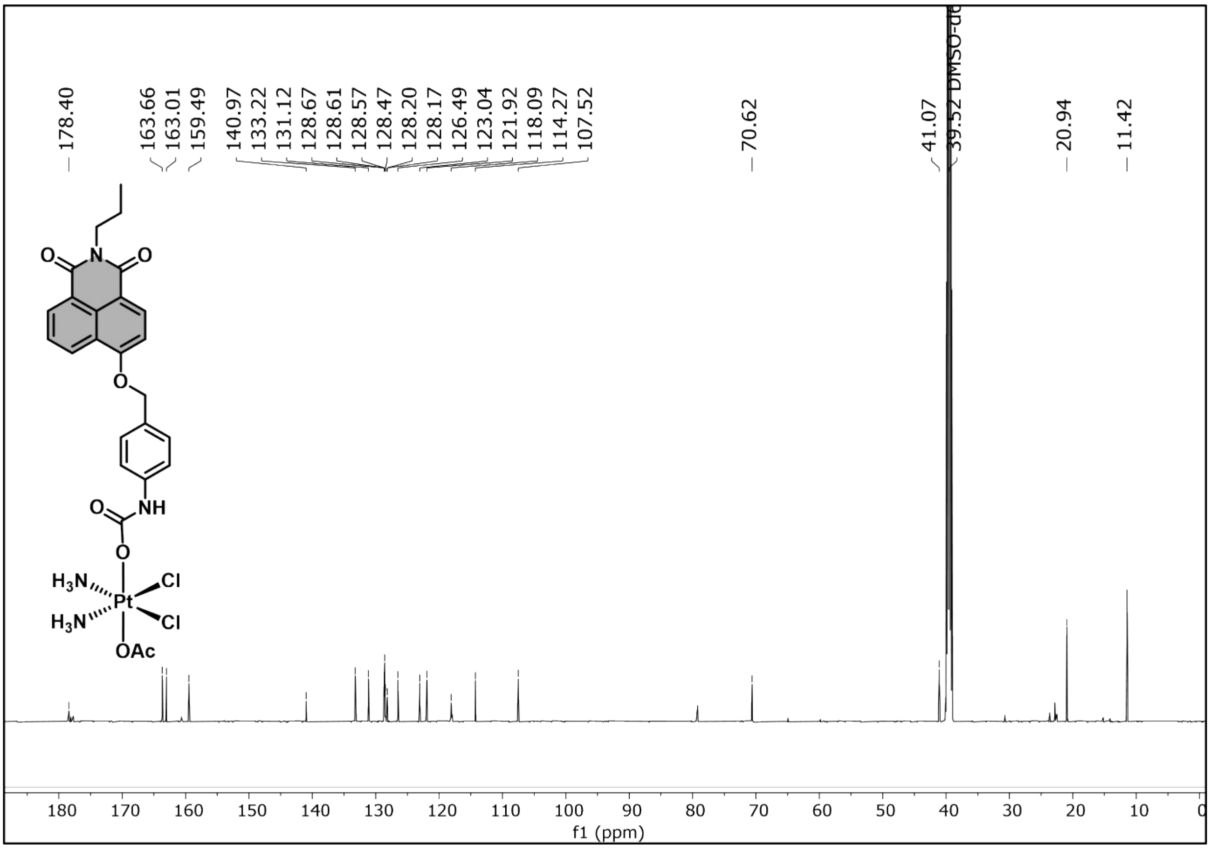

Figure S49. ^{13}C NMR (151 MHz, DMSO-d_6) at 25 °C of **CisBlue**.

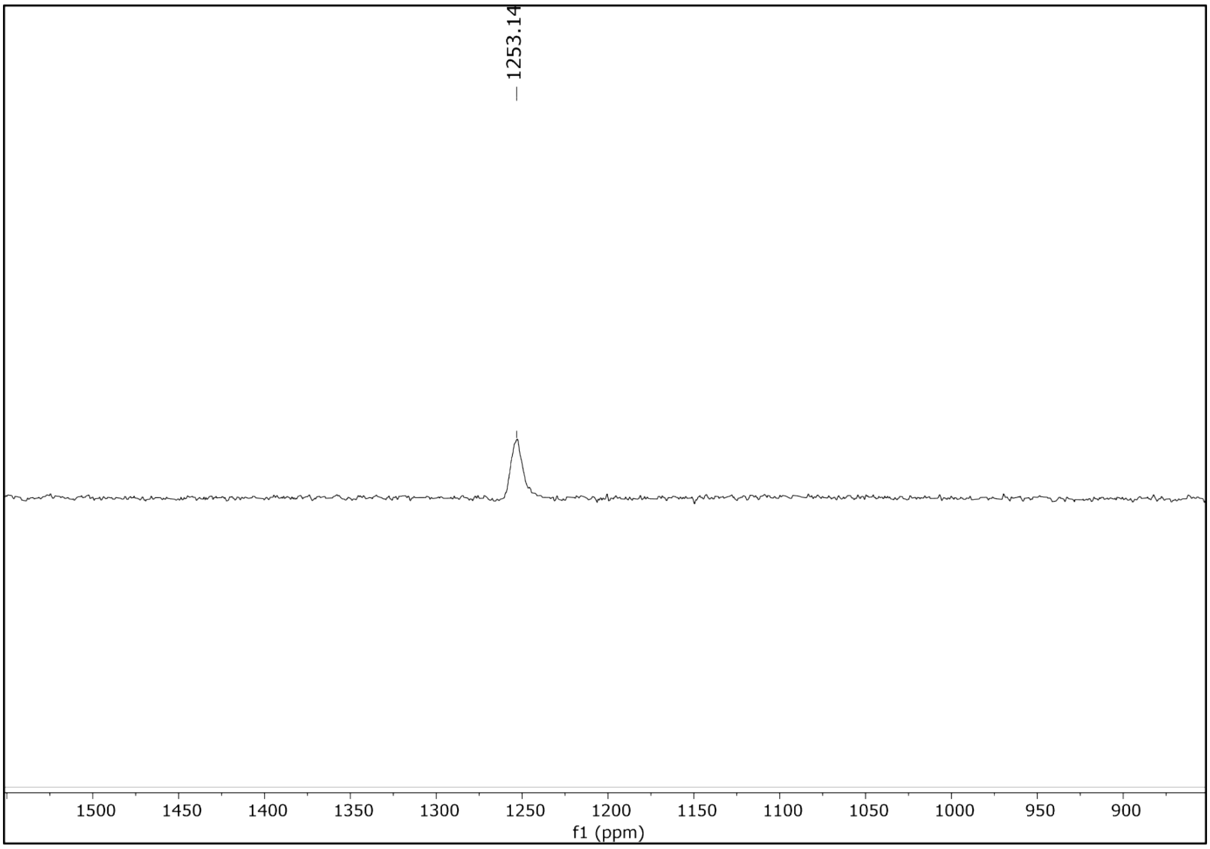

Figure S50. ^{195}Pt NMR (86 MHz, DMSO-d_6) at 25 °C of **CisBlue**.

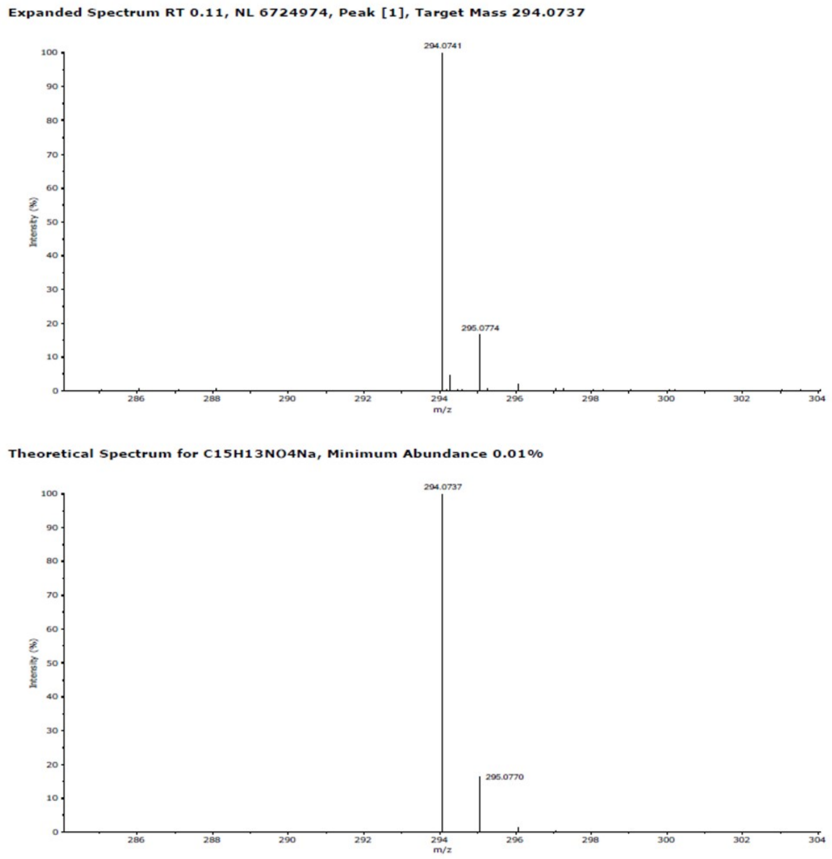

Measured Mass	Calculated Mass	Error (mDa)	Error (ppm)	Formula $[M+Na]^+$	Response
294.0741	294.0737	0.42	1.41	$C_{15}H_{13}NO_4Na$	2690818

The measured m/z value is consistent with your proposed formula for this sample.

Figure S51. HRMS of **Nap-OH** (Na adduct, + ion mode) in MeOH (+ 1% DMSO).

Expanded Spectrum RT 0.08, NL 16880444, Peak [1], Target Mass 446.1962

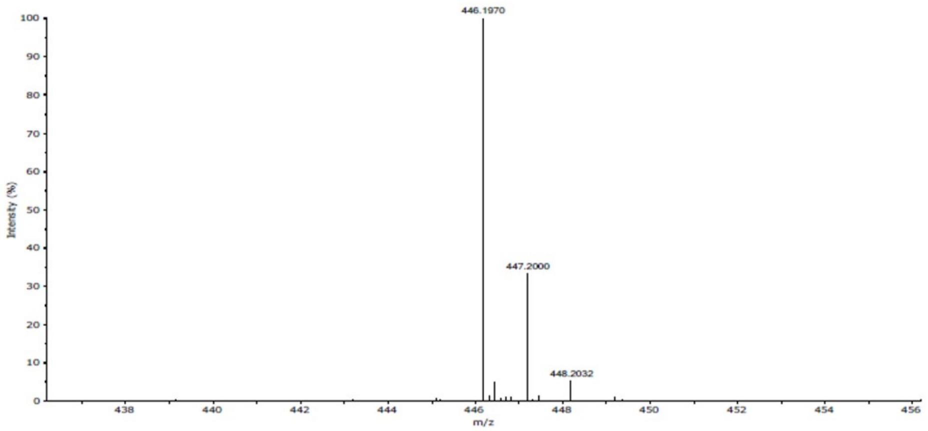

Theoretical Spectrum for C27H28NO5, Minimum Abundance 0.01%

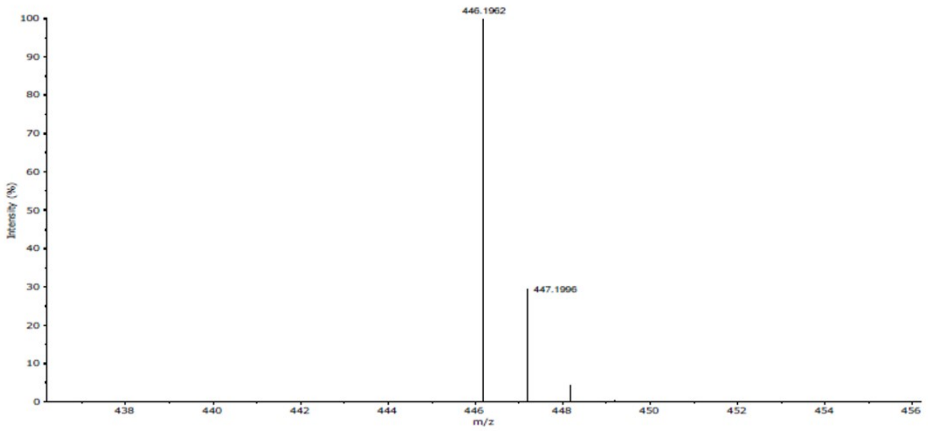

Measured Mass	Calculated Mass	Error (mDa)	Error (ppm)	Formula $[M+H]^+$	Response
446.1970	446.1962	0.80	1.78	$C_{27}H_{28}NO_5$	12503072

The measured m/z value is consistent with your proposed formula for this sample.

Figure S52. HRMS of **Nap-Bn-Tb** (H adduct, + ion mode) in CH_2Cl_2.

Expanded Spectrum RT 0.08, NL 3163337, Peak [1], Target Mass 390.1336

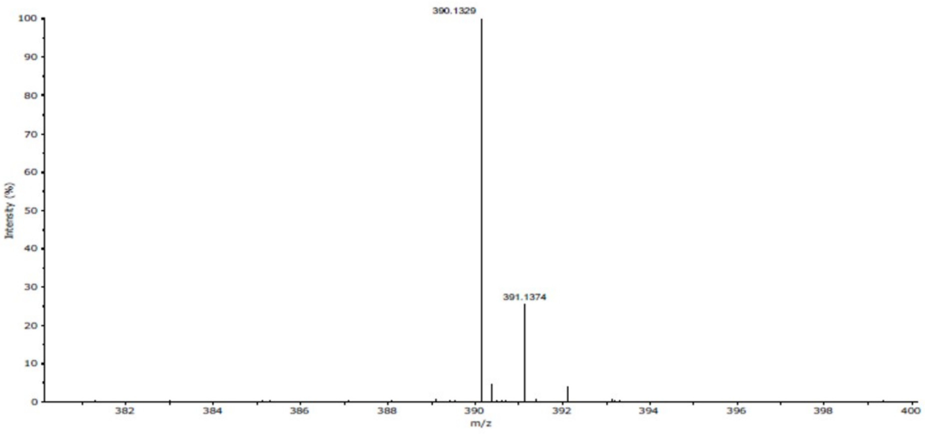

Theoretical Spectrum for C23H20NO5, Minimum Abundance 0.01%

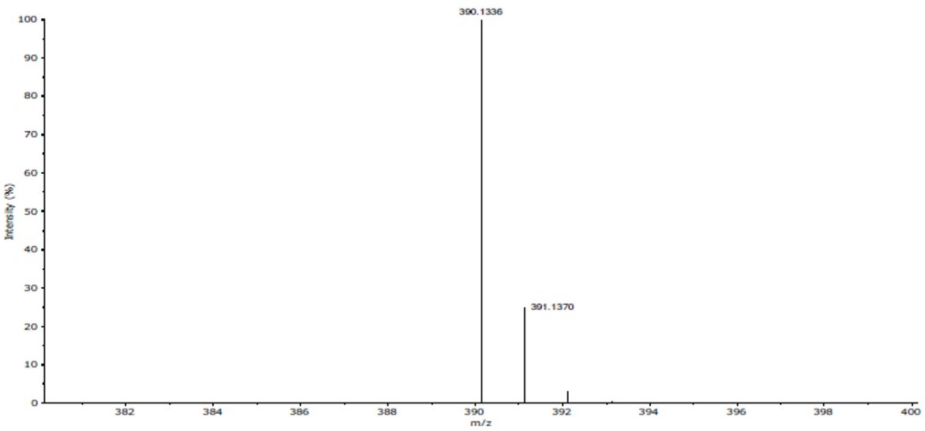

Measured Mass	Calculated Mass	Error (mDa)	Error (ppm)	Formula $[M+H]^+$	Response
390.1329	390.1336	-0.70	-1.81	$C_{23}H_{20}NO_5$	6647726

The measured m/z value is consistent with your proposed formula for this sample.

Figure S53. HRMS of **Nap-Bn-COOH** (H adduct, + ion mode) in MeOH (+ 1% DMSO).

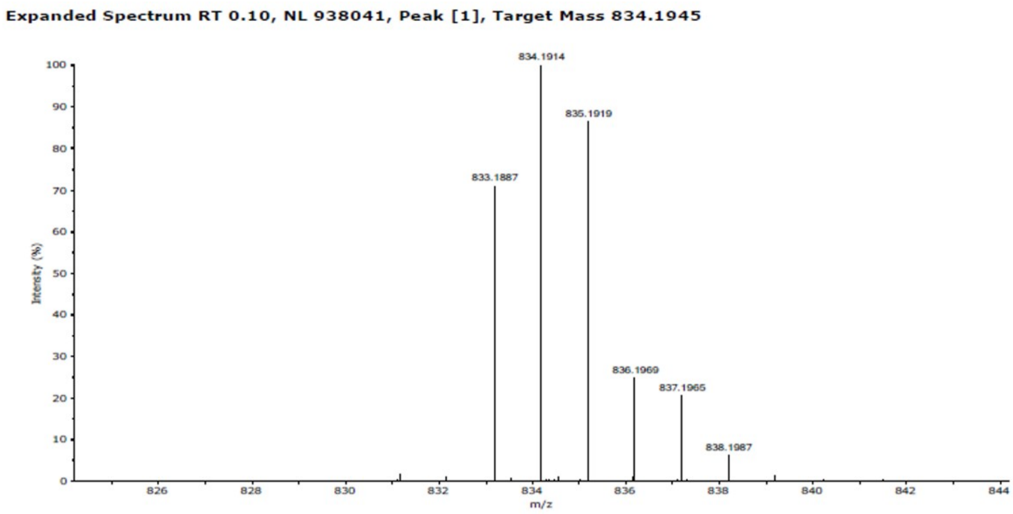

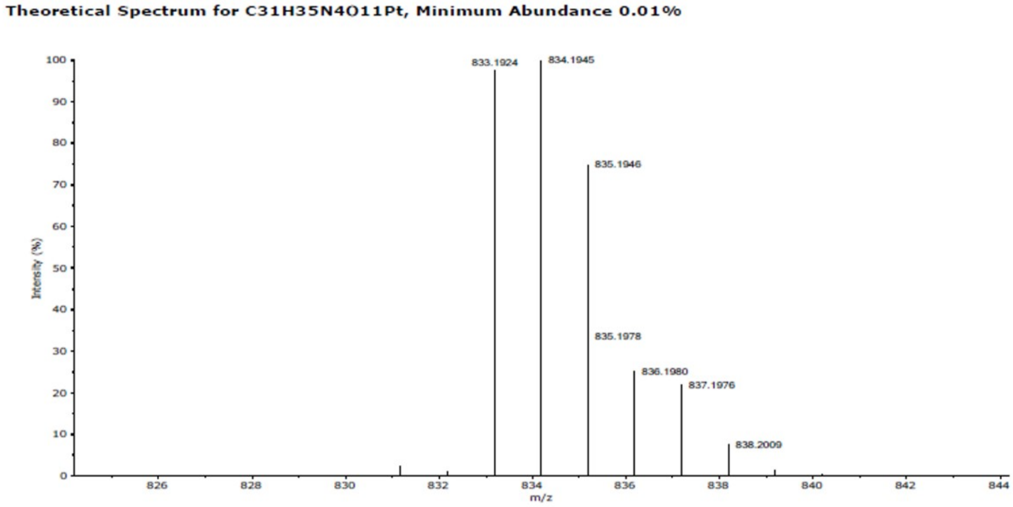

Measured Mass	Calculated Mass	Error (mDa)	Error (ppm)	Formula [M+H]$^+$	Response
834.1914	834.1945	-3.08	-3.69	$C_{31}H_{35}N_4O_{11}Pt$	7603772

The measured m/z value is consistent with your proposed formula for this sample.

Figure S54. HRMS of **CarboBlue** (H adduct, + ion mode) in MeOH (+ 1% DMSO).

Expanded Spectrum RT 0.09, NL 748285, Peak [1], Target Mass 860.2101

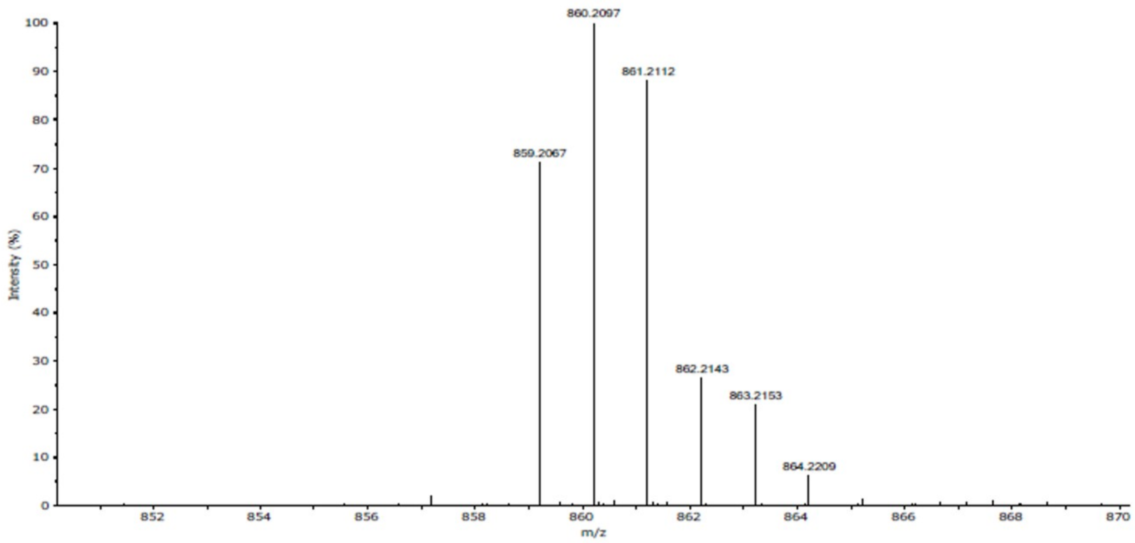

Theoretical Spectrum for C33H37N4O11Pt, Minimum Abundance 0.01%

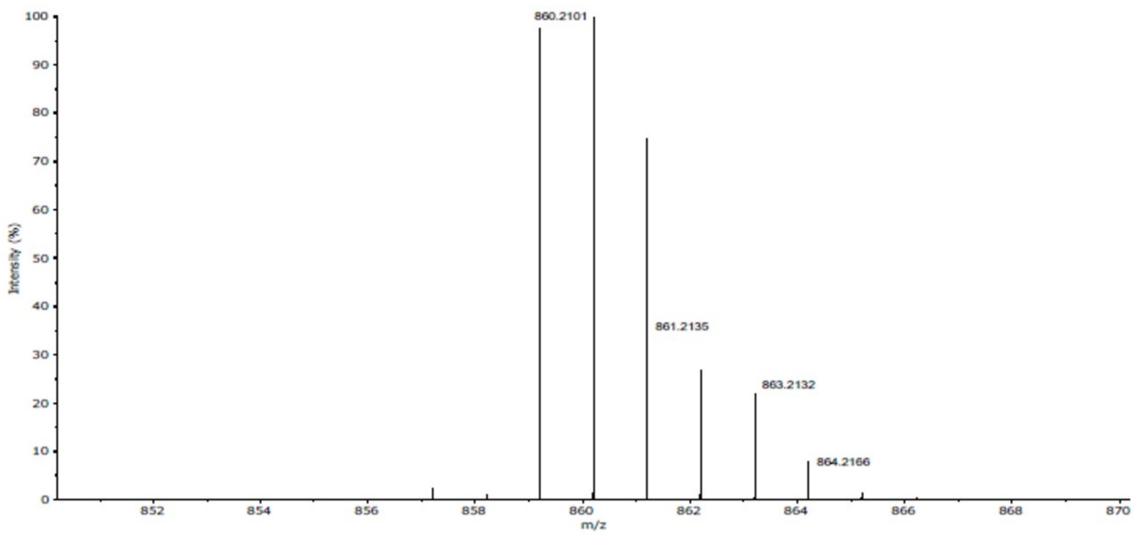

Measured Mass	Calculated Mass	Error (mDa)	Error (ppm)	Formula $[M+H]^+$	Response
860.2097	860.2101	-0.43	-0.50	$C_{33}H_{37}N_4O_{11}Pt$	3377944

The measured m/z value is consistent with your proposed formula for this sample.

Figure S55. HRMS of **OxaliBlue** (H adduct, + ion mode) in MeOH (+ 1% DMSO).

Expanded Spectrum RT 0.09, NL 879289, Peak [1], Target Mass 762.1056

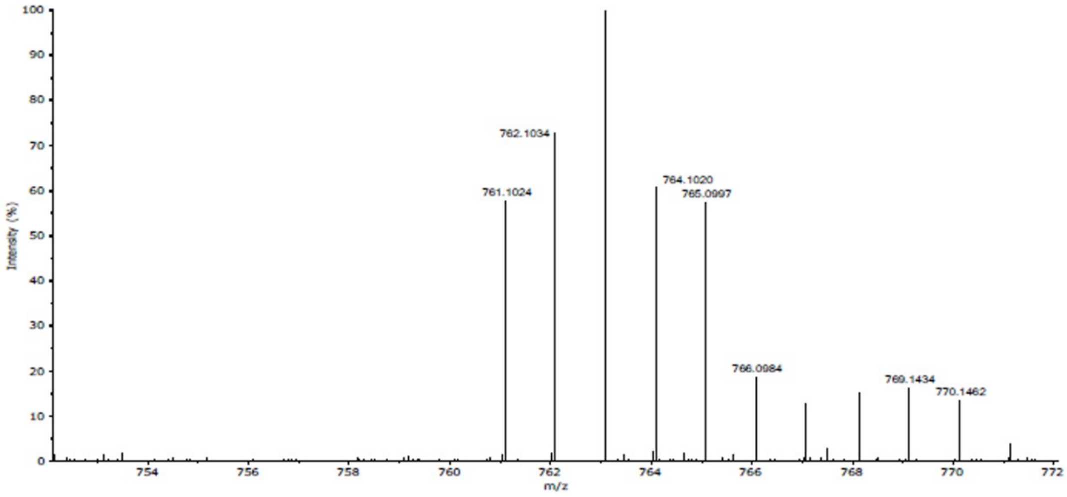

Theoretical Spectrum for C25H29Cl2N4O7Pt, Minimum Abundance 0.01%

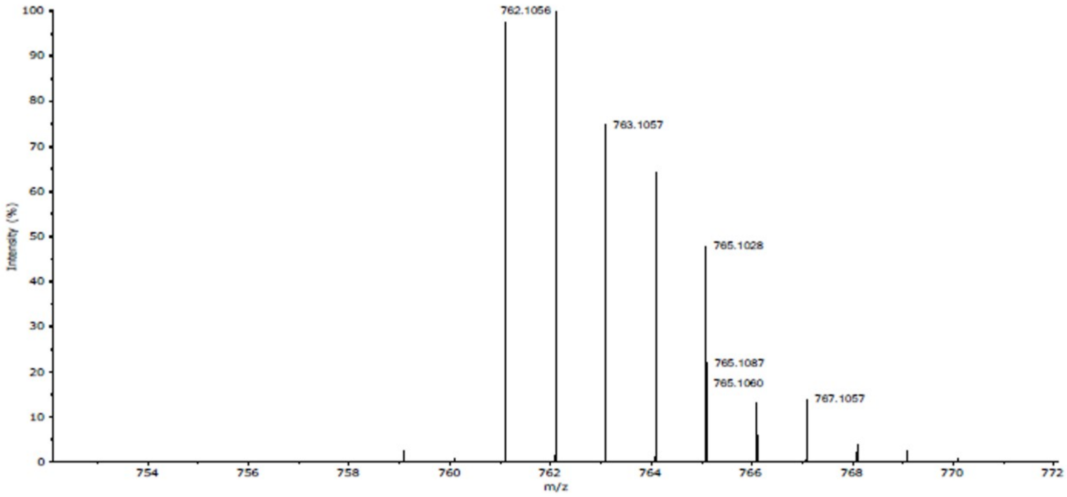

Measured Mass	Calculated Mass	Error (mDa)	Error (ppm)	Formula $[M+H]^+$	Response
762.1034	762.1056	-2.17	-2.85	$C_{25}H_{29}Cl_2N_4O_7Pt$	287858

The measured m/z value is consistent with your proposed formula for this sample.

Figure S56. HRMS of **CisBlue** (H adduct, + ion mode) in MeOH (+ 1% DMSO).

5. Computational Analysis

All calculations were carried out using Gaussian 16.[6] using the PBE1PBE functional and the –D3 dispersion correction of Grimme et al.[7] in combination with the basis sets 6-31G**(H,C,N,O)/LANL2DZ(Pt)[8–12] and an integral equation formalism SCRF continuum solvent method with continuum parameters designed to describe dimethylformamide solvent.[13] Multiple conformers of the Pt(IV)L_6 complexes **CarboNap** and **CarboBlue** were optimised in Gaussian to confirm the lowest energy structures. The lowest energy conformers were optimised and subsequently confirmed to be energetic minima via the absence of imaginary frequencies in the vibrational calculations. The valence orbitals of **CarboNap** and **CarboBlue** are presented in **Figure S57**. For both molecules, the LUMO is largely Pt dz^2 in character. The HOMO in **CarboNap** is a Nap-based π orbital, and in **CarboBlue** is a π-orbital localised around the -$PhCH_2O$- moiety.

TD-DFT calculations were performed on the optimised structures of **CarboNap** and **CarboBlue** with PBE1PBE/6-31G**(H,C,N,O)/LANL2DZ(Pt)/gd3 in DMF as solvent, using 30 roots (the first 10 excitations are detailed in **Table S1**). The visible region of the spectrum was well reproduced for **CarboNap** (**Figure S58**), but less so for **CarboBlue** (**Figure S59**). For the optimised structure of **CarboNap**, the excited states 1 (433 nm) and 3 (371 nm) reproduces the broad absorbance at 362 nm well and are comprised of a HOMO -> LUMO and HOMO -> LUMO+2 transition, respectively. The comparable excitations (states 1 and 6) at 415 and 341 nm respectively for **CarboBlue** correspond to the HOMO -> LUMO and HOMO–1 -> LUMO+1 transitions respectively. To visualise these excitations, the natural transition orbitals (NTOs) were calculated for these significant excitations (oscillator strength (f) > 0.03). Excited state 1 of both **CarboNap** and **CarboBlue** corresponds to a ligand-to-metal charge transfer (LMCT) involving a ligand-based π orbital and the Pt dz^2-based orbital (**Figures S60-S61**). Excited state 3 in **CarboNap** and excited state 6 in **CarboBlue** both involve a π-π* transition of the Nap moiety in both molecules (**Figures S62**-**S63**).

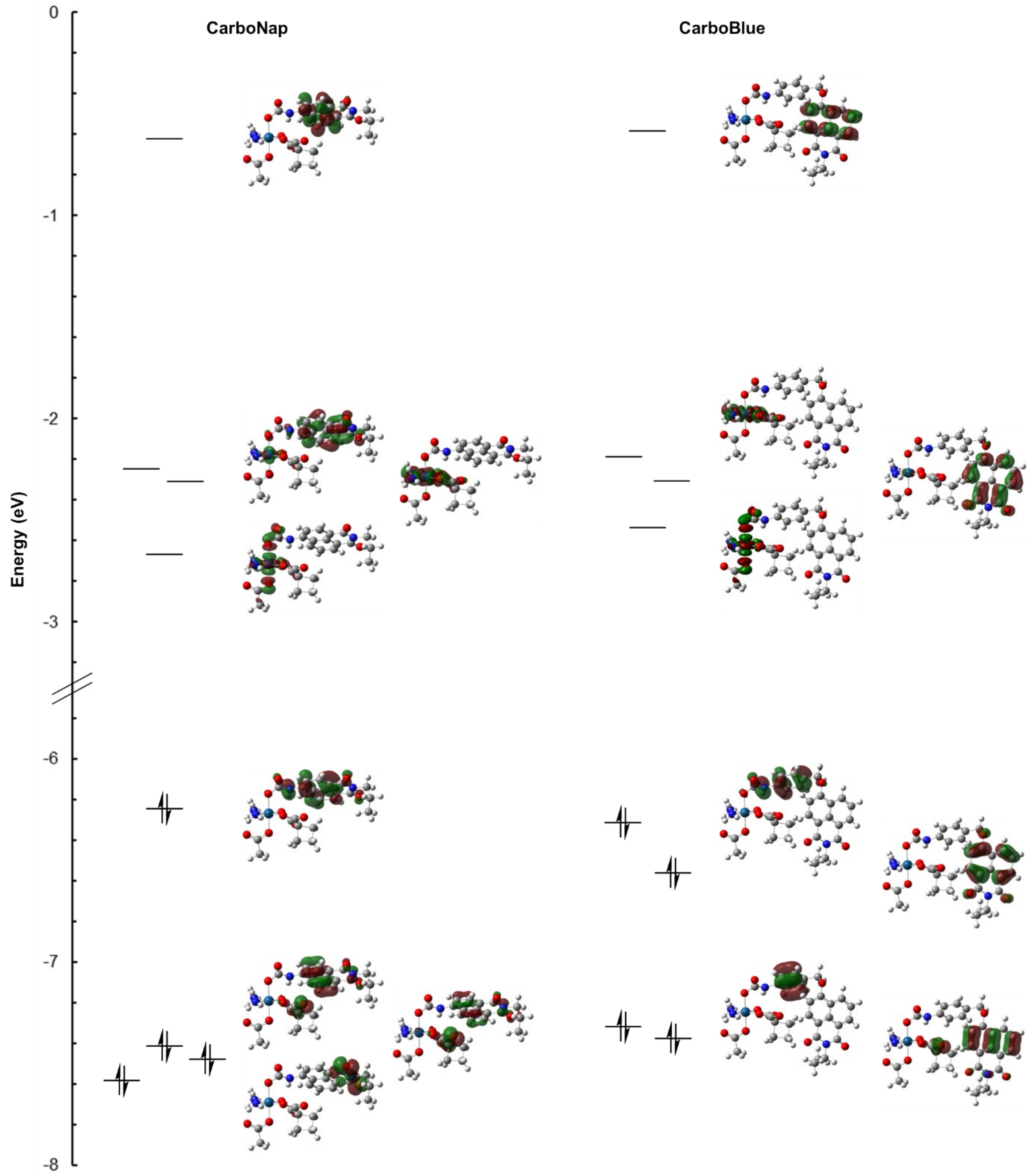

Figure S57. Frontier Kohn-Sham molecular orbitals for **CarboNap** (left) and **CarboBlue** (right). Isodensity value 0.04 au.

Table S1. Summary of the ten computed lowest energy singlet excited states of **CarboNap** and **CarboBlue**. Those with oscillator strength (f) > 0.03 are highlighted.

	**CarboNap**			**CarboBlue**		
**Excitation**	nm	Oscillator strength (f)		nm	Oscillator strength (f)	
**1st**	**433.20**	**0.0718**	**HOMO -> LUMO**	**414.96**	**0.0324**	**HOMO -> LUMO**
**2nd**	385.37	0.0054		372.31	0.0004	
**3rd**	**370.68**	**0.3201**	**HOMO -> LUMO+2**	363.82	0.0063	
**4th**	352.50	0.0016		355.64	0.0042	
**5th**	325.56	0.0051		344.25	0.0062	
**6th**	314.02	0.0046		**340.71**	**0.2649**	**HOMO–1 -> LUMO+1**
**7th**	310.62	0.0001		317.44	0.0089	
**8th**	304.50	0.0133		314.69	0.0013	
**9th**	303.76	0.0022		312.11	0.0040	
**10th**	297.43	0.0052		309.82	0.0003	

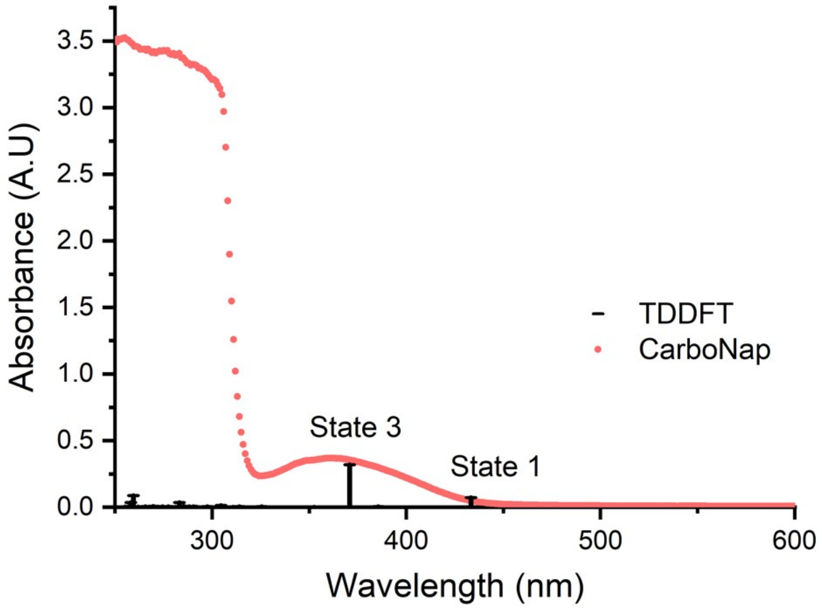

Figure S58. Experimental UV-vis spectra for **CarboNap** (red) overlayed with calculated transitions (black bars) from TDDFT calculations.

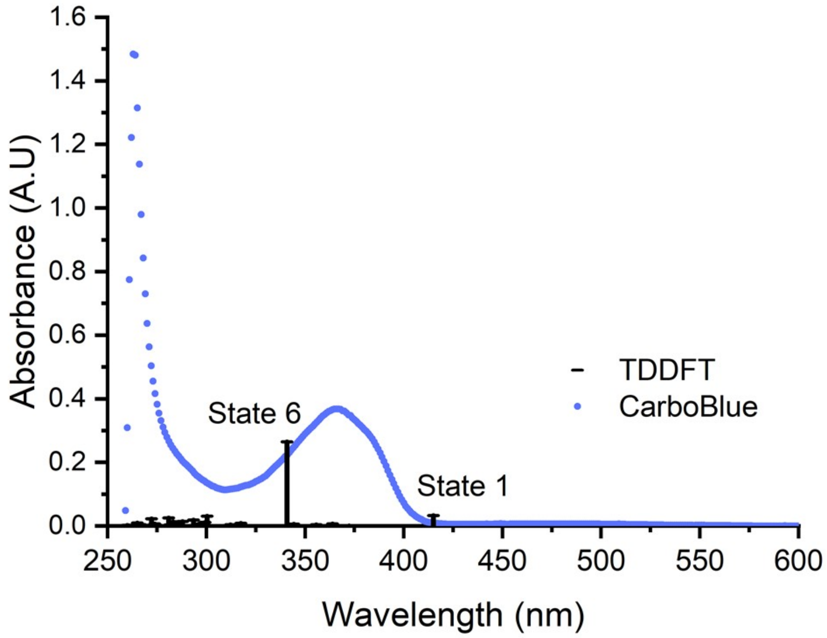

Figure S59. Experimental UV-vis spectra for **CarboBlue** (blue) overlayed with calculated transitions (black bars) from TDDFT calculations.

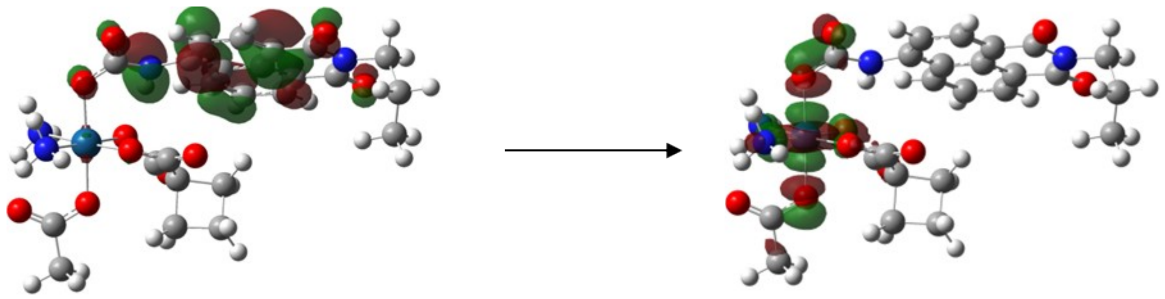

Figure S60. Natural Transition Orbitals (NTOs) for the dominant transition of the first excitation of **CarboNap** (433 nm) at PBE1PBE/6-31G**(C,H,N,O)LANL2DZ(Pt).

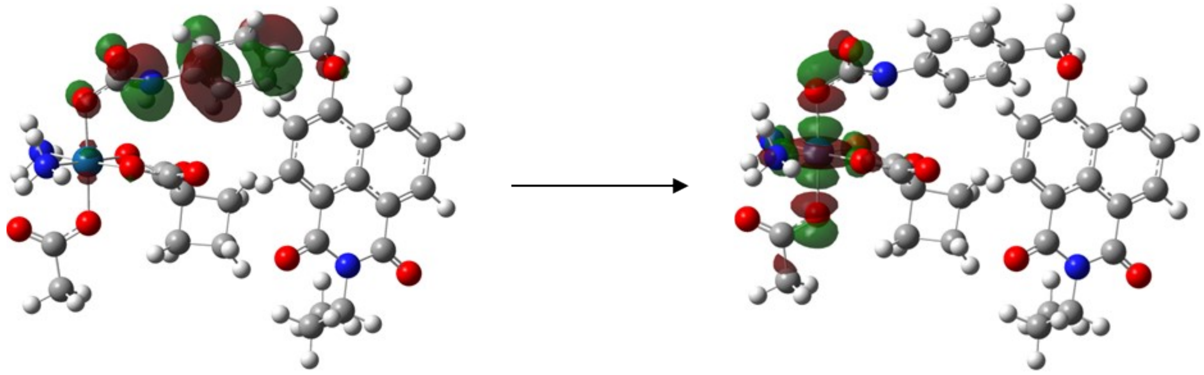

Figure S61. Natural Transition Orbitals (NTOs) for the dominant transition of the first excitation of **CarboBlue** (414.96 nm) at PBE1PBE/6-31G**(C,H,N,O)LANL2DZ(Pt).

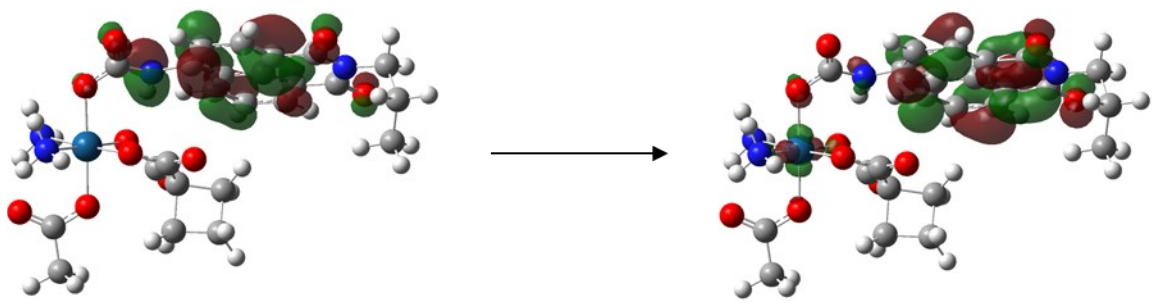

Figure S62. Natural Transition Orbitals (NTOs) for the dominant transition of the third excitation of **CarboNap** (371 nm) at PBE1PBE/6-31G**(C,H,N,O)LANL2DZ(Pt).

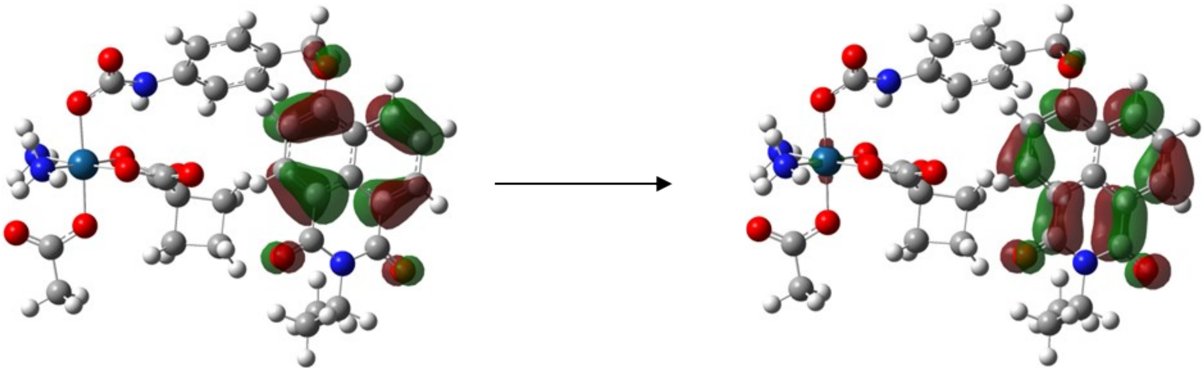

Figure S63. Natural Transition Orbitals (NTOs) for the dominant transition of the sixth excitation of **CarboBlue** (340.71 nm) at PBE1PBE/6-31G**(C,H,N,O)LANL2DZ(Pt).

CarboNap

Pt	-2.97116100	-0.48702400	-0.00613300
N	-4.37531800	-0.70079700	1.48415500
H	-4.49459200	-1.69306600	1.72722900
H	-4.02811900	-0.18618000	2.30612700
N	-4.22575100	-1.27810800	-1.41799800
H	-3.80093700	-1.12206600	-2.34320000
H	-4.32851800	-2.28929100	-1.25646300
O	-1.60568400	-0.36207800	-1.44015000
O	0.47204000	0.20731500	-2.01086100
O	-1.75260800	0.27819200	1.38893600
O	-1.04733200	2.36226500	1.87047900
O	-2.39907300	-2.37921500	0.35286500
O	-0.79447900	-3.78334600	-0.42164400
O	-3.55641800	1.40657800	-0.36301000
O	-5.76087300	0.82592400	-0.42841300
C	-0.50128500	0.41240300	-1.28487000
C	-0.55232500	1.59215600	-0.33783200
C	-1.14648700	1.46712100	1.03925400
C	-1.13782700	2.81854100	-1.16033300
H	-1.85369900	2.56336200	-1.94091900
H	-1.57048500	3.52424700	-0.44643800
C	0.31965400	3.21083700	-1.54703000
H	0.60857200	2.75371400	-2.49611400
H	0.56624200	4.27357900	-1.53148500
C	0.82298100	2.33994700	-0.35484300
H	0.91131400	2.92409900	0.56278800
H	1.68757100	1.69729900	-0.51746200
C	-1.09812500	-2.75038300	0.17872600
C	-4.85809800	1.69209400	-0.49628000
C	-5.09107400	3.15777900	-0.73065900
H	-4.58081800	3.46062100	-1.65113300
H	-4.65386300	3.72827800	0.09516200
H	-6.16007300	3.35517900	-0.80736700
H	-5.25569700	-0.28466100	1.13186800
H	-5.12500000	-0.76892200	-1.32882400
N	-0.19684600	-1.87875200	0.77360100
H	-0.63636000	-1.07885000	1.25182400
C	1.16784800	-1.83604000	0.55803400
C	1.91550600	-0.78373800	1.19888900
C	1.83296200	-2.71704500	-0.29223100
C	3.27654900	-0.59186900	0.82753500
C	1.36363500	0.07924600	2.17638100
C	3.18127700	-2.53097700	-0.60376500
H	1.26812600	-3.52669600	-0.72774600
C	4.00454700	0.48748600	1.37688800
C	3.89916100	-1.47098800	-0.08373900
C	2.09447000	1.12806600	2.69941200
H	0.35976400	-0.08296600	2.54905100
H	3.68295500	-3.20751200	-1.28683400
C	5.29258100	-1.27899500	-0.48240800
H	1.63874300	1.78526500	3.42990400
C	5.40513300	0.72529200	0.98253000
C	3.41837600	1.34449700	2.28601900
H	4.00568300	2.16730800	2.67662200
N	5.95637100	-0.18307000	0.08046100
O	5.88568300	-2.02569900	-1.27996500
O	6.06713500	1.68006000	1.42338300
C	7.34565100	0.04892200	-0.36486800
H	7.79181300	0.72259600	0.36853400
H	7.86000500	-0.91514000	-0.36335400
C	7.37575600	0.67549900	-1.76505500
H	8.42402400	0.88391200	-2.01562400
H	6.99542800	-0.06220600	-2.48034100
C	6.54641300	1.96575800	-1.83117900
H	5.48009800	1.73829900	-1.71564400
H	6.83397900	2.64879300	-1.02399500
H	6.68444400	2.47032200	-2.79377600

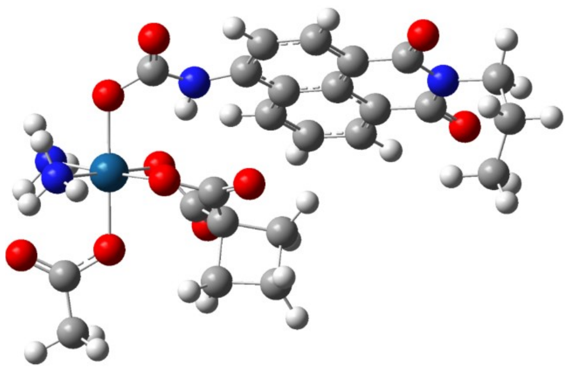

CarboBlue

Pt	3.89779300	-0.09340700	-0.13772900
N	5.64030400	0.01908000	0.94820200
H	6.12343400	0.90650200	0.75477400
H	5.38696500	-0.02285500	1.94559100
N	4.90380500	-0.37344200	-1.89918500
H	4.21408700	-0.64609100	-2.61384400
H	5.35044100	0.51121400	-2.17633900
O	2.24849500	-0.12255300	-1.23954700
O	0.04400700	0.06505600	-1.39071900
O	2.95571400	0.19680600	1.59961300
O	1.33492800	-0.41178700	3.00144500
O	4.08934000	1.87018300	-0.51688200
O	2.83803200	3.45166200	-1.56709400
O	3.75160900	-2.07479000	0.21561600
O	5.93236200	-2.44177800	-0.34794500
C	1.01801000	-0.22806400	-0.69252900
C	0.87362600	-0.83193700	0.68887300
C	1.73162200	-0.35466600	1.83630100
C	0.88932100	-2.41987700	0.50153300
H	1.43996500	-2.77437800	-0.36826600
H	1.27591900	-2.88490700	1.41176100
C	-0.66745600	-2.39427800	0.46308400
H	-1.06717300	-2.37784300	-0.55127400
H	-1.17672600	-3.16021200	1.05252000
C	-0.63087400	-0.95963900	1.07462000
H	-0.70715800	-0.97729300	2.16294800
H	-1.28344400	-0.20857000	0.63277100
C	2.97826200	2.66881300	-0.62270700
C	4.81562600	-2.86452200	0.03685400
C	4.51110000	-4.30285300	0.35033200
H	3.71573600	-4.65507900	-0.31511200
H	4.14300800	-4.37832100	1.37853300
H	5.40839300	-4.90768100	0.22091700
H	6.21464300	-0.79949200	0.67574100
H	5.59162600	-1.13117400	-1.73113700
N	2.10618700	2.52446400	0.43151800
H	2.40857200	1.86724400	1.15797000
C	-3.98977100	2.42063500	-0.59863600
C	-5.16872200	1.69342700	-0.26207100
C	-2.88555300	1.75101600	-1.08879900
C	-5.18567300	0.28508700	-0.45210900
C	-6.32153500	2.33053800	0.25219400
C	-2.91919600	0.36088700	-1.28704600
H	-1.97842100	2.29700500	-1.30638000
C	-6.33653300	-0.45018800	-0.09591600
C	-4.04827600	-0.36670300	-0.97391300
C	-7.43881400	1.59188100	0.58700400
H	-6.30191100	3.40746400	0.36861100
H	-2.02755000	-0.14127900	-1.64005400
C	-4.05138500	-1.81806200	-1.17392400
H	-8.31816500	2.08581500	0.98316100
C	-6.35853200	-1.91555000	-0.24699900
C	-7.44444400	0.19344400	0.41881600
H	-8.31053700	-0.40070500	0.68731700
N	-5.20608500	-2.49910600	-0.78281200
O	-3.09061900	-2.44598100	-1.65435500
O	-7.33505400	-2.60778500	0.08322700
C	-5.17180300	-3.97243600	-0.89654400
H	-6.20712200	-4.30625500	-0.81153100
H	-4.76835000	-4.22238000	-1.88082500
C	-4.31047600	-4.58819000	0.21486200
H	-4.38806100	-5.67991200	0.13095400
H	-3.26592800	-4.30675800	0.04212600
C	-4.76294900	-4.12728600	1.60738500
H	-4.55805700	-3.05816400	1.73819700
H	-5.84064400	-4.28270700	1.73302800
H	-4.22933000	-4.67428400	2.39243900
C	0.28256600	3.97525000	-0.37219000
C	-1.03166700	4.40202700	-0.23773300
C	-1.31928700	2.99381100	1.68802800
C	-0.00141000	2.56914300	1.57737300
C	0.81224600	3.03976400	0.53340700
H	0.91672000	4.33068100	-1.17079000
H	-1.44091400	5.10507900	-0.95733900
H	-1.94604800	2.58617200	2.47529200
H	0.38425300	1.83544800	2.27809300
C	-1.85067100	3.91672000	0.78663700
C	-3.30330500	4.27693900	0.81249400
H	-3.79791100	3.85318300	1.69383400
H	-3.46606500	5.35618300	0.77773300
O	-4.00829700	3.79501100	-0.41894600

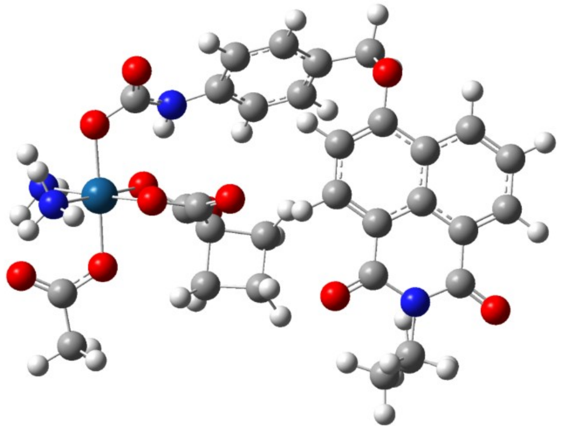

6. References

1. Li, J. *et al.* Ratiometric fluorescent probes for ClO− and in vivo applications. *Dyes Pigm.* **130**, 209–215 (2016).

2. Marsh, J. W. *et al.* Fluorogenic platinum(IV) complexes as potential predictors for the design of hypoxia-activated platinum(IV) prodrugs. *Dalton Trans.* **53**, 14811–14816 (2024).

3. Rudebeck, E. E. *et al.* Mixed alkoxy/hydroxy 1,8-naphthalimides: expanded fluorescence colour palette and in vitro bioactivity. *Chem. Commun.* **56**, 6866–6869 (2020).

4. Boulet, M. H. C., Bolland, H. R., Hammond, E. M. & Sedgwick, A. C. Oxali(IV)Fluors: Fluorescence Responsive Oxaliplatin(IV) Complexes Identify a Hypoxia-Dependent Reduction in Cancer Cells. *J. Am. Chem. Soc*. **145**, 12998–13002 (2023).

5. Jones, W. E. & Fox, M. A. Determination of Excited-State Redox Potentials by Phase-Modulated Voltammetry. *J. Phys. Chem* **98**, 5095–5099 (1994).

6. Carpenter, J. E. & Weinhold, F. Analysis of the geometry of the hydroxymethyl radical by the 'different hybrids for different spins' natural bond orbital procedure. *Journal of Molecular Structure: THEOCHEM* **169**, 41–62 (1988).

7. Grimme, S., Antony, J., Ehrlich, S. & Krieg, H. A consistent and accurate ab initio parametrization of density functional dispersion correction (DFT-D) for the 94 elements H-Pu. *J. Chem. Phys.* **132**, 14 (2010).

8. Hay, P. J. & Wadt, W. R. Ab initio effective core potentials for molecular calculations. Potentials for the transition metal atoms Sc to Hg. *J. Chem. Phys.* **82**, 270–283 (1985).

9. Hay, P. J. & Wadt, W. R. Ab initio effective core potentials for molecular calculations. Potentials for K to Au including the outermost core orbitale. *J. Chem. Phy.s* **82**, 299–310 (1985).

10. Binkley, J. S., Pople, J. A. & Hehre, W. J. Self-consistent molecular orbital methods. 21. Small split-valence basis sets for first-row elements. *J. Am. Chem. Soc.* **102**, 939–947 (1980).

11. Pietro, W. J. *et al.* Self-Consistent Molecular Orbital Methods. 24. Supplemented Small Split-Valence Basis Sets for Second-Row Elements. *J. Am. Chem. Soc.* **104**, 5039–5048 (1982).

12. Gordon, M. S., Binkley, J. S., Pople, J. A., Pietro, W. J. & Hehre, W. J. Self-Consistent Molecular-Orbital Methods. 22. Small Split-Valence Basis Sets for Second-Row Elements. *J. Am. Chem. Soc.* **104**, 2797–2803 (1982).

13. Tomasi, J., Mennucci, B. & Cammi, R. Quantum mechanical continuum solvation models. *Chem. Rev.* **105**, 2999–3093 (2005).
